# Natural Glycoforms of Human Interleukin 6 Show Atypical Plasma Clearance

**DOI:** 10.1002/anie.202101496

**Published:** 2021-05-06

**Authors:** Andreas Reif, Kevin Lam, Sascha Weidler, Marie Lott, Irene Boos, Juliane Lokau, Christian Bretscher, Manuel Mönnich, Lukas Perkams, Marina Schmälzlein, Christopher Graf, Jan‐Patrick Fischer, Carolin Lechner, Kerstin Hallstein, Stefan Becker, Michael Weyand, Clemens Steegborn, Gerhard Schultheiss, Stefan Rose‐John, Christoph Garbers, Carlo Unverzagt

**Affiliations:** ^1^ Bioorganic Chemistry University of Bayreuth Universitätsstraße 30 95447 Bayreuth Germany; ^2^ Department of Pathology Medical Faculty Otto von Guericke University Magdeburg 39120 Magdeburg Germany; ^3^ Protein Engineering & Antibody Technologies Merck Healthcare KGaA Frankfurter Str. 250 64293 Darmstadt Germany; ^4^ Department of Biochemistry Kiel University 24098 Kiel Germany; ^5^ Department of Biochemistry University of Bayreuth Universitätsstraße 30 95447 Bayreuth Germany; ^6^ Animal Welfare Kiel University 24098 Kiel Germany

**Keywords:** glycopeptides, glycoproteins, native chemical ligation, oligosaccharides, serum clearance

## Abstract

A library of glycoforms of human interleukin 6 (IL‐6) comprising complex and mannosidic N‐glycans was generated by semisynthesis. The three segments were connected by sequential native chemical ligation followed by two‐step refolding. The central glycopeptide segments were assembled by pseudoproline‐assisted Lansbury aspartylation and subsequent enzymatic elongation of complex N‐glycans. Nine IL‐6 glycoforms were synthesized, seven of which were evaluated for in vivo plasma clearance in rats and compared to non‐glycosylated recombinant IL‐6 from E. coli. Each IL‐6 glycoform was tested in three animals and reproducibly showed individual serum clearances depending on the structure of the N‐glycan. The clearance rates were atypical, since the 2,6‐sialylated glycoforms of IL‐6 cleared faster than the corresponding asialo IL‐6 with terminal galactoses. Compared to non‐glycosylated IL‐6 the plasma clearance of IL‐6 glycoforms was delayed in the presence of larger and multibranched N‐glycans in most cases

## Graphical abstract

Sugars affect plasma lifetime: The main glycoforms of human interleukin 6 (IL‐6) were synthesized by native chemical ligation combining chemical, enzymatic, and recombinant methods. The native fold was evident from CD spectra, mass spectrometry, crystallography, and receptor binding. In vivo assays showed a different plasma clearance for each glycoform with an unexpected correlation to the glycan structures.

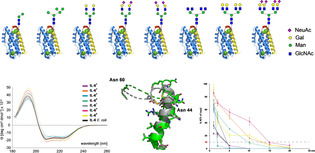

## Introduction

Although many of the biological effects of the human cytokine interleukin 6 (IL‐6) have been studied in detail,^[1]^ little is known about the influence of the glycans present on this glycoprotein. The glycan analysis of IL‐6 isolated from induced human blood monocytes revealed a relatively small set of N‐glycans, which were separated by gel filtration and identified by glycosidase digestion.^[2]^ The biological activity of a glycoprotein is typically modified by the sugar part,^[3]^ however, homogenous glycoproteins (glycoforms) are rarely accessible from natural sources and need to be accessed by synthesis.^[4]^ Following our semisynthetic approach to biologically active IL‐6 glycoproteins^[5]^ we planned to synthesize a representative set of glycans of IL‐6 and generate the corresponding IL‐6 glycoforms. Here we show for the first time that each IL‐6 glycoform has a different plasma half‐live showing either an increased or decreased clearance relative to non‐glycosylated IL‐6 from *E. coli*.

IL‐6 is a cytokine exerting both immunostimulating and regenerating effects depending on the localization of the IL‐6 receptor.^[6]^ In vivo, IL‐6 is mainly targeted to the liver^[7]^ but can also be complexed by a soluble, circulating IL‐6 receptor.^[8]^ When assayed with cells depending on IL‐6 as a proliferation stimulus the bioactivity of two IL‐glycoforms synthesized initially^[5]^ was identical to non‐glycosylated IL‐6 from *E. coli*. We thus concluded that the N‐glycan of IL‐6 does not affect binding to the cellular IL‐6 receptor. On the other hand, the serum half‐life of IL‐6 in vivo should depend on the type of oligosaccharide. Based on the glycan structures identified earlier^[2]^ we set out to provide a comprehensive library of IL‐6 glycoforms including complex‐type and oligomannosidic N‐glycans for systematic studies.

**Scheme 1:**
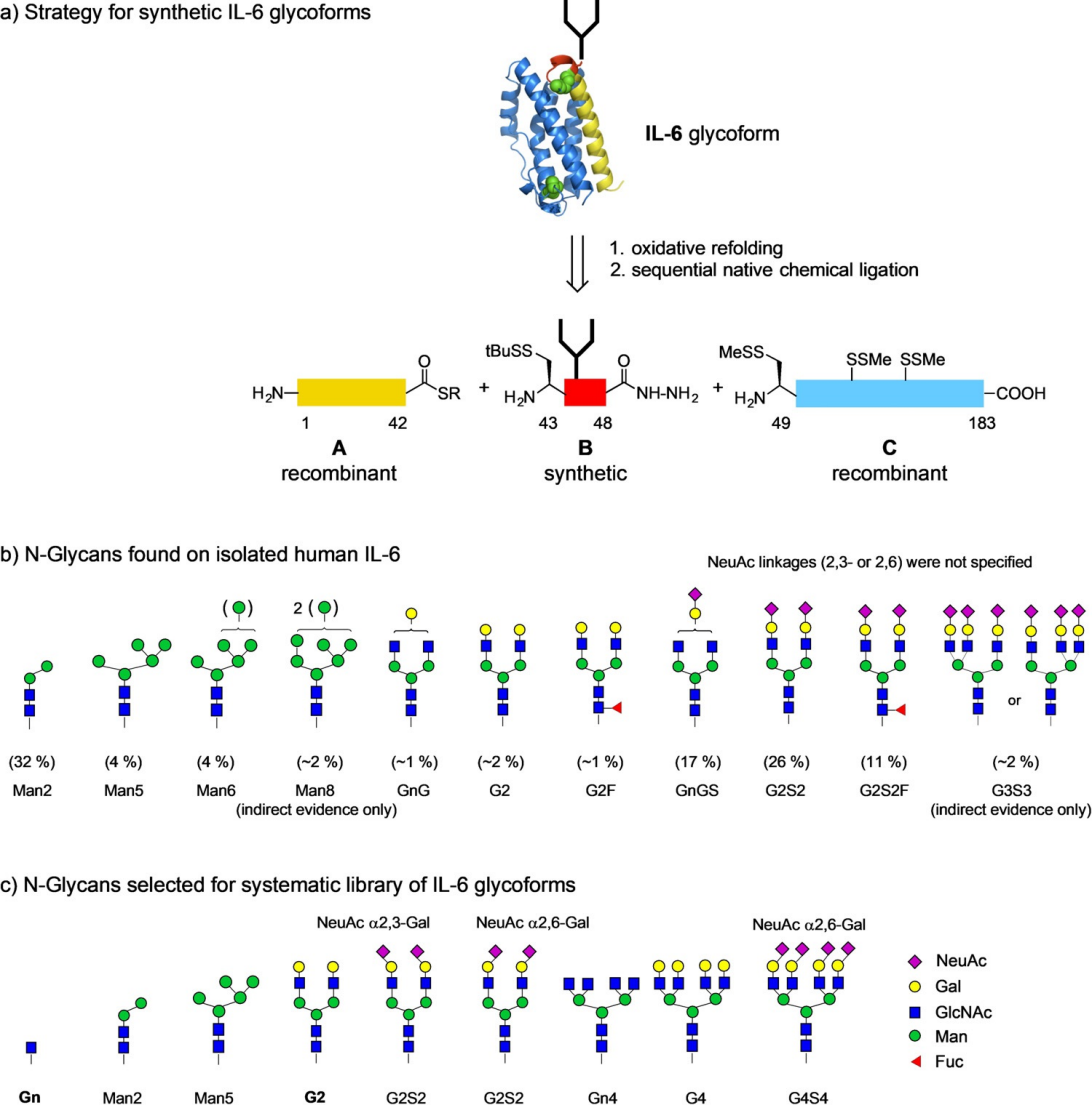
a) Retrosynthesis of IL‐6 glycoforms; b) N‐glycans detected in natural human IL‐6 (values in parentheses give percentage of total N‐glycans and were deduced from ref. [2]); c) structures of N‐glycans envisioned for systematically varied library of hIL‐6 glycoforms. The IL‐6 glycoforms marked in bold (**Gn** and **G2**) were available from previous work.^[5]^

The main N‐glycans identified on human IL‐6^[2]^ isolated from mononuclear cells (Scheme 1 b) were oligomannosidic (40 %) or complex type (53 % sialylated and ≈6 % neutral). Among the mannosidic N‐glycans an unusual paucimannosidic Man2 tetrasaccharide was predominant (32 %) followed by a Man5 and a Man6 glycan (4 % each) and traces of Man8. The main complex type N‐glycan was biantennary and sialylated (26 %). The closely related core‐fucosylated or monogalactosylated biantennary N‐glycans were less abundant (11–17 %). Only traces of a presumably triantennary complex N‐glycan were found (2 %).

The structures envisioned for the library of synthetic IL‐6 glycoforms are shown in Scheme 1 c. We focused on the most abundant N‐glycans of each subtype and thus selected Man2 and Man5 from oligomannosidic structures as well as G2S2 from the complex type. Since the type of linkage of the terminal sialic acids was not specified, we envisioned the biantennary structures G2S2 in the 2,3‐ and the 2,6‐sialylated form. The effect of desialylation on IL‐6 should be addressed with the G2 glycan, which was already available as an IL‐6 glycoform.^[5]^

The core‐fucosylated or monogalactosylated biantennary N‐glycans were not implemented in this study due to their high similarity to the major biantennary N‐glycan. The low abundance triantennary N‐glycan was not structurally defined and may be branched within the α1,3‐ or the α1,6‐arm. To consider both possibilities and to generally investigate the effect of additional N‐glycan branches on IL‐6 we decided to incorporate tetraantennary N‐glycans with terminal GlcNAc (Gn4), Gal (G4) or 2,6‐linked sialic acid residues (G4S4) as a surrogate to maximize potential steric and multivalency effects in combination with sialylation/desialylation (Scheme 1 c).

## Results and Discussion

**Scheme 2:**
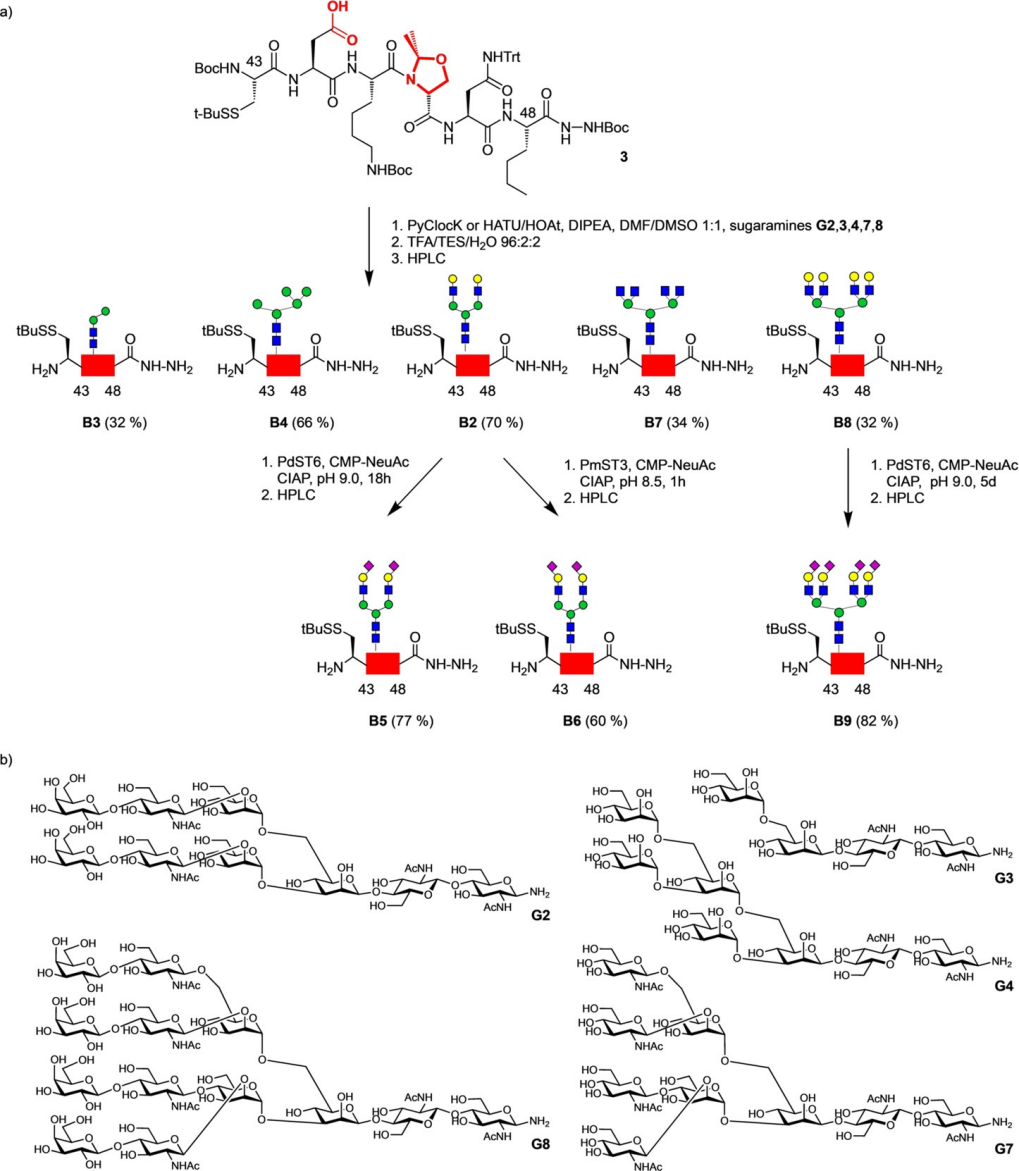
a) Chemical and enzymatic synthesis of IL‐6 glycopeptides **B2**–**B9**; b) glycosylamines **G2**, **G3**, **G4**, **G7**,and **G8** employed for coupling with **3**.

For the semisynthesis of the library of IL‐6 glycoforms^[5]^ three segments (**A**–**C**) were employed (Scheme 1 a). The functionalized segments **A** and **C** were obtained recombinantly and the short glycopeptide segment **B** was synthesized convergently by pseudoproline‐assisted Lansbury aspartylation.^[9]^ The required hexapeptide hydrazide^[10]^ **3** (Scheme 2 a) was assembled by Fmoc‐SPPS and modified after cleavage from the resin. In segment **B** the N‐terminal cysteine was protected by a mixed disulfide and the native methionine at position 48 was replaced with a norleucine,^[11]^ thus preventing undesired oxidation.

Prior to coupling with aspartyl peptide **3** the glycosyl amines **G2**,**3**,**4**,**7**,**8** (Scheme 2 b) were freshly prepared by reduction of the corresponding azides.^[12]^ Except the biantennary compound^[9b]^ all N‐glycan azides were synthesized from modular building blocks^[13]^ followed by a multistep deprotection sequence yielding the desired unprotected N‐glycan azides. The key step in this sequence was an oxidative debenzylation selectively removing all four benzyl groups in the presence of the anomeric azide.[^12a^, ^14^]

For the paucimannosidic N‐glycan azide **7** (Scheme S8) a synthesis was developed based on the α‐selective glycosylation of a derivative of the core trisaccharide **5** with the disaccharide imidate **6** ^[15]^ The protected pentasaccharide **9** was deprotected to the corresponding free N‐glycan azide **10** followed by an enzymatic removal of the accessory GlcNAc moiety as the final step (see supplementary information). The disaccharide imidate **6** was preferred in this synthesis because the glycosylations of the primary hydroxyl group with peracetylated mannosyl donors gave rise to stable orthoesters, which were resistant to rearrangement to the desired α‐mannoside and gave low yields in the deprotection to the azide **7** (data not shown).

The coupling of the glycosyl amines **G2**,**3**,**4**,**7**,**8** was initiated by activating peptide **3** either with PyClock or HATU/HOAt followed by addition of the sugars. The crude products were deprotected and gave the glycopeptide hydrazides **B2**,**3**,**4**,**7**,**8** in yields of 32–70 % after RP‐HPLC. Due to the pseudoproline at Ser 45 the formation of aspartimides^[9]^ was reliably reduced. The sialylated glycopeptides **B5**,**6**,**9** were obtained by enzymatic sialylation^[16]^ of **B2** and **B8** using the bacterial sialyltransferases PdST6 or PmST3 and purified by RP‐HPLC. In all cases the sialylations required optimization of the reaction conditions. For the 2,3‐sialylation of **B2** small amounts of PmST3 and short reaction time were preferable whereas the 2,6‐sialylations using PdST6 required longer reaction times and repeated addition of CMP‐NeuAc. The final purification by RP‐HPLC readily separated intermediates with incomplete sialylation.

**Scheme 3:**
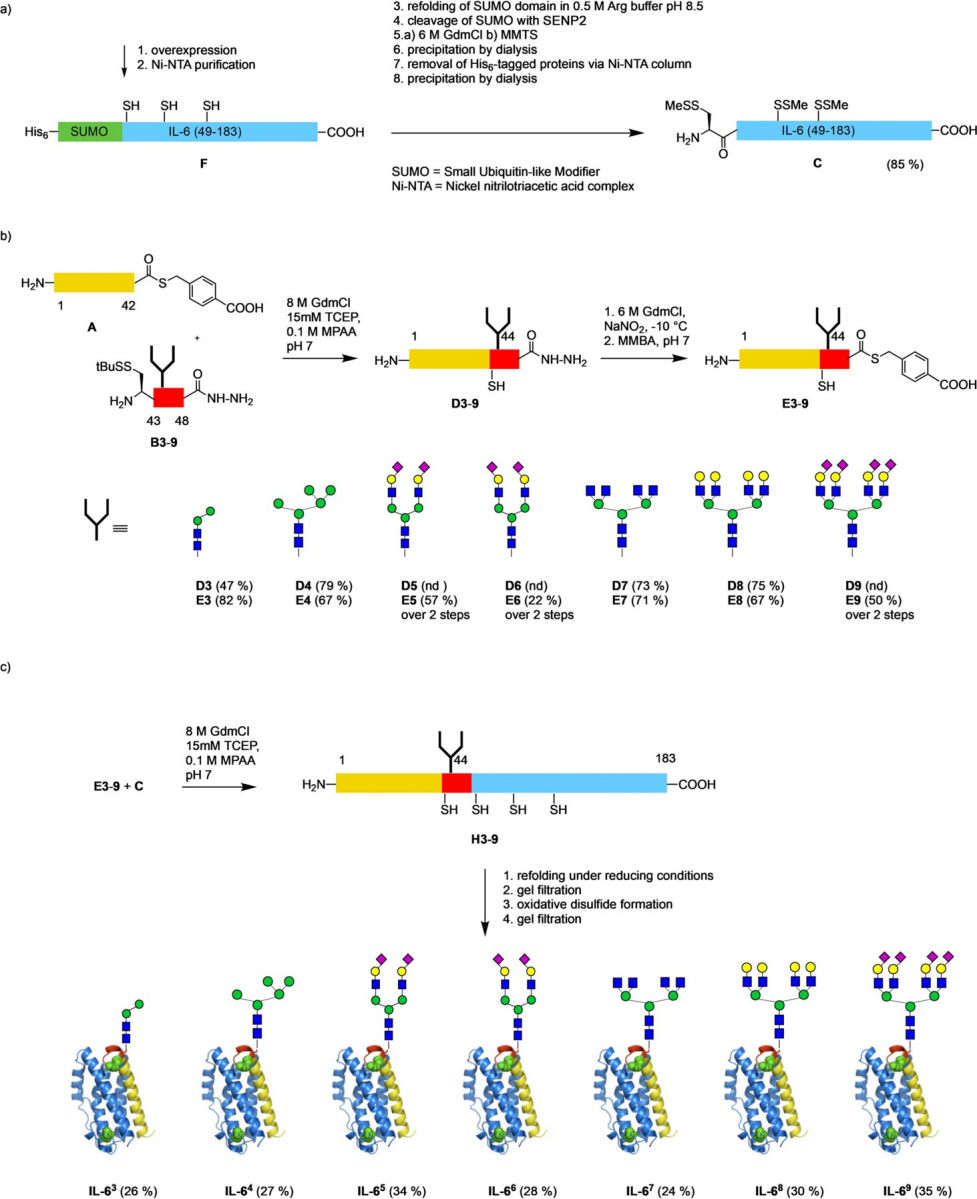
a) Recombinant expression of fusion protein **F** and conversion to disulfide‐protected fragment **C**; b) native chemical ligation of segments **A** and **B3**–**B9** to IL‐6 (1–48) hydrazides **D3**–**D9** and conversion to thioesters **E3**–**E9**; c) native chemical ligation of thioesters **E3**–**E9** with segment **C** followed by a two‐step refolding and oxidation of the full‐length glycopeptides **H3**–**H9** to the IL‐6 glycoforms **IL‐6^3^**–**IL‐6^9^**.

The recombinant IL‐6 peptide 49–183 is not compatible with the conditions for preparative purification by RP‐HPLC due to low recovery when applying acidic MeCN/water gradients.^[5]^ Furthermore, the Asp139‐Pro140 bond is labile under acidic conditions.^[17]^ We thus examined the use of the disulfide‐protected recombinant^[18]^ segment **C** (Scheme 3 a). The corresponding SUMO fusion protein **F** ^[5]^ was cleaved with the SUMO‐protease SENP2 and the free thiols were quantitatively converted to mixed disulfides using excess thiosulfonate MMTS in 6 M GdmCl. After the modification fragment **C** and other proteins except SUMO were precipitated by dialysis against water. The precipitate was dissolved in 6 M GdmCl and purified over a Ni‐NTA column, which retained the remaining His_6_‐tagged proteins (subtractive Ni‐IMAC). By adding cysteine as a scavenger in the proteolysis step the formation of N‐terminal thiazolidines on Cys 49 was efficiently blocked. This protocol eliminated the need for a subsequent acidic methoxyamine treatment, which previously gave rise to a cleavage product at the Asp‐Pro site within IL‐6 49–183.^[5]^ The N‐terminal 1–42 thioester **A** was obtained recombinantly from the corresponding two‐intein fusion protein.^[5]^

With all the segments in hand the native chemical ligations were carried out sequentially in the C‐terminal direction.^[5]^ Since the C‐terminal amino acid of thioester **A** is a threonine^[19]^ the ligations with the seven glycopeptide hydrazides **B3**–**B9** were kept in an anaerobic tent for 4–8 days. After purification by RP‐HPLC the 1–48 glycopeptide hydrazides **D3**–**9** were obtained in yields of 47–79 %. The sialylated ligation products **D5**, **D6**, **D9** were immediately neutralized after purification by RP‐HPLC with NH_4_HCO_3_ to prevent loss of sialic acids during or after lyophilization.

The 1–48 glycopeptide hydrazides **D3**–**9** were converted to the corresponding thioesters^[10]^ via diazotization followed by addition of the benzylthiol MMBA.^[5]^ Purification of the thioesters **E** by gel filtration was generally preferable over RP‐HPLC since the product mixtures contained varying amounts of thiolactone and mixed disulfide species. The seven glycopeptide thioesters **D3**–**9** were reacted with the protected segment **C** and the ligations to the full length‐IL‐6 glycopeptides **H3**–**9** were followed by LC‐MS for 5–10 d. Prior to refolding the ligation mixtures were reduced with DTT and then rapidly diluted under anaerobic conditions. The refolding mixture was subjected to a first gel filtration thereby removing oligomers and low molecular weight impurities. The disulfides of the refolded but still reduced IL‐6 glycoproteins (**IL‐6^3red^**–**IL‐6^9red^**, see supporting information) were subsequently oxidized in the presence of catalytic amounts of cysteamine. In a final gel filtration, newly formed oligomers were removed and the desired glycoforms **IL‐6^3^**–**IL‐6^9^** were obtained in high purity and good yields (24–35 %).

**Scheme 4:**
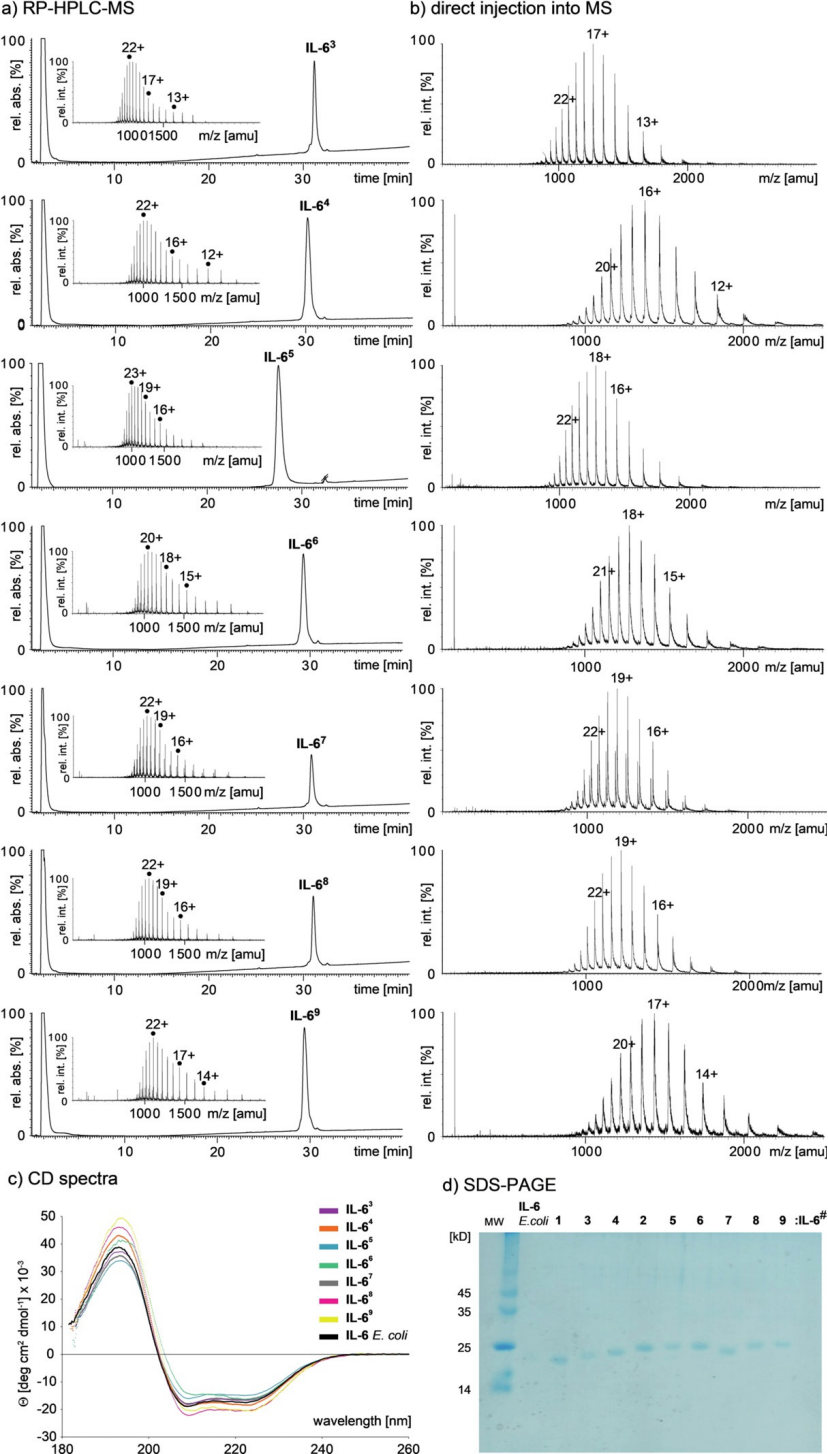
a) RP‐HPLC‐ESI‐TOF‐MS of glycoforms **IL‐6^3^**–**IL‐6^9^** using acetonitrile/water + 0.1 % HCOOH gradients. b) direct injections of desalted **IL‐6^3^**–**IL‐6^9^** (plain water) into ESI‐TOF mass spectrometer show gaussian distribution of charge states; c) overlay of the CD‐spectra of glycosylated **IL‐6^3^**–**IL‐6^9^**, d) SDS‐PAGE of glycoforms **IL‐6^1^**–**IL‐6^9^** (here termed **1**–**9**).

The glycoforms were characterized by LC‐MS, HR‐MS, SDS‐PAGE and CD‐spectroscopy indicating that the correct fold was attained and the formation of the disulfides was complete (Scheme 4).

IL‐6 is sensitive to partial denaturation by organic solvents,^[17]^ which accounts for the bimodal charge state distribution^[20]^ commonly observed during RP‐HPLC‐MS of the IL‐6 glycoforms **IL‐6^3^**–**IL‐6^9^** (Scheme 4 a). In contrast a gaussian charge state distribution was obtained (indicating a native fold of the glycoproteins)^[20]^ when injecting a desalted aqueous solution of the IL‐6 glycoforms directly into the mass spectrometer (Scheme 4 b).^[5]^ This pattern was consistent throughout the library of glycoforms. Additionally, an overlay showed that the CD‐spectra of the glycoforms **IL‐6^3^**–**IL‐6^9^** were very similar to that of the non‐glycosylated reference **IL‐6** *E. coli* (Scheme n n4 c). The native helical fold of the set of IL‐6 glycoforms was independent of the glycan and only the overall intensity of the spectra varied to a small extent (Scheme 4 c).

**Scheme 5:**
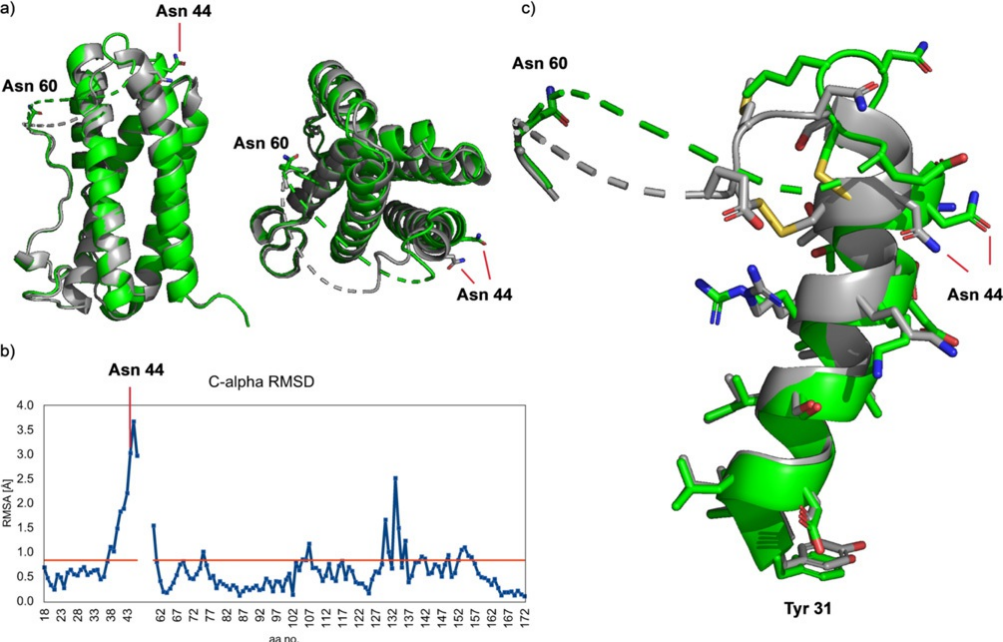
a) Structure superposition of glycosylated **IL‐6^1^** (PDB code 7NXZ, green) and non‐glycosylated IL‐6 (PDB code 1ALU, gray); b) Cα‐atom RMSD plot between both forms, showing the main deviations around the glycosylation site (Asn 44); c) enlargement of the Asn 44 glycosylation site containing helix A (Tyr 31 to Asn 44) showing the gradually increasing deviation towards and beyond the glycosylation site.

We also attempted to crystallize a synthetic IL‐6 glycoform and started with **IL‐6^1^**. After optimization of the crystallization conditions^[21]^ a crystal structure was obtained from **IL‐6^1^** bearing a single GlcNAc moiety, which was largely identical to the non‐glycosylated IL‐6 from *E. coli* (pdbID:1ALU) (Scheme 5 a). The crystallization of an IL‐6 glycoform with a full‐length N‐glycan (data not shown) was not successful. The structure of **IL‐6^1^** (GlcNAc) did not resolve the flexible loop beyond Glu 50 (Ser 51‐Asn 59) but refinement to 2.0 Å showed low electron density for the GlcNAc residue indicating connectivity at the side chain amide (see Figure S84). Notably, in both structures the unstructured loop region ends at Asn 60. However, the largest deviations between the two structures were observed around the glycosylation site at Asn44 reflecting an influence of the glycosylation on the orientation of helix A prior to the loop region (Scheme 5 b,c).

**Scheme 6:**
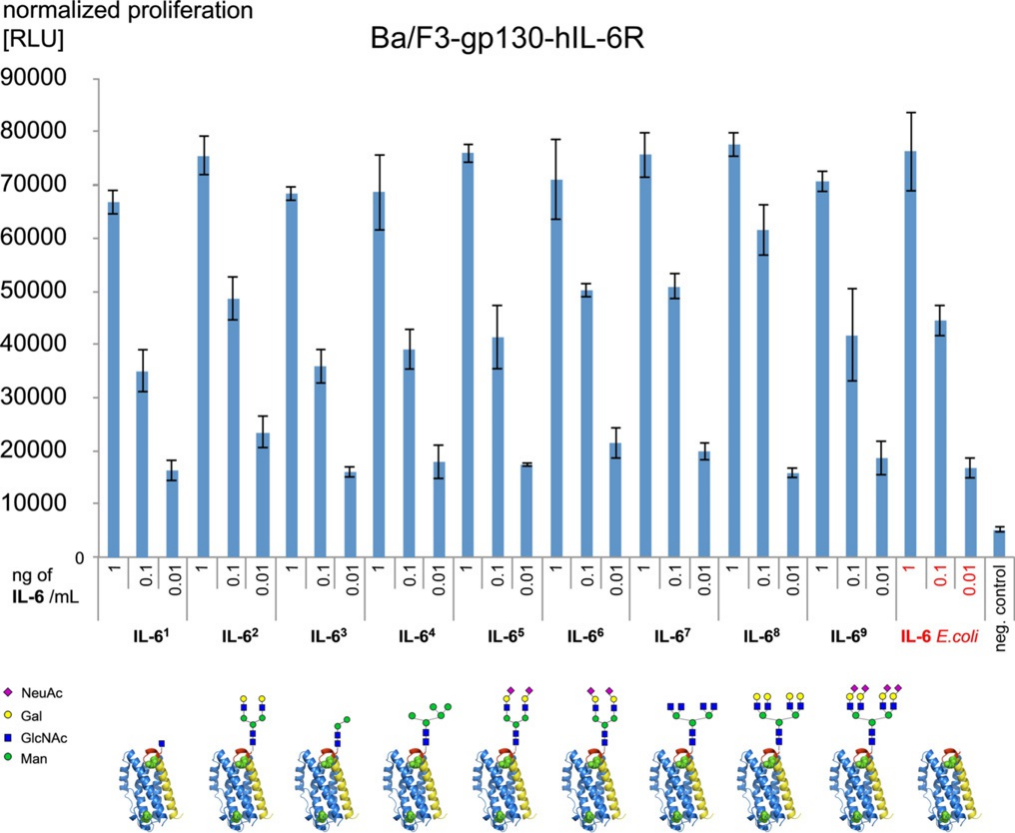
Proliferation assay of IL‐6 glycoforms **IL‐6^1^**–**IL‐6^9^** using an IL‐6‐dependent Ba/F3 cell line.

The biological activity of all glycoforms **IL‐6^1^**–**IL‐6^9^** was compared by a proliferation assay using the IL‐6‐dependent Ba/F3‐gp130‐hIL‐6R cell line^[5]^ (Scheme 6). Despite the variations in the sugar part the activity of the individual glycoforms was nearly identical in the cellular assays indicating that the binding to the IL‐6 receptor and the formation of the signal‐transducing receptor complex^[22]^ should not be significantly affected by the various N‐glycan structures.

To evaluate the biological activity of the library of IL‐6 glycoforms in the bloodstream we set out for an exploratory in vivo experiment with small rodents. The experimental design required optimization and was finally carried out with commercially available rats supplied with two implanted catheters suitable for injections directly into the bloodstream and repeated withdrawal of blood samples. Non‐glycosylated hIL‐6 expressed in *E. coli* is fully active in rats,^[23]^ known to have a short half‐life (≈3 min) in the blood stream of rats and is rapidly taken up by the liver or washed out by the kidney.^[7]^ We followed the unlabeled hIL‐6 glycoforms in the blood of the rats via an ELISA‐assay. To ascertain the unbiased detectability of each IL‐6 beforehand 250 pg of each glycoform was tested in the ELISA sandwich assay. The results were nearly identical for each IL‐6 variant indicating that the assay is not affected by the presence or the structure of the N‐glycans.

The individual dosage of each hIL‐6 variant was set to 8 μg per rat^[23b]^ thus ensuring a sufficient amount of detectable hIL‐6 in the serum. In total seven glycoforms were tested with IL‐6 *E. coli* serving as a reference. After injection of the IL‐6 in 250 μL of PBS the animals behaved normally and blood samples were taken over 20 minutes. Six blood samples (1–20 minutes) were analyzed by ELISA in triplicates and the residual amount of IL‐6 was plotted against time.

To assure that the measurements show minimal influence by animal‐to‐animal variability the regime of exposure to different IL‐6 glycoforms was as follows: A cohort of four rats was exposed to four different IL‐6 glycoforms and the response in the blood was measured. After a reconvalescence period of 7 days the same cohort was exposed to the remaining four glycoforms and after a second recovery period the first four glycoforms were administered again, but to different individuals. This set of experiments was repeated with a second cohort of animals allowing the measurement of a single glycoform in three individuals.

**Scheme 7:**
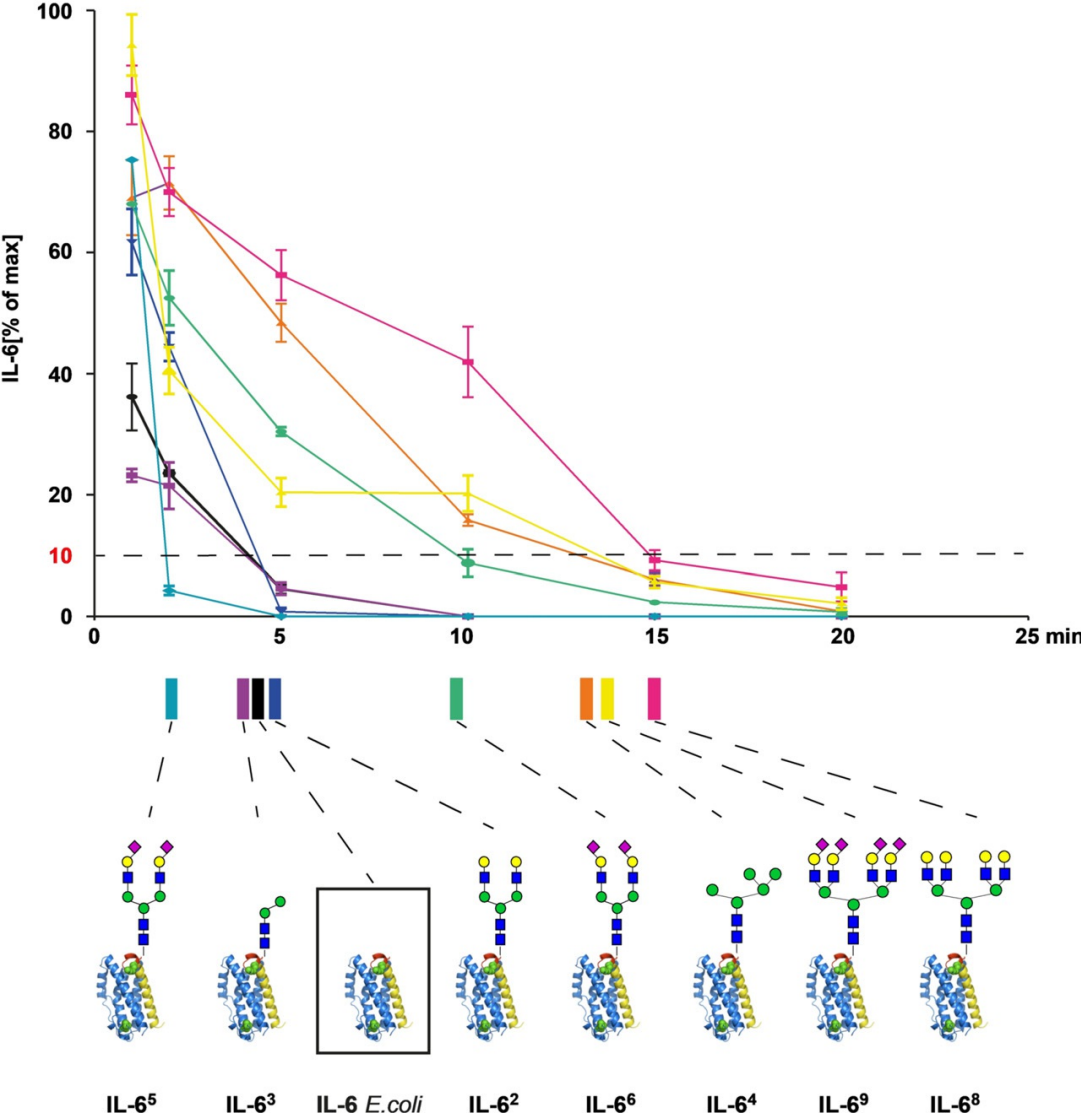
Normalized percentage of h**IL‐6** glycoforms detected in rat serum after IV injection. The 10 % of max. values were chosen arbitrarily for a ranking of the plasma clearance.

Strikingly, all the IL‐6 glycoforms tested showed different plasma clearance rates. Relative to the reference IL‐6 *E. coli* most glycoforms cleared slower, however, there were also two glycoforms with a faster clearance (Scheme 7). For better comparison of the intersecting curves we selected the 10 % of max value as a reference threshold. Since the measured clearances of the IL‐6 glycoforms were quite contrary to the expected ranking we reconfirmed by ESI‐MS that the samples used for testing were correctly assigned and administered.

For the sialylated IL‐6 glycoforms plasma clearance was quite diverse. The most rapidly disappearing compound was the biantennary 2,6‐sialylated **IL‐6^5^** whereas the corresponding 2,3‐sialylated **IL‐6^6^** showed a much slower clearance^[24]^ followed by the 2,6‐sialylated tetraantennary **IL‐6^8^**. Most surprisingly, the galactosylated tetraantennary **IL‐6^9^** had the slowest clearance of all the glycoforms tested whereas the corresponding galactosylated biantennary **IL‐6^2^** showed rapid clearance. Among the mannosylated glycoforms the Man2 glycoform **IL‐6^3^** showed rapid clearance (close to non‐glycosylated IL‐6) whereas the Man5 glycoform **IL‐6^4^** was cleared slowly. Furthermore, only the two glycoforms with tetraantennary N‐glycans (**IL‐6^8^** and **IL‐6^9^**) showed bimodal clearance curves.

The acute phase response of the administered variants of hIL‐6 in the rats^[23b]^ was tested at the *m*RNA and the protein level (see supporting information). *m*RNA of rat acute phase proteins was detected by qRT‐PCR for fibrinogen‐like protein 1 (FGL) > orosomucoid > C‐reactive protein. The corresponding increase of orosomucoid in plasma^[23b]^ was shown by ELISA. The biological responses appeared to be independent of the carbohydrate of the IL‐6 glycoforms.

We also tested the formation of the hexameric signaling complex^[22]^ IL‐6/IL‐6 receptor/GP‐130 by biolayer interferometry. Data analysis of the complex binding curves revealed similarly high affinities (0.2–0.4 nM) for the respective complexes containing **IL‐6** *E.coli*, **IL‐6^3^** or **IL‐6^8^**, indicating that small or large glycans on IL‐6 do not substantially interfere with the formation of the hexameric receptor complex (see supporting information).

The main proteins mediating carbohydrate‐related clearance of serum glycoproteins or neoglycoproteins from blood are two lectins in the liver, the asialoglycoprotein receptor (ASGPR) and the mannose/GlcNAc receptor (MR).^[25]^ However, their known specificities contradict the results obtained in our study. Proteins with terminal galactose on multiantennary N‐glycans should clear faster than the sialylated variants via the ASGPR (see **IL‐6^5^** vs. **IL‐6^2^** and **IL‐6^8^** vs. **IL‐6^9^**). Similarly, the MR should clear **IL‐6^4^** faster than **IL‐6^3^**.

Due to the rapid clearance of **IL‐6** from blood (*t*_1/2_≈3 min) a glycosidase‐based degradation of the N‐glycans (observed after ≈24 h)^[26]^ is unlikely to occur during the plasma lifetime of the different IL‐6 glycoforms. Thus, both the faster and the slower plasma clearance of the **IL‐6** glycoforms relative to reference **IL‐6** *E.coli* can only be a consequence of the different carbohydrate chains. We assume the following scenario: Since reference **IL‐6** *E.coli* is rapidly targeted to the liver the delayed clearance of most **IL‐6** glycoforms should be caused by lectins in the plasma, on blood cells or blood vessels. These may interact with the glycans of the **IL‐6** glycoforms temporarily and thus delay binding to the IL‐6R during the liver passages. Besides a soluble IL‐6 receptor^[27]^ (affecting all IL‐6 variants equally) blood serum of mammals also contains soluble versions of the ASGPR,^[28]^ MR,^[29]^ the group of lectins of the lectin pathway of complement activation^[30]^ and various additional soluble lectins.^[31]^ These mostly multivalent lectins should preferentially interact with **IL‐6** glycoforms bearing larger multibranched glycans and might cause a delayed targeting of lectin‐associated **IL‐6** glycoforms to the liver. The less branched smaller glycans are presumably not well bound by the serum lectins but may still be recognized by lectins in the liver leading to an accelerated overall clearance of these glycoforms. The serum concentration of the human lectins of the lectin pathway of complement activation was found to be in the range of 1–20 μg mL^−1^.^[32]^ It can be assumed that the equivalent lectins in rat^[33]^ should be present in similar serum concentrations. Thus, 8 μg of IL‐6 administered to a rat with a blood serum volume of ≈10 mL^[34]^ would lead to an initial IL‐6 concentration of ≈1 μg mL^−1^ which is in the same order of magnitude as the serum lectins mentioned above.

Remarkably, the IL‐6 glycoforms with the slowest serum clearance (**IL‐6^4^**, **IL‐6^8^**, **IL‐6^9^**) correspond to those present only in low abundance in natural IL‐6, whereas the fast‐clearing variants (**IL‐6^5^**, **IL‐6^3^**, **IL‐6^2^**) correspond to the most abundant glycoforms of natural IL‐6. The unexpected differences in the biological properties of the various IL‐6 glycoforms could only be revealed by providing sufficient amounts of a systematically varied library of synthetic IL‐6 glycoforms.

## Conclusion

In summary the chemoenzymatic semisynthesis of a systematic library of glycoforms of hIL‐6 representing the most abundant as well as the minor N‐glycans found on natural IL‐6 was accomplished. The sequential ligations followed by a two‐step refolding/purification protocol was equally applicable to all glycoforms. The resulting hIL‐6 glycoproteins were of high‐purity and were properly folded according to CD‐spectroscopy, LC‐MS, HR‐MS and X‐ray crystallography (one structure). All glycoforms were equally active in a cellular assay. The seven IL‐6 glycoforms tested in rats showed a wide range of different plasma clearance rates markedly deviating from the expected ranking. This was particularly evident for sialylated IL‐6 glycoforms relative to asialo glycoforms. In general, larger and multibranched N‐glycans led to slower plasma clearance. These findings show that the natural microheterogeneity of the rapidly liver‐targeted cytokine IL‐6 is strongly affecting its serum lifetime, presumably mediated by interaction with endogenous soluble or membrane‐bound lectins.

## Conflict of interest

The authors declare no conflict of interest.

## Supporting information

As a service to our authors and readers, this journal provides supporting information supplied by the authors. Such materials are peer reviewed and may be re‐organized for online delivery, but are not copy‐edited or typeset. Technical support issues arising from supporting information (other than missing files) should be addressed to the authors.

Supplementary
