# Supplementary material for: Natural Glycoforms of Human Interleukin 6 Show Atypical Plasma Clearance
Source: Angew Chem Int Ed Engl. 2021 May 6;60(24):13380–7. doi: 10.1002/anie.202101496 (PMC8251587; doi:10.1002/anie.202101496)
Supplement: Supplementary file 1 — Supplementary [file ANIE-60-13380-s001.pdf]

Angewandte Chemie
Eine Zeitschrift der Gesellschaft Deutscher Chemiker

Supporting Information

Natural Glycoforms of Human Interleukin 6 Show Atypical Plasma Clearance

*Andreas Reif, Kevin Lam, Sascha Weidler, Marie Lott, Irene Boos, Juliane Lokau, Christian Bretscher, Manuel Mönnich, Lukas Perkams, Marina Schmälzlein, Christopher Graf, Jan-Patrick Fischer, Carolin Lechner, Kerstin Hallstein, Stefan Becker, Michael Weyand, Clemens Steegborn, Gerhard Schultheiss, Stefan Rose-John, Christoph Garbers, and Carlo Unverzagt**

anie_202101496_sm_miscellaneous_information.pdf

Contents:

General methods

1. IL-6 (1-42) MMBA thioester **A**

2. IL-6 (49-183) $(SMe)_3$ **C**

3. Synthesis of IL-6 (43-48) hydrazide **3**

4. Synthesis of IL-6 (43-48) nonasaccharide glycopeptide **B2**

5. Synthesis of IL-6 (43-48) tetrasaccharide glycopeptide **B3**

6. Synthesis of IL-6 (43-48) Man_5 glycopeptide **B4**

7. Synthesis of IL-6 (43-48) biantennary 2,6 sialylated glycopeptide **B5**

8. Synthesis of IL-6 (43-48) (biantennary 2,3 sialylated) undeca hydrazide **B6**

9. Synthesis of IL-6 (43-48) tetraantennary nona hydrazide **B7**

10. Synthesis of IL-6 (43-48) tetraantennary trideca hydrazide **B8**

11. Synthesis of IL-6 (43-48) (tetraantennary 2,6 sialylated) heptadeca hydrazide **B9**

12. Synthesis of 1-48 hydrazides **D**

13. Synthesis of 1-48 thioesters **E**

14. Synthesis of 1-183 full length interleukin 6 **G** and refolding to the native form **H**

15. CD spectroscopy

16. Bioassay for the measurement of IL-6 activity

17. Validation of ELISA test for IL-6 glycoforms

18. Analysis of IL-6 plasma clearance in vivo

19. Quantitative Real-Time PCR (qPCR)

20. Quantification of rat acute phase proteins

21. Complexation of IL-6 glycoforms with sIL-6R

22. Biolayer interferometry (BLI)

23. Crystallization and X-ray structure determination

General methods

Solvents were dried according to standard methods. Manual SPPS steps were performed in 2-mL, 5-mL, 10-mL or 20-mL polypropylene syringes equipped with a 25 µm polyethylene filter (Multisyntech, Germany). Fmoc or Boc protected amino acids and coupling reagents were obtained from Novabiochem (Läufelfingen, Switzerland), Iris Biotech (Marktredwitz, Germany) and Sigma Aldrich (Taufkirchen, Germany). 4-(Mercaptomethyl)-benzoic acid (MMBA),[1] and GlcNAc-NH_2[2] were synthesized according to published procedures. 2-Chlorotrityl chloride resin was purchased from Novabiochem (Läufelfingen, Switzerland). ESI-TOF mass spectra were recorded on a Micromass LCT instrument coupled to an Agilent 1100 HPLC or a Waters ACQUITY UPLC H Class System with a photodiode array detector using solvent A (H_2O + 0.1 % HCOOH) and B (CH_3CN + 0.1 % HCOOH). Direct injections into the ESI-TOF-MS were carried out with IL-6 glycoforms, which were desalted on NAP-5 columns according to the manufacturer's instructions (100 µL of IL-6 glycoform solution, elution with plain water). HR-ESI mass spectra were recorded on a Thermo Q Exactive Orbitrap mass spectrometer. Preparative RP-HPLC was performed on an Äkta Basic HPLC with UV-detection at 214, 254 and 280 nm using solvent C (H_2O + 0.1 % TFA) and D (CH_3CN + 0.1 % TFA). Solid phase extraction was carried out with SepPak Classic C18 Silica cartridges, 330 mg, 55-105 µm. Flash chromatography was performed on a GRACE Reveleris® iES-flash chromatography system with ELS-detector. Thin layer chromatography was performed on coated aluminum plates (silica gel 60 GF254, Merck Darmstadt). Spots were detected by UV light or by charring with a 1:1 mixture of 2 N H_2SO_4/0.2 % resorcinol monomethyl ether in ethanol. Dialysis tubing Zellotrans V Serie (MW cutoff: 5000, flat width 40 mm) was obtained from Roth (Germany).

The degree of loading of resins was determined by Fmoc cleavage of the penultimate amino acid. Analytical TFA deprotections were performed by adding 0.5 mg of resin or 0.2 mg of protected peptide to 100 µL of TFA/Et_3SiH/H_2O (96:2:2) for 60 min. The mixture was dried in high vacuum, the residue was dissolved in MeCN/H_2O + 0.1 % HCOOH and analyzed by HPLC-MS. MMTS (S-Methyl methanethiosulfonate) was obtained from Sigma-Aldrich. The sialyltransferases were obtained from Sigma-Aldrich. Calf-intestinal alkaline phosphatase was obtained from Roche.

1. IL-6 (1-42) MMBA thioester A

IL-6 (1-42) MMBA thioester **A** was obtained recombinantly as described previously.[1]

2. IL-6 (49-183) $(SMe)_3$ C

dissolving buffer:	8 M GdmCl, 0.5 M L-arginine, 0.15 M NaCl, 0.1 M DTT, 0.05 M cysteine, pH 8
refolding buffer:	0.5 M L-arginine, 0.15 M NaCl, pH 8
Ni^{2+} IMAC binding buffer:	6 M GdmCl, 20 mM Na_2HPO_4, 20 mM imidazole, pH 7.4

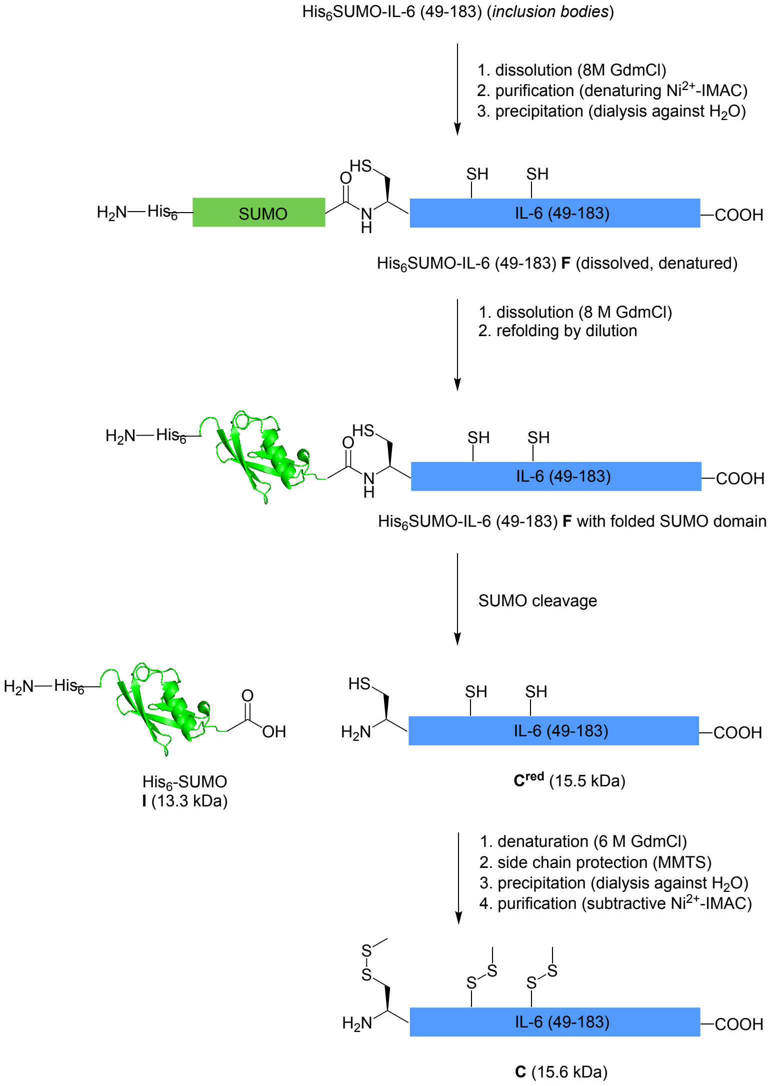

Figure S1: *Semisynthesis of disulfide-protected IL-6 (49-183)(SMe)*$_3$ ***C***

His$_6$-SUMO-IL-6 (49-183) **F** was overexpressed and purified as described previously.[1] The refolding of His$_6$-SUMO-IL-6 (49-183) **F** was performed in an anaerobic chamber. 100 mg of His$_6$-SUMO-IL-6 (49-183) **F** were dissolved in 4.5 mL of dissolving buffer, incubated for 2 h

and clarified by centrifugation (20800 g, 2 min). The supernatant was transferred into a 250 mL flask and 70 mL of refolding buffer were added under stirring (10 mL/min). The cleavage of His_6-SUMO was induced by addition of the SUMO protease SENP2 (0.8 mL of a 50 µM solution). After complete cleavage (1 d) 70 g of GdmCl (final concentration 6 M) were dissolved in the reaction mixture followed by addition of 1.2 mL (12.4 mmol) of MMTS. After incubation for one day IL-6 (49-183) $(SMe)_3$ **C** was precipitated by dialysis (2 L of H_2O, 3 times) and collected by centrifugation. The pellet was dissolved in 20 mL of Ni^{2+} IMAC binding buffer. The solution was centrifuged and the supernatant was filtered (0.45 µm, syringe filter, PVDF, Roth, Germany). The filtrate was applied to a HisPrep FF16/10 column (20 mL, GE Healthcare, Sweden) at 1 mL/min with a peristaltic pump P-1 (GE Healthcare, Sweden). Absorption was monitored at 280 nm. The column was washed with 50 mL of Ni^{2+} IMAC binding buffer and the eluate was collected in fractions of 2 mL. Fractions with A_{280} > 0.1 were pooled. The purified **C** was precipitated by dialysis (2 L of H_2O, 3 times), collected by centrifugation, washed with H_2O (1 mL; 3 times) and lyophilized.

Yield of IL-6 (49-183) $(SMe)_3$ **C**: 46 mg (2.9 µmol, 84.6 %) ESI-MS: m/z (average isotopes): $C_{684}H_{1103}N_{183}O_{212}S_{10}$ (15602.82); calculated: $(M+10H)^{10+}$ 1561.28, $(M+9H)^{9+}$ 1734.65, $(M+8H)^{8+}$ 1951.35; found: 1561.04, 1734.30, 1951.13. ESI-HRMS: m/z (exact mass): $C_{684}H_{1103}N_{183}O_{212}S_{10}$ (15592.8362); calculated: $(M+10H)^{10+}$ 1560.2909, $(M+11H)^{11+}$ 1418.5378, $(M+12H)^{12+}$ 1300.4103; found: 1560.2936, 1418.5396, 1300.4138.

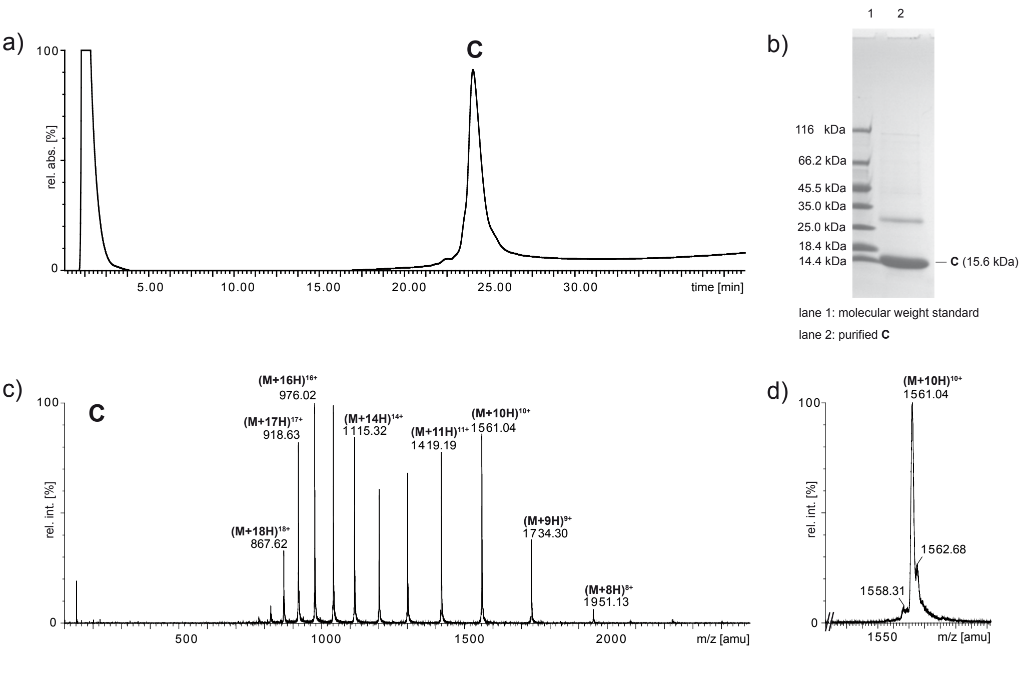

Figure S2: *Characterization of purified* ***C****: a) RP-HPLC-chromatogram; b) SDS-PAGE; c) ESI-MS analysis; d) magnified peak of ESI-MS* $(M+10H)^{10+}$.

3. Synthesis of IL-6 (43-48) hydrazide 3

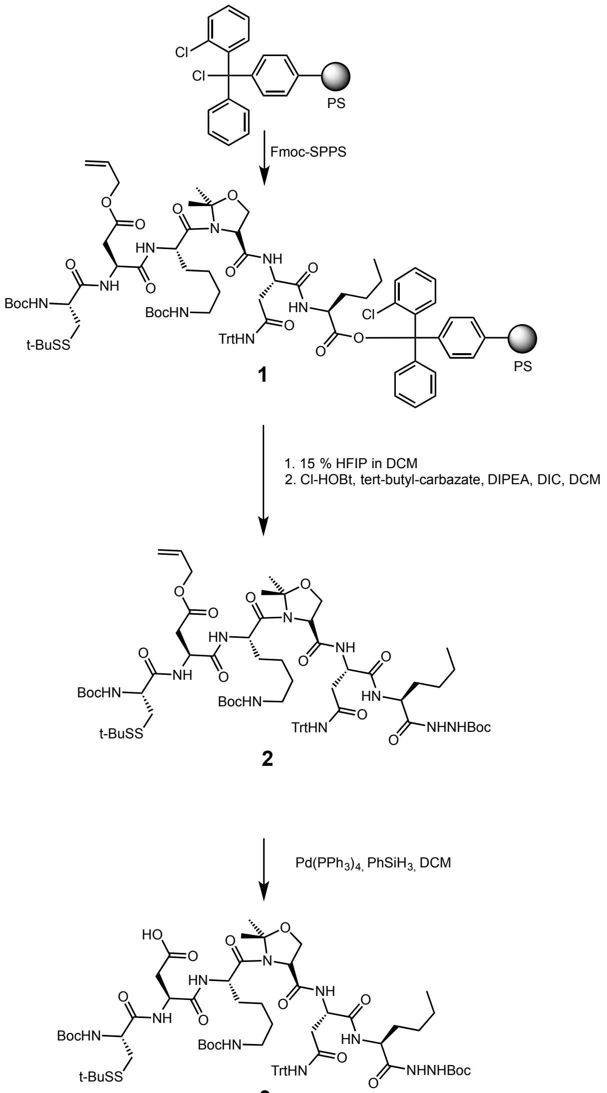

***Figure S3**: Synthesis of peptide hydrazide **3**.*

495.9 mg (793.3 μmol, loading = 1.6 mmol/g, 2.7 equiv.) of 2-chlorotrityl chloride polystyrene resin were placed in a 20-mL syringe. The resin was preswelled in CH_2Cl_2. 105.2 mg (297.3 μmol, 1 equiv.) of Fmoc-Nle-OH and 203.7 μL (1.19 mmol, 4 equiv.) of DIPEA dissolved in 2 mL of CH_2Cl_2 were added. The slurry was shaken for 15 h. The resin was washed with CH_2Cl_2 (6x) and CH_2Cl_2/methanol/DIPEA (17:2:1, 3x2 min). After washing with CH_2Cl_2 (5x) the resin was dried in high vacuum. The degree of substitution was determined (quantitative). Afterwards peptide synthesis was continued manually. The Fmoc group was removed with 20 % piperidine in DMF (5+15 min). Fmoc-Asn(Trt)-OH (5 equiv.) was coupled using HCTU and DIPEA (5 + 10 equiv.) in 7 mL of DMF for 30 min. The

couplings of Fmoc-Lys(Boc)-Ser($\Psi^{Me,Me}$pro)-OH (3 equiv.) and Fmoc-Asp(OAll)-OH (3 equiv.) were performed using PyBOP and DIPEA (3 + 6 equiv.) in 7 mL of DMF for 45 min. Washing steps were performed with DMF. Boc-Cys(StBu)-OH was coupled manually as symmetric anhydride. (The symmetric anhydride was prepared as follows: 10 equiv. amino acid were dissolved in 7 mL of CH_2Cl_2 and stirred for 10 min at 0 ° C. Then 5 equiv. of DIC were added and stirring was continued for 20 min at 0 °C. The resulting suspension was filtered and concentrated in vacuo. The residue was dissolved in 3 mL of DMF and coupling was performed for 3 h.). The resin was washed with DMF (2x) and CH_2Cl_2 (3x). The protected peptide was cleaved from the resin (3x with 15 % HFIP in CH_2Cl_2 (total 20 mL) for 15 + 5 + 5 min) and the resin was washed with CH_2Cl_2 (3x). The cleavage mixture and the washings were combined, concentrated and dried in vacuo. The residue was dissolved in 1,4-dioxane and lyophilized. The peptide (262.9 µmol, 1 equiv.), 89.2 mg (525.8 µmol, 2 eq) of Cl-HOBt, 69.5 mg (525.8 µmol, 2 equiv.) of t-butyl-carbazate and 100 µL (525.8 µmol, 2 equiv.) of DIPEA were dissolved in 3.6 mL of CH_2Cl_2 and stirred for 10 min at 0 °C. 81.9 µL (525.8 µmol, 2 equiv.) of DIC dissolved in 1.6 mL of CH_2Cl_2 were added. The reaction was stirred at 0 °C and was slowly warmed to ambient temperature. The reaction was monitored by TLC. After 19 h the solution was cooled to 0 °C and additional Cl-HOBt, tert-butyl-carbazate, DIPEA (1 equiv. each, dissolved in 1 mL of CH_2Cl_2) was added. After 5 h stirring and warming to RT the reaction was diluted with 150 mL of CH_2Cl_2 and was washed with 75 mL of H_2O (2x). The organic phase was concentrated in vacuo and dried in high vacuum. The product was purified by flash-chromatography with GRACE Reveleris® iES-Flash-System (column 40 g, cyclohexane/ acetone, 5 → 40 %, 50 min, 40 mL/min).

Yield of **2**: 270.0 mg (191.9 µmol; 64 %); ESI-MS: m/z calc. (exact mass): $C_{70}H_{102}N_{10}O_{16}S_2$ (1402.69); calculated $(M+H)^+$ 1403.69; found 1404.21.

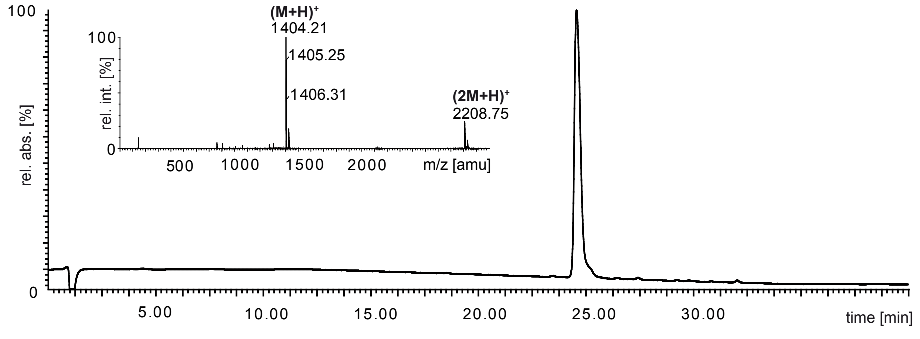

Figure S4: *HPLC-MS of purified **2**.*

270 mg (191.9 µmol, 1 equiv.) of peptide hydrazide **2** were dissolved in 2.5 mL of CH_2Cl_2 under argon. 22.2 mg (19.2 µmol, 0.1 equiv.) of $Pd(PPh_3)_4$ and 142 µL (1.2 mmol, 6 equiv.) of $PhSiH_3$ dissolved in 1.4 mL of CH_2Cl_2 were added and the mixture was stirred in the dark for 3 h. The reaction was monitored by TLC. The solution was concentrated in vacuo and the residue was dissolved in 50 % acetonitrile/H_2O (0.1 % formic acid). The sample was applied to a SepPak Classic C18 silica-column (2 g, 55-105 µm). The column was washed with 50 % acetonitrile/H_2O (0.1 % formic acid). **3** was eluted with 70 % acetonitrile/H_2O (0.1 % formic acid) and lyophilized.

Yield of **3**: 173.2 mg (126 µmol; 66 %); ESI-MS: m/z calc. (exact mass): $C_{67}H_{98}N_{10}O_{16}S_2$ (1362.66); calculated $(M+H)^+$ 1363.66; found 1364.52; ESI-HRMS: m/z (exact mass): $C_{67}H_{98}N_{10}O_{16}S_2$ (1362.6604); calculated: $(M+1H)^{1+}$ 1363.6676, $(M+2H)^{2+}$ 682.3375; found: 1363.6682, 682.3357.

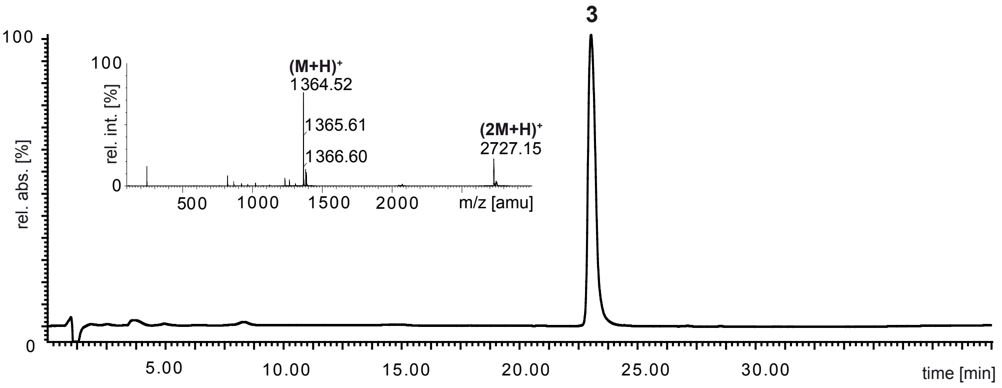

***Figure S5:** HPLC-MS of purified **3**.*

^{1}H-NMR (360 MHz, [D$_6$]-DMSO): δ = 9.72-9.56 (m, 1H, NHNHBoc), 8.70-8.55 (m, 2H, NHNHBoc, γ-NH Asn), 8.47 (d, *J* = 7.6 Hz, 1H, NH Asn), 8.35 (d, *J* = 7.7 Hz, 1H, NH Asp), 7.63 (d, *J* = 8.1 Hz, 1H, NH Nle), 7.42 (d, *J* = 6.8 Hz, 1H, NH Lys), 7.30-7.22 (m, 6H, Trt), 7.21-7.12 (m, 9H, Trt), 7.10 (d, *J* = 7.8 Hz, 1H, NH Cys), 6.74-6.62 (m, 1H, ε-NH Lys), 4.64-4.45 (m, 3H, α-CH Asp, α-CH Ser, α-CH Asn), 4.30-4.03 (m, 5H, α-CH Cys, α-CH Lys, α-CH Nle, β-CH_2 Ser), 3.10-3.00 (m, 1H, β-CHa Cys), 2.97-2.76 (m, 4H, β-CHb Cys, β-CHa Asn, ε-CH_2 Lys), 2.75-2.60 (m, 2H, β-CHa Asp, β-CHb Asn), 2.58-2.52 (m, 1H, β-CHb Asp), 1.66-1.41 (m, 10H, β-CH_2 Lys, β-CH_2 Nle, CH_3 Ψpro), 1.40-1.34 (m, 27H, CH_3 Boc), 1.31-1.14 (m, 17H, γ-CH_2 Lys, γ-CH_2 Nle, δ-CH_2 Lys, δ-CH_2 Nle, CH_3 *t*Bu), 0.87-0.78 (m, 3H, ε-CH_3 Nle),

^{13}C-NMR (91 MHz, [D$_6$]-DMSO): δ = 171.9, 171.0, 170.3, 170.1, 169.5, 169.5, 169.2 (C=O), 155.5, 155.2, 155.0 (C=O Boc), 144.7 (Cq Trt), 128.6, 127.4, 126.3 (CH Trt), 95.6 (Cq Ψpro), 79.0, 78.4, 77.3 (Cq Boc), 69.4 (Cq Trt), 67.3 (Cβ Ser), 58.9 (Cα Ser), 53.8 (Cα Cys), 51.6 (Cα Lys), 51.0 (Cα Nle), 50.0 (Cα Asn), 49.5 (Cα Asp), 47.6 (Cq *t*Bu), 42.5 (Cβ Cys), 39.5 (Cε Lys), 37.7 (Cβ Asn), 35.4 (Cβ Asp), 32.1 (Cβ Nle), 31.6 (Cβ Lys), 29.5 (CH$_3$ *t*Bu), 29.3 (Cδ Lys), 28.2, 28.1, 28.0 (CH$_3$ Boc), 26.9 (Cγ Lys, Nle), 24.9 (CH$_3$ Ψpro), 23.1 (CH$_3$ Ψpro), 21.8 (Cδ Nle), 13.8 (Cε Nle).

4. Synthesis of IL-6 (43-48) nonasaccharide glycopeptide hydrazide B2

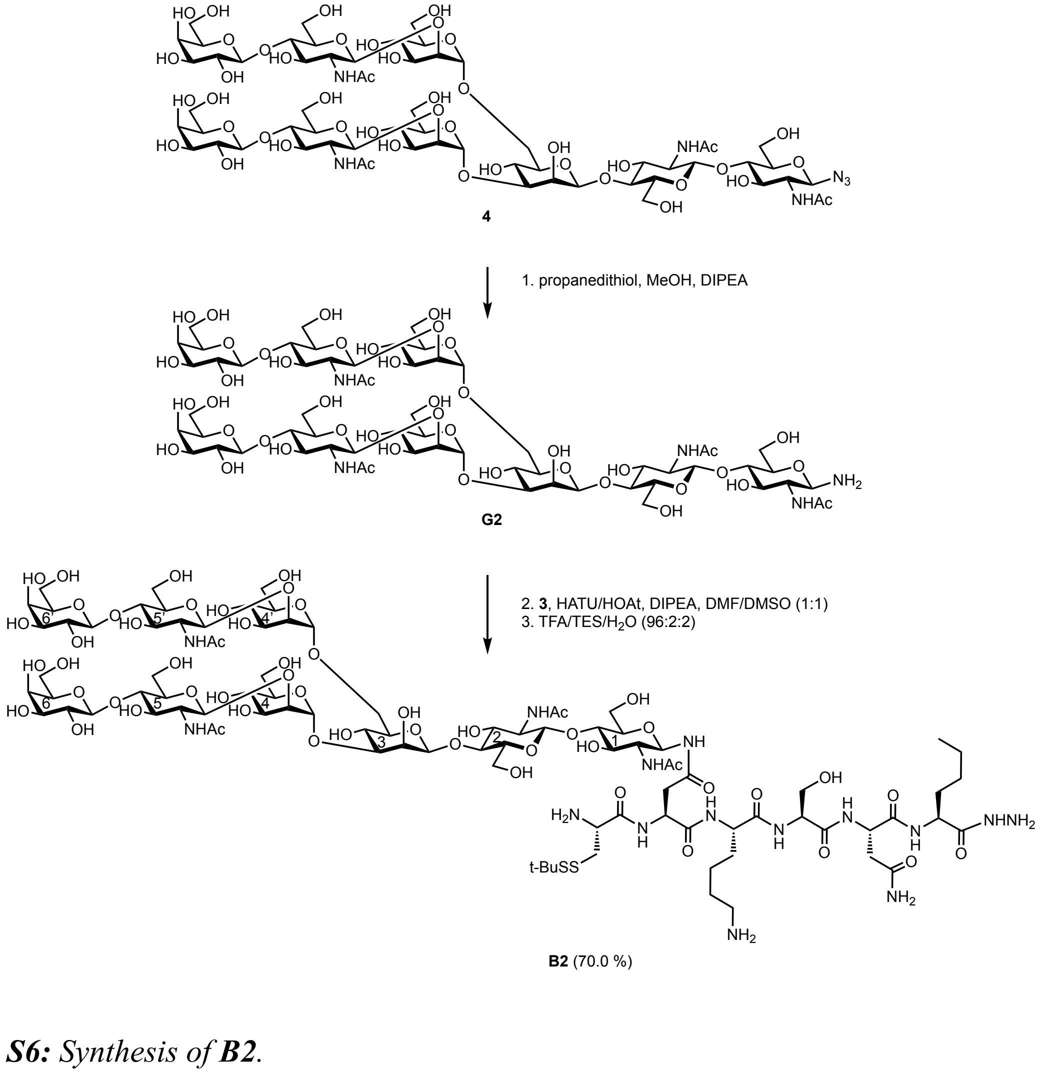

Figure S6: *Synthesis of **B2**.*

The nonasaccharide azide **4** was prepared from sialoglycopeptide (SGP) as described previously.[3] 13.4 mg (8.04 µmol, 1.0 eq) of nonasaccharide azide **4** were dissolved in 893 µL

of absolute methanol (9 mM) and 28.0 µL (160.8 µmol, 20 eq) of diisopropylethylamine and 96.9 µL (964.9 µmol, 120 eq) of 1,3-propanedithiol were added. After 2 h the resulting glycosylamine **G2** was precipitated with 1 mL of cold diethyl ether and collected by centrifugation. The pellet was washed with cold diethyl ether (2x 1 mL) and dried in high vacuum.

17.54 mg (12.87 µmol, 1.6 eq) of peptide **3**, 9.78 mg (25.73 µmol, 3.2 eq) of HATU and 3.50 mg (25.73 µmol, 3.2 eq) of HOAt were dissolved in 429.0 µL of DMF/DMSO (1:1) and 5.60 µL (32.16 µmol, 4.0 eq) of DIPEA were added. The solution was added to the glycosylamine. After 21 h the reaction was stopped by addition of 7.5 mL of TFA/TES/H_2O (95:2.5:2.5). After two hours the solution was concentrated in vacuo. The glycopeptide **B2** was precipitated by addition of 20 mL of cold diethyl ether, collected by centrifugation, washed with cold diethyl ether (2x 45 mL) and dried in high vacuum. The residue was dissolved in 20 % acetonitrile and purified by RP-HPLC (YMC-Pack C8, 250 x 20 mm, 5 µm, gradient from 5 to 25 % acetonitrile/water, 0.1 % formic acid).

Yield of **B2**: 13.50 mg (5.62 µmol, 70.0 %); ESI-MS: m/z (average isotopes): $C_{92}H_{159}N_{15}O_{54}S_2$ (2403.44); calculated $(M+H)^+$ 2404.44, $(M+2H)^{2+}$ 1202.72; found 2403.34, 1202.37.

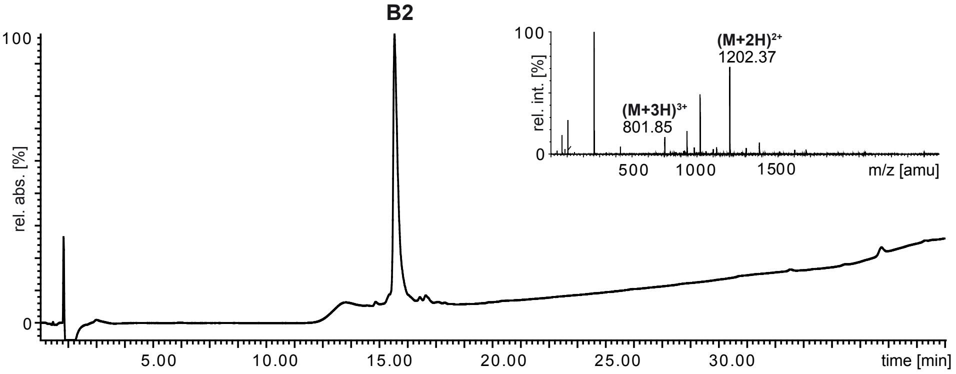

F***igure S7:*** *HPLC-MS of purified* ***B2****.*

1H-NMR (360 MHz, D_2O, 1 % [D_6]-DMSO as internal standard): d = 4.92 (d, $J_{1,2}$ < 1 Hz, 1H, H-1^4), 4.83 (d, $J_{1,2}$ = 9.5 Hz, 1H, H-1^1), 4.73 (d, $J_{1,2}$ < 1 Hz, 1H, H-$1^{4'}$), 4.65-4.52 (m, 3H, α-CH Asn-47, α -CH Asn-44, H-1^3), 4.43-4.33 (m, 3H, H-1^2, H-1^5, H-$1^{5'}$), 4.30-4.20 (m, 3H, α-CH Ser, H-1^{G1}, H-1^{G2}), 4.13-4.01 (m, 4H, α-CH Cys, α-CH Lys, α-CH Nle, H-2^3), 3.98 (dd, $J_{1,2}$ < 1 Hz, $J_{2,3}$ < 1 Hz, 1H, H-2^4), 3.90 (dd, $J_{1,2}$ < 1 Hz, $J_{2,3}$ < 1 Hz, 1H, H-$2^{4'}$), 3.82-3.67 (m, 13H, β-CHa Ser, H-3^4, H-$3^{4'}$, H-4^{G1}, H-4^{G2}, H-$6a^2$, H-$6a^3$, H-$6a^4$, H-$6a^{4'}$, H-6a,b^5, H-6a,$b^{5'}$),

3.66-3.49 (m, 24H, β-CHb Ser, H-2^{1}, H-2^{2}, H-2^{5}, H-2$^{5'}$, H-3^{1}, H-3^{2}, H-3^{3}, H-3^{5}, H-3$^{5'}$, H-4^{2}, H-4^{3}, H-4^{5}, H-4$^{5'}$, H-5^{4}, H-5^{G1}, H-5^{G2}, H-6a^{1}, H-6b^{2}, H-6b^{3}, H-6a,b^{G1}, H-6a,b^{G2}), 3.48-3.23 (m, 16H, H-2^{G1}, H-2^{G2}, H-3^{G1}, H-3^{G2}, H-4^{1}, H-4^{4}, H-4$^{4'}$, H-5^{1}, H-5^{2}, H-5^{3}, H-5$^{4'}$, H-5^{5}, H-5$^{5'}$, H-6b^{1}, H-6b^{4}, H-6b$^{4'}$), 3.12-2.99 (m, 2H, β-CH_2 Cys), 2.80 (t, J = 7.5 Hz, 2H, ε-CH_2 Lys), 2.76-2.51 (m, 4H, β-CH_2 Asn-47, β-CH_2 Asn-44), 1.88 (s, 3H, NHAc), 1.85 (s, 3H, NHAc), 1.85 (s, 3H, NHAc), 1.82 (s, 3H, NHAc), 1.75-1.44 (m, 6H, β-CH_2 Lys, β-CH_2 Nle, δ-CH_2 Lys), 1.35-1.21 (m, 2H, γ-CH_2 Lys), 1.19-1.04 (m, 13H, γ-CH_2 Nle, δ-CH_2 Nle, CH_3 tBu), 0.69 (t, J = 6.5 Hz, 3H, ε-CH_3 Nle),

13C-NMR (91 MHz, D_2O, 1 % [D_6]-DMSO as internal standard): d = 176.0, 176.0, 176.0, 175.8, 175.2, 174.0, 173.6, 173.5, 173.1, 172.9, 169.2 (C=O), 104.5 (C-1^{G1}, C-1^{G2}), 102.9 (C-1^{2}), 102.0 (C-1^{3}), 101.0 (C-1^{4}), 101.0 (C-1^{5}, C-1$^{5'}$), 98.5 (C-1$^{4'}$), 81.9 (C-3^{3}), 81.0 (C-4^{2}), 80.3 (C-4^{1}), 80.1 (C-45, C-4$^{5'}$), 79.8 (C-1^{1}), 78.0 (C-2^{4}), 77.8 (C-2$^{4'}$), 77.7 (C-5^{1}), 76.9 (C-5^{G1}, C-5^{G2}), 76.3 (C-5^{5}, C-5$^{5'}$), 76.0 (C-5^{2}), 75.1 (C-5^{3}), 74.4 (C-5^{4}), 74.2 (C-5$^{4'}$), 74.1 (C-3^{G1}, C-3^{G2}), 73.6 (C-3^{1}, C-3^{2}), 73.5 (C-3^{5}, C-3$^{5'}$), 72.5 (C-2^{G1}, C-2^{G2}), 72.5 (C-2^{3}), 71.7 (C-3^{4}, C-3$^{4'}$), 71.0 (C-4^{G1}, C-4^{G2}), 68.9 (C-4^{4}, C-4$^{4'}$), 67.4 (C-6^{3}), 67.2 (C-4^{3}), 63.3 (C-6^{4}, C-6$^{4'}$), 62.5 (C-6^{G1}, C-6^{G2}), 62.4 (Cb Ser), 61.5 (C-6^{1}, C-6^{2}, C-6^{5}, C-6$^{5'}$), 57.4 (Cα Ser), 56.4 (C-2^{2}, C-2^{5}, C-2$^{5'}$), 55.6 (Cα Lys), 55.3 (C-21), 54.3 (Cα Nle), 54.0 (Cα Cys), 51.9, 51.4 (Cα Asn-47, Cα Asn-44), 50.3 (Cq tBu), 41.6 (Cβ Cys), 40.7 (Cε Lys), 38.1, 37.6 (Cβ Asn-47, Cβ Asn-44), 32.1 (Cβ Nle), 31.8 (Cβ Lys), 30.6 (CH_3 tBu), 28.6 (Cγ Nle), 27.9 (Cδ Lys), 23.9 (NHAc), 23.8 (NHAc), 23.7 (Cγ Lys), 23.7 (NHAc), 23.1 (Cδ Nle), 14.8 (Cε Nle).

5. Synthesis of IL-6 (43-48) Man2 glycopeptide hydrazide B3

The building blocks **5** and **6** were synthesized as described.[4,5]

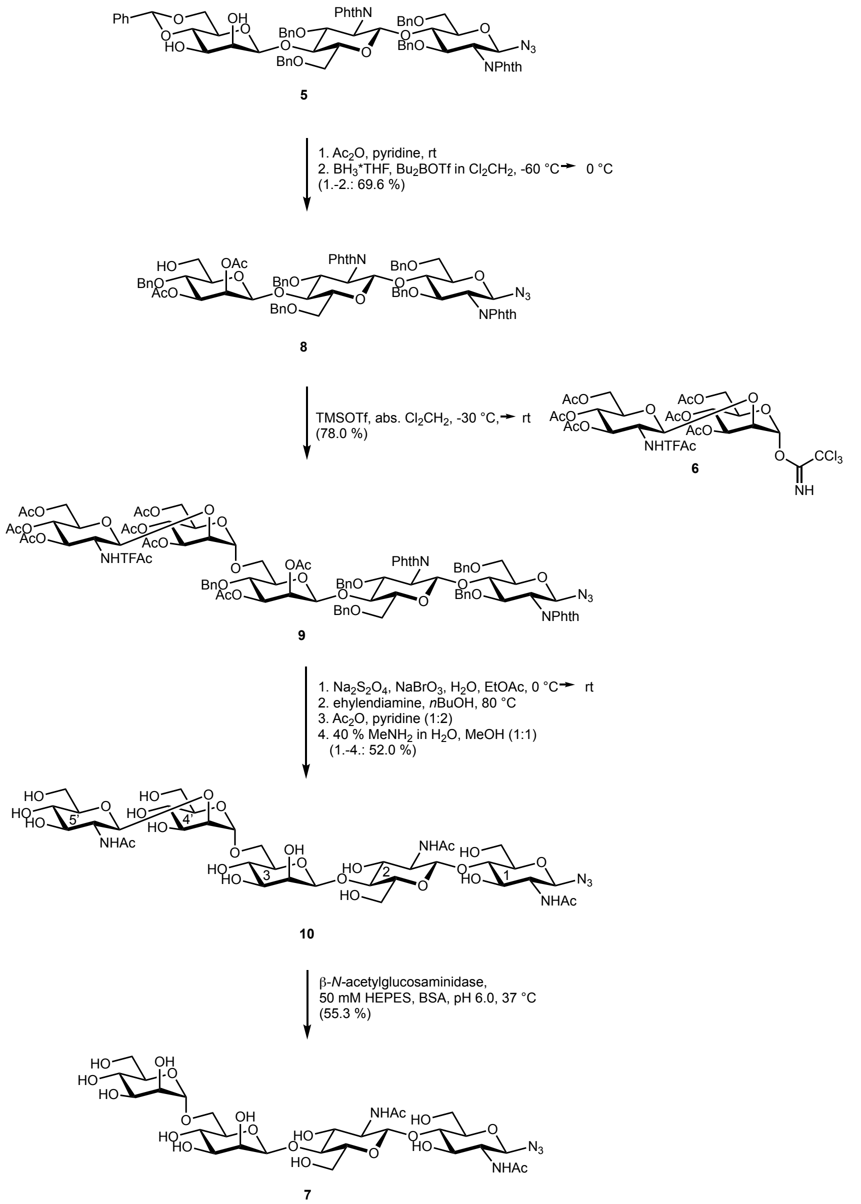

Figure S8: *Synthesis of tetrasaccharide 7.*

Synthesis of ***8***

50 mg (40.4 µmol, 1 eq) of **5** were dissolved in 1.63 mL (20.2 mmol, 500 eq) of pyridine and 809 µL (8.48 mmol, 210 eq) of acetic anhydride were added dropwise. The mixture was stirred at ambient temperature and after complete reaction (50 min, tlc: hexane/acetone, 1.5:1) the volatiles were evaporated in vacuo and residual reagents were removed by addition of toluene and azeotropic distillation (4 x). The remainder was dried in high vacuum. The acetylated trisaccharide was dissolved in 400 µl of borane tetrahydrofuran complex solution

(1 M in THF, 404 µmol, 10 eq,) under argon at -60 °C. Subsequently, 142 µL of dibutylboryl trifluoromethanesulfonate solution (1 M in CH_2Cl_2, 141.6 µmol, 3.5 eq) were added dropwise under stirring. The reaction was allowed to warm up to 0 °C over 1.5 h and stirred for another 25 min at 0 °C. After complete reaction (tlc: cyclohexane/ethyl acetate, 1:1) a mixture of 50 µL of triethylamine and 250 µL of methanol was added under stirring. The reaction was allowed to warm to ambient temperature over 1 h. The reaction was diluted with CH_2Cl_2 and extracted with 2 M $KHCO_3$, dried with $MgSO_4$, concentrated in vacuo and dried in high vacuum. **8** was purified by flash chromatography (cyclohexane/acetone, 5:1 to 3:1).

Yield: 37.2 mg **8** (28.1 µmol, 69.6 %); R_f = 0.52 (cyclohexane/ethyl acetate, 1:1); ESI-MS: m/z (exact mass): $C_{73}H_{71}N_5O_{19}$ (1321.47); calculated $(M+Na)^+$ 1344.46; found 1345.25.

*Synthesis of **9***

A suspension of 44.7 mg (33.8 µmol, 1 eq) of **8**, 35.9 mg (43.9 µmol, 1.3 eq) of **6** and 88.7 mg of freshly activated ground molecular sieves (4 Å) in 676 µL of absolute CH_2Cl_2 was stirred at ambient temperature under argon for 20 min. The suspension was cooled to -40 °C and stirred for 1 h. Subsequently, 612 µL of dilute TMSOTf (0.01 % in CH_2Cl_2, 3.4 µmol, 0.1 eq) were added over 5 min. After stirring for 1 h at -30 °C the reaction was diluted with 0.5 mL of CH_2Cl_2 and stirred for another 30 h at ambient temperature. The reaction was stopped by filtration through Celite into a stirred solution of 2 M $KHCO_3$. The mixture was extracted and the organic phase was dried with $MgSO_4$, concentrated in vacuo and dried in high vacuum. **9** was purified by flash chromatography (cyclohexane/ethyl acetate, 3:1 to 1:1).

Yield: 52.6 mg **9** (26.4 µmol, 78.0 %); R_f = 0.26 (hexane/acetone, 1.5:1); $[\alpha]_D^{27}$ = -1.61 (c = 0.55, CH_2Cl_2); ESI-MS: m/z (exact mass): $C_{99}H_{103}N_6O_{35}$ (1992.64); calculated $(M+Na)^+$ 2015.63; found 2016.54.

^{1}H-NMR (360 MHz, [D$_6$]-DMSO): δ = 9.42 (d, 1H, $J_{NH,2}$ = 9.50 Hz, NHTFAc), 7.93-7.65 (m, 8H, Pht), 7.38-7.21 (m, 15H, Ar), 6.98-6.92 (m, 2H, Ar), 6.87-6.72 (m, 8H, Ar), 5.33 (dd, 1H, H-$3^{4'}$), 5.27 (d, 1H, $J_{1,2}$ = 9.59 Hz, H-1^1), 5.20-5.10 (m, 2H, H-1^2, H-$4^{4'}$), 5.07-4.88 (m, 4H, H-2^3, H-$2^{4'}$, H-1^3, H-$3^{5'}$), 4.87-4.65 (m, 5H, H-$1^{4'}$, CH_2O, H-$4^{5'}$, CH_2O), 4.62-4.49 (m, 4H, CH_2O, CH_2O), 4.45 (d, 1H, $J_{1,2}$ = 8.52, H-$1^{5'}$), 4.37-4.32 (m, 3H, CH_2O, CH_2O), 4.19-3.91 (m, 9H, H-3^2, H-4^1, H-4^3, H-3^1, H-2^2, H-3^3, H-$6a^{5'}$, H-4^2, H-$6b^{5'}$), 3.89-3.52 (m, 11H, H- $a,b^{4'}$, H-$2^{5'}$, H-$5^{4'}$, H-2^1, H-$6a^3$, H-$6a^2$, H-$6b^3$, H-5^3, H-$6b^2$, H-5^1), 3.45-3.23 (m, 3H, H-$6a^1$, H-$6b^1$,

H-5[2]), 3.18-3.11 (m, 1H, H-5[5']) 2.11 (s, 3H, OAc), 2.03-1.83 (m, 18H, OAc), 1.75 (s,3H, OAc).

^{13}C-NMR (90 MHz, [D$_6$]-DMSO): δ = 169.9, 169.7, 169.5, 169.4, 169.1, 169.1, 167.7, 167.2 (C=O OAc), 156.5 (q, $^2J_{C,F}$ = 36.7, C=O TFAc), 138.3, 138.2, 138.1, 138.0 (C-1 Ar), 134.8, 134.6 (C-4/5 Pht), 130.7, 130.6, 130.5 (C-1/2 Pht), 128.2, 128.2, 128.0, 127.8, 127.7, 127.4, 127.4, 127.3, 127.2, 127.1, 126.8 (C Ar), 115.7 (q, $^1J_{C,F}$ = 286.7, CF_3), 99.2 (C-1[5]), 97.2 (C-1[4]), 96.7 (C-1[3]), 96.5 (C-1[2]), 84.8 (C-1[1]), 77.6 (C-4[2]), 76.4 (C-3[1]), 75.6 (C-4[3]), 75.6 (C-4[1]), 75.6 (C-5[1]) 75.1 (C-3[2]), 74.6 (C-3[3]), 74.1 (C-5[2]), 73.8, 73.7, 73.4 (CH_2O), 73.3 (C-5[3]), 73.2 (C-4[4]), 73.1 (C-5[4]), 72.2 (CH_2O), 71.6 (CH_2O), 71.5 (C-2[4]), 70.6 (C-5[5]), 69.8 (C-3[5]), 69.1 (C-3[4]), 67.9 (C-6[2]), 67.7 (C-4[5]), 67.5 (C-6[1]), 67.1 (C-6[4]), 64.4 (C-2[3]), 61.5 (C-6[5]), 61.4 (C-6[3]), 55.7 (C-2[2]), 54.5 (C-2[1]), 52.9 (C-2[5]), 20.5, 20.5, 20.4, 20.4, 20.3, 20.2, 20.2, 20.0, 20.0 (OAc).

*Synthesis of **10***

117 mg (62.4 µmol, 1 eq) of **9** were dissolved in 832 µL of ethyl acetate and cooled to 0 °C. 3.12 mL of 300 mM aqueous $NaBrO_3$ (936 µmol, 15 eq) and subsequently 7.06 mL of 130 mM aqueous $Na_2S_2O_4$ (780 µmol, 12.5 eq) were added slowly under stirring. The reaction was vigorously stirred at ambient temperature until completion (16 h, tlc: CH_2Cl_2/acetone, 2:1, R_f = 0.48) and 100 µl of 10 % aqueous $Na_2S_2O_3$ were added. The mixture was diluted with CH_2Cl_2 and extracted with H_2O. The organic phase was dried with $MgSO_4$, concentrated in vacuo and dried in high vacuum. The residue was suspended in 6.24 mL of n-butanol and 1.67 mL (25 mmol, 401 eq) of ethylenediamine were added. Subsequently, the mixture was stirred at 90 °C for 12 h. After completion (tlc: isopropyl alcohol/1 M ammonium acetate, 2:1 R_f = 0.38) the reaction was cooled to ambient temperature and the volatiles were evaporated in vacuo. Residual reagents were removed by addition of toluene and azeotropic distillation. The residue was dried in high vacuum and subsequently dissolved in a mixture of 2.77 mL of acetic anhydride and 5.55 mL of pyridine and stirred at ambient temperature until complete conversion (tlc: isopropyl alcohol/1 M ammonium acetate, 2:1 R_f = 0.88). The volatiles were evaporated in vacuo and residual reagents were removed by addition of toluene and azeotropic distillation (8 x). The dried residue was dissolved in CH_2Cl_2, extracted with 1 M HCl and 2 M $KHCO_3$, dried with $MgSO_4$, concentrated in vacuo and dried in high vacuum. The residue was dissolved in 6.24 mL of methanol and 6.24 mL of methylamine (40 % in H_2O) were added dropwise. After complete conversion (18 h, tlc: isopropyl alcohol/ 1 M ammonium acetate, 2:1) the volatiles were evaporated in vacuo and residual reagents were

removed by addition of toluene and azeotropic distillation. The dried residue was dissolved in 3.5 mL of methanol and **5e** was precipitated with 35 mL of diethyl ether and purified by size exclusion chromatography (HiLoad Superdex 30, 600x16 mm, 0.1 M NH_4CO_3, 1 mL/ min).

Yield: 39.3 mg **10** (40.3 µmol, 64.5 %); R_f = 0.68 (isopropyl alcohol/ 1 M ammonium acetate, 2:1); ESI-MS: m/z (exact mass): $C_{36}H_{60}N_6O_{25}$ (976.89); calculated $(M+H)^+$ 977.89; found 977.78.

^{1}H-NMR (360 MHz, D_2O, [D_6]-DMSO as internal standard): δ = 4.72 (d, 1H, $J_{1,2}$ < 1 Hz, H-1α$^{4'}$), 4.64-4.53 (m, 2H, H-1β^3, H-1β^1), 4.41 (d, 1H, $J_{1,2}$ = 7.64 Hz, H-1β^2), 4.35 (d, 1H, $J_{1,2}$ = 8.48 Hz, H-1β$^{5'}$), 3.92-3.86 (m, 2H, H-2$^{4'}$, H-2^3), 3.77-3.64 (m, 6H, H-6a^3, H-6a$^{5'}$, H-3$^{4'}$, H-6a^1, H-6a$^{4'}$, H-6a^2), 3.63-3.33 (m, 18H, H-6b^3, H-6b$^{5'}$, H-2^2, H-6a^2, H-2^1, H-4^3, H-4^1, H-5^2, H-2$^{4'}$, H-6a$^{4'}$, H-3^3, H-5^1, H-6b^1, H-3^1, H-5$^{4'}$, H-3^2, H-5^3, H-3$^{4'}$), 3.32-3.19 (m, 4H, H-4$^{4'}$, H-4^2, H-4$^{5'}$, H-5$^{5'}$) 1.90-1.84 (m, 9H, NAc).
^{13}C-NMR (90 MHz, D_2O, [D6]-DMSO as internal standard): δ = 176.3, 176.2, 176.2 (C=O NAc), 102.9 (C-1^{2}β, $^1J_{C-1,H-1}$ = 164.4 Hz), 102.2 (C-1^{3}β, $^1J_{C-1,H-1}$ = 162.2 Hz) 101.2 (C-1$^{5'}$α, $^1J_{C-1,H-1}$ = 163.6 Hz) 98.6 (C-1$^{4'}$α, $^1J_{C-1,H-1}$ = 171.2 Hz), 90.1 (C-1^{1}β, $^1J_{C-1,H-1}$ = 164.4 Hz), 81.1 (C-5^2), 80.4 (C-5^1), 78.1 (C-2$^{4'}$), 77.9 (C-5^3), 77.4 (C-5$^{5'}$), 76.1 (C-3^2), 76.0 (C-3$^{5'}$), 75.0 (C-5$^{4'}$), 74.5 (C-3^3), 73.9 (C-4^3), 73.6 (C-4^1), 72.0 (C-2^3), 71.5 (C-4^2), 71.5 (C-4$^{4'}$), 71.0 (C-3$^{4'}$), 70.0 (C-4$^{4'}$), 68.2 (C-3^1), 67.8 (C-6^3), 63.2 (C-6^1), 62.2 (C-6$^{4'}$), 61.6 (C-6^2), 61.4 (C-6$^{5'}$), 56.9 (C-2$^{5'}$), 56.5 (C-2^1), 56.0 (C-2^2), 23.9, 23.8, 23.7 (NAc).

Synthesis of 7

HEPES buffer: 50 mM HEPES, 0.1 mg/ml BSA, pH 6.0

21.7 mg (22.2 µmol, 1 eq) of **10** were dissolved in 1.07 mL of HEPES buffer and 10.8 µL (0.6 U) of β-*N*-acetylglucosaminidase from Jack Bean *Canavalia ensiformis* (E.C. 3.2.1.30) were added. The mixture was incubated for 19 h at 37 °C (tlc: isopropyl alcohol/ 1 M ammonium acetate, 2:1). The tetrasaccharide **7** was purified by size exclusion chromatography (HiLoad Superdex 30, 600x16 mm, 0.1 M NH_4CO_3, 0.8 mL/ min).

Yield: 9.52 mg **7** (12.3 µmol, 55.4 %); R_f = 0.72 (isopropyl alcohol/1 M ammonium acetate, 2:1); $[\alpha]_D^{27}$ = -22.1 (c = 0.3, H_2O); ESI-MS: m/z (exact mass): $C_{28}H_{47}N_5O_{20}$ (773.28); calculated $(M+H)^+$ 774.28; found 774.88.

^{1}H-NMR (360 MHz, D_2O, [D_6]-DMSO as internal standard): δ = 4.72 (d, 1H, $J_{1,2}$ < 1 Hz, H-1α$^{4'}$), 4.62-4.53 (m, 2H, H-1β^{3}, H-1β^{1}), 4.41 (d, 1H, $J_{1,2}$ = 7.75 Hz, H-1β^{2}), 3.88 (dd, 1H, $J_{1,2}$ = 2.11 Hz, $J_{2,3}$ < 1 Hz, H-2^{3}), 3.77 (dd, 1H, $J_{1,2}$ < 1 Hz, $J_{2,3}$ < 1 Hz, H-2$^{4'}$, 3.74-3.65 (m, 5H, H-6a^{1}, H-6a^{3}, H-3$^{4'}$, H-6a^{2}, H-6a$^{4'}$), 3.643-3.48 (m, 10H, H-6b^{3}, H-2^{2}, H-3^{2}, H-6b^{2}, H-2^{1}, H-6b^{1}, H-3^{1}, H-5^{2}, H-5$^{4'}$, H-6b$^{4'}$), 3.48-3.35 (m, 7H, H-5^{1}, H-3^{3}, H-4$^{4'}$, H-4^{2}, H-4^{3},H-5^{3}, H-4^{1}), 1.88 (s, 3H, NAc), 1.85 (s, 3H, NAc).

^{13}C-NMR (90 MHz, D_2O, [D_6]-DMSO as internal standard): δ = 176.5, 176.5 (C=O NAc), 103.2 (C-1^{2}β, $^{1}J_{C\text{-}1,H\text{-}1}$ = 168.1 Hz), 102.4 (C-1^{3}β, $^{1}J_{C\text{-}1,H\text{-}1}$ = 164.2 Hz) 101.5 (C-1$^{4'}$α, $^{1}J_{C\text{-}1,H\text{-}1}$ = 173.3 Hz) 90.3 (C-1^{1}β, $^{1}J_{C\text{-}1,H\text{-}1}$ = 164.9 Hz), 81.4 (C-5^{2}), 80.6 (C-5^{1}), 78.3 (C-5^{3}), 76.2 (C-4^{1}), 76.2 (C-4^{2}), 74.5 (C-3^{3}), 74.1 (C-5$^{4'}$), 73.8 (C-3^{1}), 73.4 (C-3^{2}), 72.2 (C-2^{3}), 72.2 (C-3$^{4'}$), 71.7 (C-2$^{4'}$), 68.6 (C-4^{3}), 68.6 (C-4$^{4'}$), 68.0 (C-6^{3}), 62.8 (C-6^{2}), 61.8 (C-6^{1}), 61.7(C-6$^{4'}$), 56.7 (C-2^{2}), 56.3 (C-2^{1}), 24.0, 23.9 (NAc).

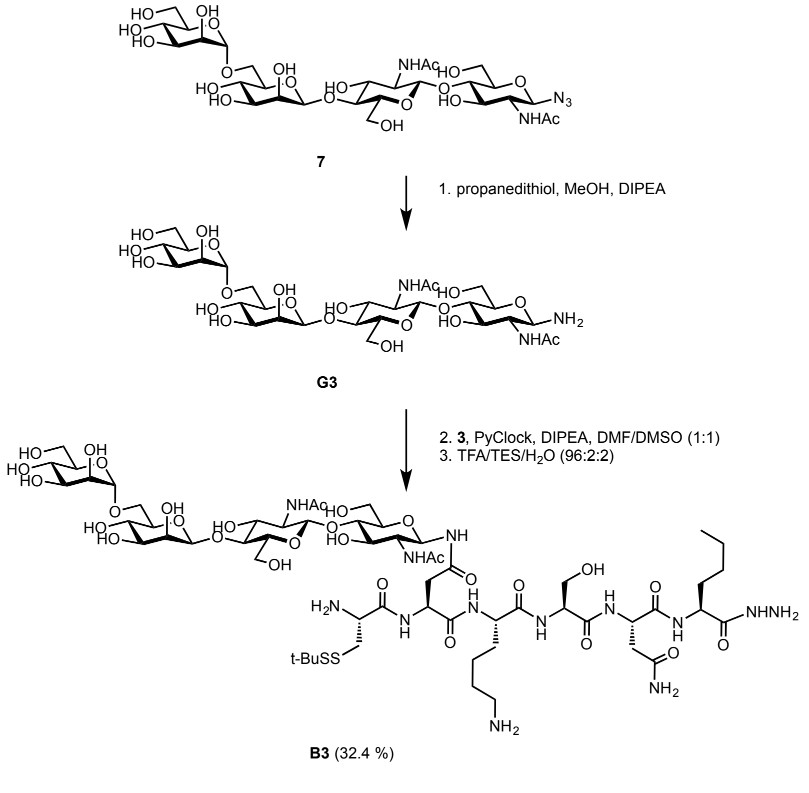

Figure S9: *Synthesis of **B3**.*

6.88 mg (8.89 µmol, 1 eq) of tetrasaccharide azide **7** were dissolved in 680 µL of absolute methanol (18 mM) and 20.9 µL (123 µmol, 14 eq) of diisopropylethylamine and 74.1 µL (738 µmol, 83 eq) of 1,3-propanedithiol were added. After 2.5 h the resulting glycosylamine **G3**

was precipitated with 1 mL of cold diethyl ether and collected by centrifugation. The pellet was washed with cold diethyl ether (2x 1 mL) and dried in high vacuum.

To a solution of 18.5 mg (13.6 µmol, 2.0 eq) of peptide **3** and 11.1 mg (20.0 µmol, 2.25 eq) of PyClock in 178 µL DMF/DMSO (1:1) 4.5 µL (26.7 µmol, 3.0 eq) of DIPEA were added. After 25 min the solution was added to the glycosylamine **G3**. After four days the reaction was stopped by addition of 0.5 mL of TFA/TES/H_2O (96:2:2). After two hours the solution was concentrated in vacuo. The glycopeptide **B3** was precipitated by addition of 1 mL of cold diethyl ether, collected by centrifugation, washed with cold diethyl ether (2x, 1 mL) and dried in high vacuum. The residue was dissolved in 5 % acetonitrile and purified by RP-HPLC (YMC Hydrosphere C18, 150 x 10 mm, 5 µm, gradient from 5 to 30 % acetonitrile/water, 0.1 % formic acid).

Yield of **B3**: 4.47 mg (2.89 µmol, 32.4 %); ESI-MS: m/z (average isotopes): $C_{58}H_{103}N_{13}O_{29}S_2$ (1510.64); calculated $(M+2H)^{2+}$ 756.32, $(M+H)^+$ 1511.64; found 756.25, 1511.50.

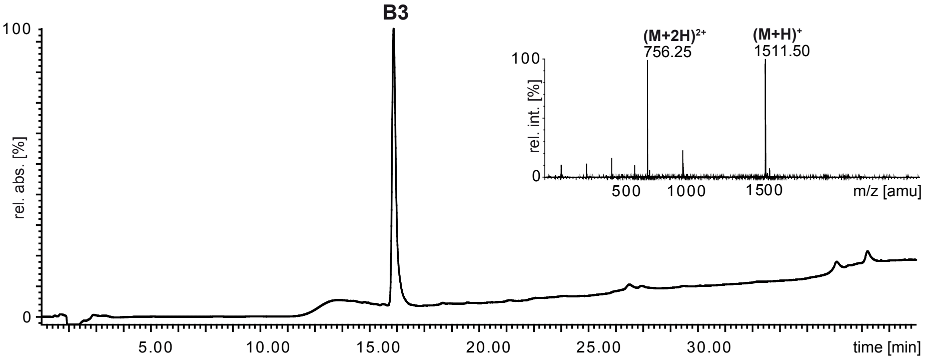

Figure S10: *HPLC-MS of purified* ***B3****.*

^{1}H-NMR (500 MHz, D_2O): δ= 4.84 (d, 1H, $J_{1,2}$ = 9.22 Hz, H-1β^1), 4.72 (d, 1H, $J_{1,2}$ < 1 Hz, H-1α$^{4'}$), 4.63-4.54 (m, 3H, α-CH Asn-47, H-1β^3, α-CH Asn-44), 4.41 (d, 1H, $J_{1,2}$ = 7.89 Hz, H-1β^2), 4.22 (t, 1H, J = 5.81 Hz, α-CH Ser), 4.12-4.06 (m, 3H, α-CH Cys, α-CH Lys, α-CH Nle), 3.88 (dd, 1H, $J_{1,2}$ = 3.02 Hz, $J_{2,3}$ < 1 Hz, 2^3), 3.76 (dd, 1H, $J_{1,2}$ = 3.33 Hz, $J_{2,3}$ = 1.58 Hz, 2$^{4'}$), 3.72-3.64 (m, 7H, 3$^{4'}$, 6a^1, 6a^2, 6a^3, β-CH_2 Ser, 2^1), 3.63-3.50 (m, 8H, 6b^3, 6b^1, 2^2, 3^2, 6a$^{4'}$, 3^1, 6b^2, 5^2), 3.48-3.39 (m, 8H, 5$^{4'}$, 5^1, 6b$^{4'}$, 4$^{4'}$, 5^3, 3^3, 4^2, 4^3), 3.36-3.32 (m, 1H, 4^1), 3.11-3.00 (m, 2H, β-CH_2 Cys), 2.80 (t, 2H, J = 6.92, ε-CH_2 Lys), 2.76-2.53 (m, 4H, β-CH_2 Asn-47, β-CH_2 Asn-44), 1.88 (s, 3H, NAc), 1.82 (s, 3H, NAc), 1.74-1.47 (m, 6H, β-CH_2 Lys, β-CH_2 Nle, δ-CH_2 Lys,), 1.33-1.21 (m, 2H, γ-CH_2 Lys), 1.18-1.06 (m, 13H, γ-CH_2 Nle, δ-CH_2 Nle, CH_3 *t*Bu), 0.69 (t, 3H, J = 6.56, ε-CH_3 Nle).

^{13}C-NMR (125 MHz, D_2O, [D6]-DMSO as internal standard): δ = 176.4, 176.0, 175.6, 174.1, 173.9, 173.5, 173.1, 169.5 (C=O), 103.0 (C-1^2), 102.2 (C-1^3), 101.3 (C-1$^{4'}$), 81.3 (C-5^2), 80.3 (C-5^1), 79.9 (C-1^1), 77.9 (C-4^1), 76.0 (C-4^2), 76.0 (C-5^3), 74.3 (C-5$^{4'}$), 74.3 (C-3^3), 74.3 (C-3^1), 73.6 (C-3^2), 72.1 (C-2^3), 72.0 (C-3$^{4'}$), 71.5 (C-2$^{4'}$), 68.5 (C-4^3), 68.5 (C-4$^{4'}$), 67.9 (C-6^3), 62.6 (C-6^2), 62.5 ($C_β$ Ser) 61.7 (C-6^1), 61.4 (C-6$^{4'}$), 57.6 ($C_α$ Ser), 56.5 (C-2^2), 55.9 ($C_α$ Lys), 55.4 (C-2^1), 54.4 ($C_α$ Nle), 54.1 ($C_α$ Cys), 52.0 ($C_α$ Asn-44), 51.5 ($C_α$ Asn-47), 50.4 (C_q *t*Bu), 41.7 ($C_β$ Cys), 40.8 ($C_ε$ Lys), 38.2, 37.7 ($C_β$ Asn-47, $C_β$ Asn-44), 32.1 ($C_β$ Nle), 31.9 ($C_β$ Lys), 30.6 (CH_3 *t*Bu), 28.8 ($C_γ$ Nle), 28.1 ($C_δ$ Lys), 23.9 ($C_γ$ Lys), 23.9, 23.7 (NAc), 23.2 ($C_δ$ Nle), 14.9 ($C_ε$ Nle).

6. Synthesis of IL-6 (43-48) Man5 glycopeptide hydrazide B4

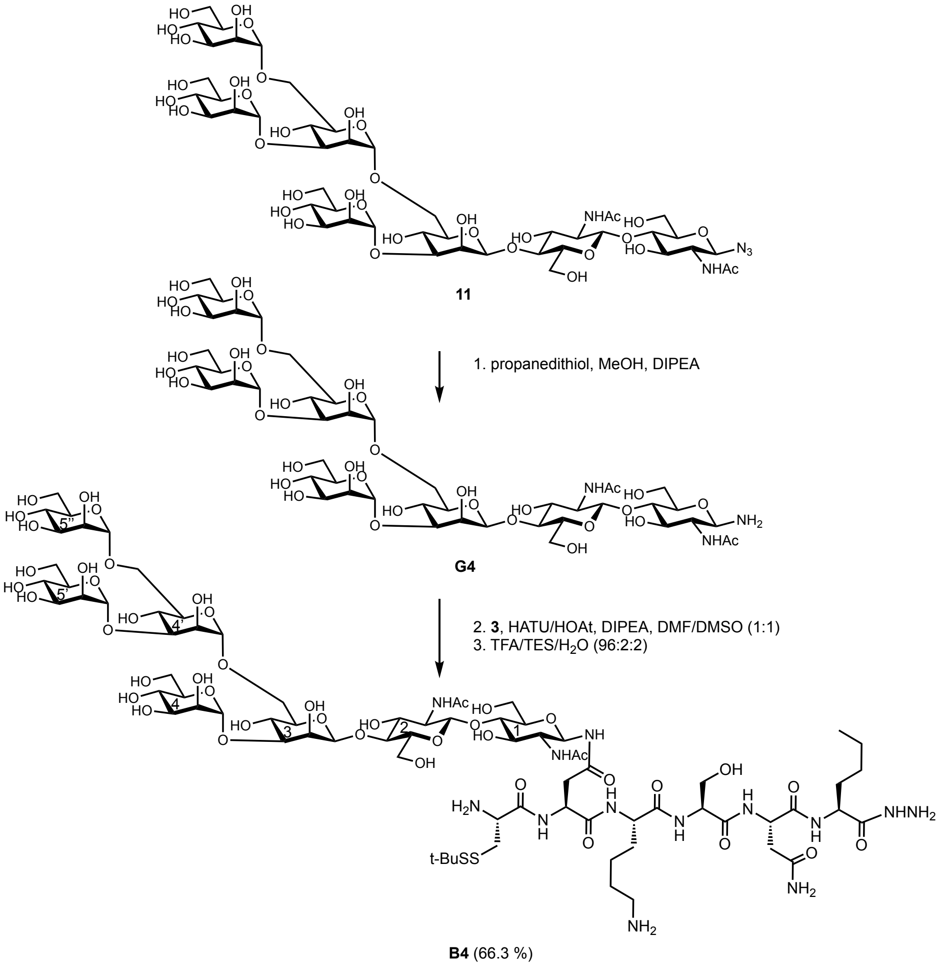

Figure S11: *Synthesis of **B4**.*

2.09 mg (1.66 µmol, 1 eq) of glycosyl azide **11**[6] were dissolved in 184 µL of absolute methanol (9 mM) and 8.5 µL (49.8 µmol, 30 eq) of diisopropylethylamine and 30.0 µL (299 µmol, 180 eq) of 1,3 propanedithiol were added. After 2.5 h the glycosylamine **G4** was precipitated with 1 mL of cold diethyl ether and collected by centrifugation. The pellet was washed with cold diethyl ether (2x 1 mL) and dried in high vacuum.

4.13 mg (3.0 µmol, 1.8 eq) of peptide **3**, 1.51 mg (3.97 µmol, 2.4 eq) of HATU and 0.51 mg (3.74 µmol, 2.3 eq) of HOAt were dissolved in 33.2 µL of DMF/DMSO (1:1) and 0.85 µL (4.98 µmol, 3.0 eq) of DIPEA were added. After 25 min the solution was added to the glycosylamine. After 21 h, 0.77 mg (5.66 µmol, 3.4 eq) of HATU were added. After a total of two days the reaction was stopped by addition of 1 mL of TFA/TES/H_2O (96:2:2). After two hours the solution was concentrated in vacuo. The glycopeptide **B4** was precipitated by addition of 2 mL cold diethyl ether, collected by centrifugation, washed with cold diethyl ether (2x, 2 mL) and dried in high vacuum. The residue was dissolved in 5 % acetonitrile and purified by RP-HPLC (YMC Hydrosphere C18, 150 x 10 mm, 5 µm, gradient from 5 to 25 % acetonitrile/water, 0.1 % formic acid)

Yield of **B4**: 2.19 mg (1.10 µmol, 66.3 %); ESI-MS: m/z (average isotopes): $C_{76}H_{133}N_{13}O_{44}S_2$ (1997.06); calculated $(M+2H)^{2+}$ 999.53, $(M+H)^+$ 1998.06; found 999.44, 1998.99; ESI-HRMS: m/z (exact mass): $C_{76}H_{133}N_{13}O_{44}S_2$ (1995.8011); calculated: $(M+H)^+$ 1996.8084, $(M+2H)^{2+}$ 998.9078, $(M+3H)^{3+}$ 666.2743; found: 1996.8034, 998.9072, 666.2739.

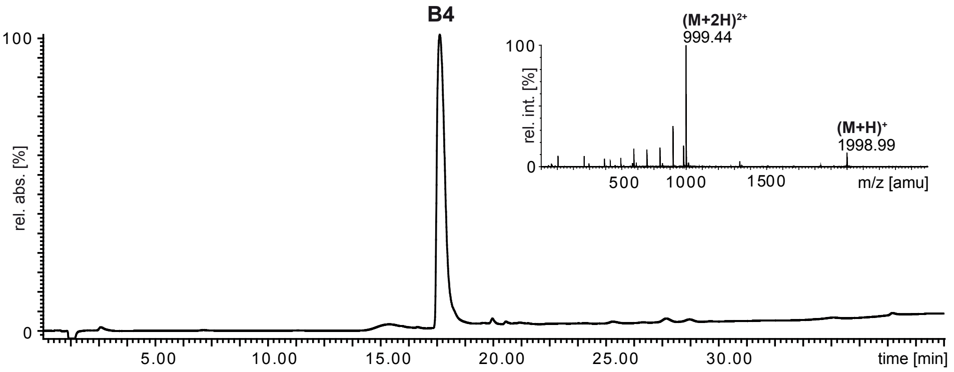

Figure S12: *HPLC-MS of purified* ***B4****.*

^{1}H-NMR (500 MHz, D_2O): δ= 4.91-4.88 (m, 2H, H-1$^{5'}$, H-1^4), 4.84 (d, 1H, $J_{1,2}$ = 9.78 Hz, H-1β^1), 4.73-4.58 (m, 4H, H-1$^{5''}$, H-1$^{4'}$, α-CH Asn-47, H-1^3), 4.56-4.53 (t, 1H, J = 6.71 Hz, α-CH Asn-44), 4.40 (d, 1H, $J_{1,2}$ = 7.16 Hz, H-1β^2), 4.24 (t, 1H, J = 5.94 Hz, α-CH Ser), 4.13-4.01 (m, 4H, α-CH Cys, H-2^3, α-CH Lys, α-CH Nle), 3.96 (dd, 1H, $J_{1,2}$ < 1 Hz, $J_{2,3}$ < 1 Hz,

$2^{4'}$), 3.89-3.86 (m, 2H, $2^{5'}$, 2^{4}), 3.83-3.78 (m, 2H, $2^{5''}$, $6a^{3}$), 3.76-3.63 (m, 15H, $6a^{4'}$, $6a^{2}$, $3^{4'}$, $6a^{1}$, $3^{5'}$, 3^{4}, $3^{5''}$, 3^{2}, $6a^{5''}$, $6a^{5'}$, $6a^{4}$, β-CH_2 Ser, 3^{1}, 2^{1}), 3.63-3.51 (m, 14H, $4^{4'}$, 2^{2}, $6b^{4'}$, 3^{3}, 4^{1}, $4^{5'}$, 4^{4}, 5^{2}, $6b^{2}$, $6b^{1}$, $6b^{5'}$, $6b^{5''}$, $6b^{4}$, 4^{2}), 3.51-3.33 (m, 9H, $6b^{3}$, 4^{3}, $5^{5'}$, 5^{4}, $5^{4'}$, 5^{1}, $4^{5''}$, 5^{3}, $5^{5''}$), 3.08-2.98 (m, 2H, β-CH_2 Cys), 2.81 (t, 2H, J = 6.29, ε-CH_2 Lys), 2.76-2.54 (m, 4H, β-CH_2 Asn-47, β-CH_2 Asn-44), 1.87 (s, 3H, NAc), 1.83 (s, 3H, NAc), 1.75-1.46 (m, 6H, β-CH_2 Lys, β-CH_2 Nle, δ-CH_2 Lys,), 1.34-1.23 (m, 2H, γ-CH_2 Lys), 1.18-1.07 (m, 13H, γ-CH_2 Nle, δ-CH_2 Nle, CH_3 *t*Bu), 0.69 (t, 3H, J = 6.56, ε-CH_3 Nle).

^{13}C-NMR (125 MHz, D_2O): δ= 104.4 (C-1^{4}), 104.2 (C-$1^{5'}$), 103.1 (C-1^{2}), 102.2 (C-1^{3}), 101.8 (C-$1^{4'}$), 101.1 (C-$1^{5''}$), 82.4 (C-5^{2}), 80.4 (C-$3^{4'}$), 80.2 (C-5^{1}), 80.0 (C-1^{1}), 77.9 (C-$5^{5''}$), 76.4 (C-5^{3}), 76.2 (C-5^{1}), 75.2 (C-$4^{4'}$), 75.0 (C-3^{1}), 74.6 (C-$5^{5'}$), 74.6 (C-5^{4}), 74.5 (C-$4^{5'}$), 74.5 (C-4^{4}), 73.8 (C-4^{1}), 72.5 (C-$3^{5''}$), 72.3 (C-$3^{5'}$), 72.2 (C-3^{4}), 72.0 (C-2^{3}), 72.0 (C-$2^{5'}$), 71.8 (C-2^{4}), 71.7 (C-$2^{5''}$), 71.3 (C-$2^{4'}$), 68.6 (C-$4^{5''}$), 68.5 (C-$4^{4'}$), 67.4 (C-3^{2}), 67.2 (C-3^{3}), 66.8 (C-6^{3}), 63.0 (C-$6^{4'}$), 63.0 (C-6^{2}), 62.7 (C-6^{1}), 62.7 (C-$6^{5''}$), 62.7 ($C_β$ Ser) 61.6 (C-$6^{5'}$), 61.6 (C-6^{4}), 57.6 ($C_α$ Ser), 56.6 (C-2^{2}), 56.0 ($C_α$ Lys), 55.5 (C-2^{1}), 54.7 ($C_α$ Nle), 54.6 ($C_α$ Cys), 52.1 ($C_α$ Asn-44), 51.7 ($C_α$ Asn-47), 40.8 ($C_ε$ Lys), 37.9, 37.7 ($C_β$ Asn-47, $C_β$ Asn-44), 32.1 ($C_β$ Nle), 32.0 ($C_β$ Lys), 30.7 (CH_3 *t*Bu), 28.8 ($C_γ$ Nle), 28.2 ($C_δ$ Lys), 24.0 ($C_γ$ Lys), 23.9, 23.8 (NAc), 23.3 ($C_δ$ Nle), 14.9 ($C_ε$ Nle).

7. Synthesis of IL-6 (43-48) biantennary 2,6-sialylated glycopeptide hydrazide B5

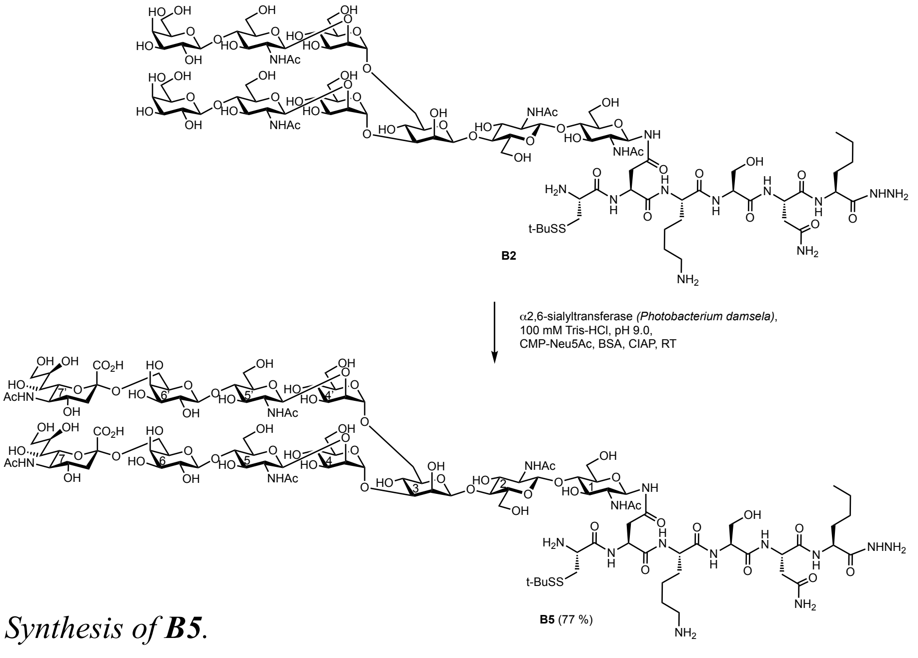

Figure S13: *Synthesis of* ***B5****.*

Glycopeptide **B5** was prepared by enzymatic elongation of **B2**. 1.0 mg of **B2** and 1.23 mg (1.87 μmol, 4.5 eq) of CMP-*N*-acetyl-β-neuraminic acid were dissolved in 41.6 μL of Tris-

buffer (100 mM Tris-HCl, 1 mg/ mL BSA, 20 μM NaN_3, pH 8.5). 0.8 μL of calf-intestinal alkaline phosphatase (2 U) and 10.0 μL of alpha-2,6-sialyltransferase from *Photobacterium damsela* (100 mU) were added. After a total reaction time of 19 h, the reaction mixture was purified by RP-HPLC (Ascentis C18, 150 x 10 mm, 5 μm, gradient from 5 to 20 % acetonitrile/water, 0.1 % formic acid). The eluate was neutralized by addition of 1 mM Na_2CO_3 to pH 7-8 and lyophilized. The residue was dissolved in 0.1 mL of H_2O and passed over a Sephadex G-25 column (164 x 15 mm, 10 % acetonitrile/water, flow rate 1.0 mL/min).

Yield of **B5**: 0.95 mg (0.32 μmol, 77 %); ESI-MS: m/z (average isotopes): $C_{114}H_{193}N_{17}O_{70}S_2$ (2985.95); calculated $(M+3H)^{3+}$ 996.93, $(M+2H)^{2+}$ 1493.98; found 996.47, 1494.33; ESI-HRMS: m/z (exact mass): $C_{114}H_{193}N_{17}O_{70}S_2$ (2984.1507); calculated: $(M+2H)^{2+}$ 1493.0826, $(M+3H)^{3+}$ 995.7242; found: 1493.0825, 995.7241.

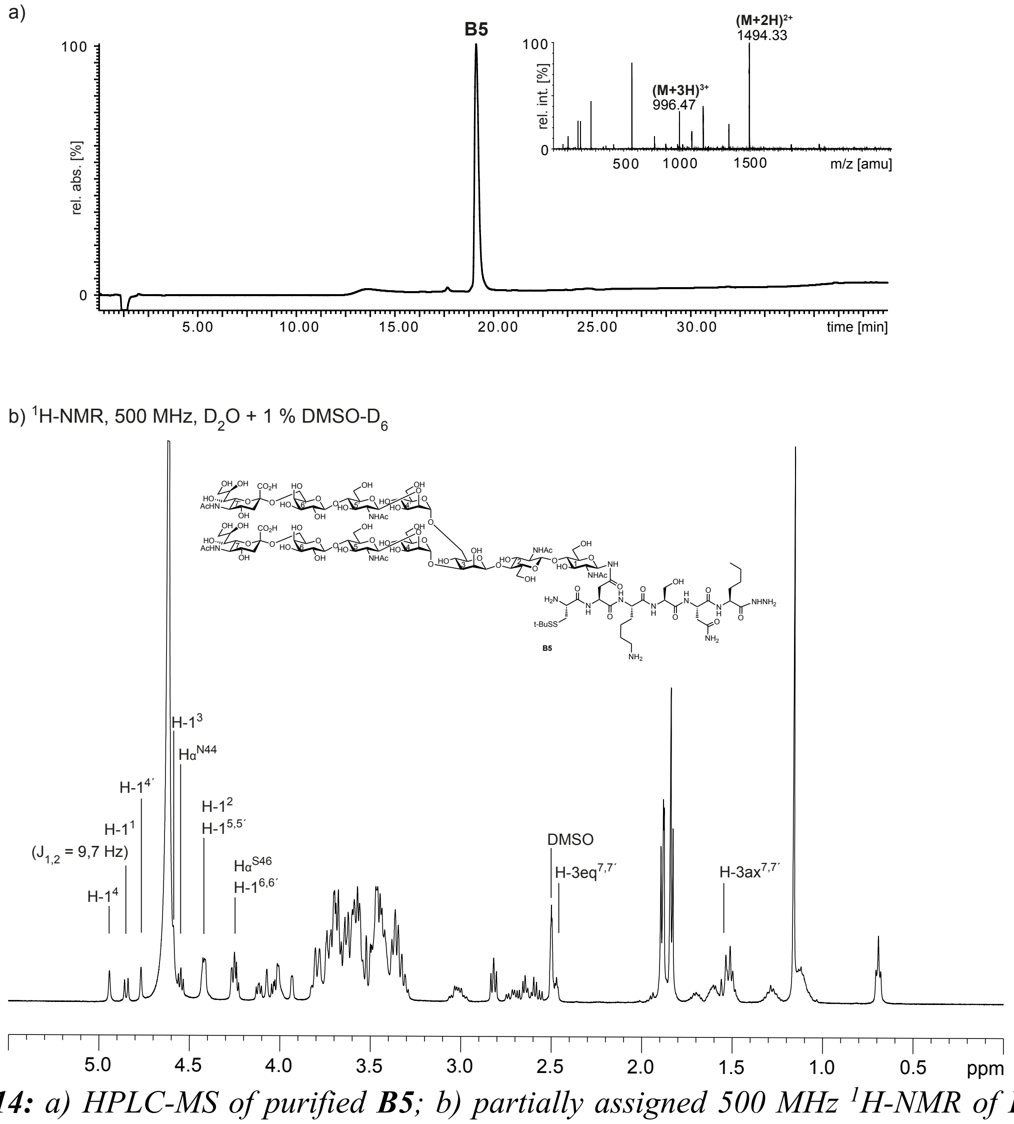

***Figure S14:** a) HPLC-MS of purified **B5**; b) partially assigned 500 MHz ^{1}H-NMR of **B5** in D_2O ($DMSO$-D_6 as internal reference.*

8. Synthesis of IL-6 (43-48) biantennary 2,3-sialylated glycopeptide hydrazide B6

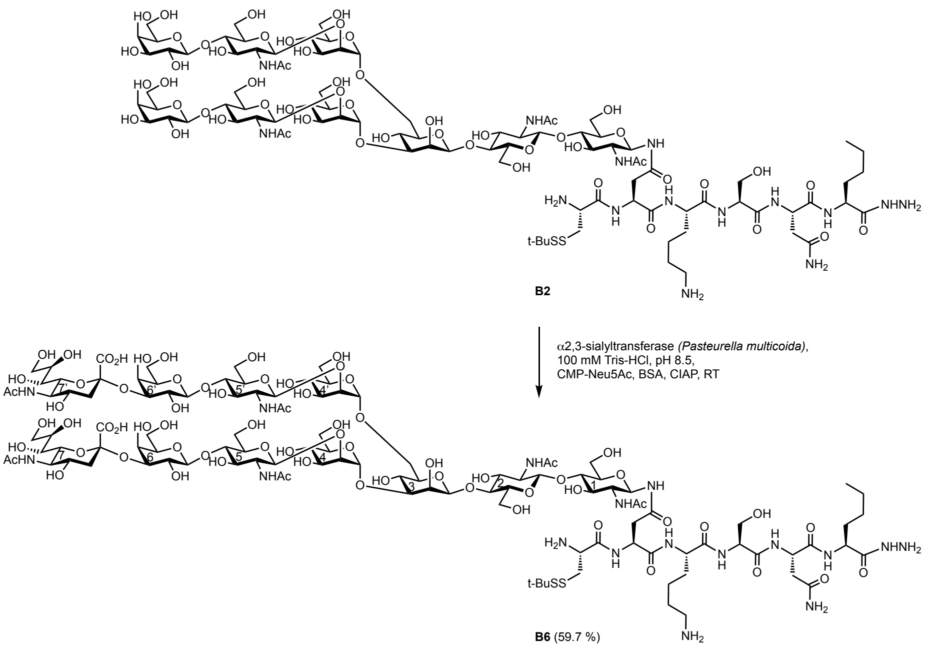

Figure S15: *Synthesis of* ***B6****.*

B6 was prepared by enzymatic elongation of **B2**. 1.50 mg (0.62 µmol, 1.0 eq, 6 mM) of **B2** and 2.52 mg (3.96 µmol, 6.3 eq) of CMP-*N*-acetyl-neuraminic acid were dissolved in 99.5 µL of Tris-buffer (100 mM Tris-HCl, 1 mg/ mL BSA, pH 8.5). Calf-intestinal alkaline phosphatase (2.0 µL, 1000U/mL and alpha-2,3-sialyltransferase from *Pasteurella multocida* (30 mU in 3.0 µL) were added. After 1 h **B6** was purified by RP-HPLC (YMC Hydrosphere C18, 150 x 10 mm, 5 µm, gradient from 0 to 40 % acetonitrile/water, 0.1 % formic acid).

Yield of **B6**: 1.10 mg (0.37 µmol, 59.0 %); ESI-MS: m/z (average isotopes): $C_{114}H_{193}N_{17}O_{70}S_2$ (2985.95); calculated $(M+3H)^{3+}$ 996.32, $(M+2H)^{2+}$ 1493.98; found 996.65, 1494.42

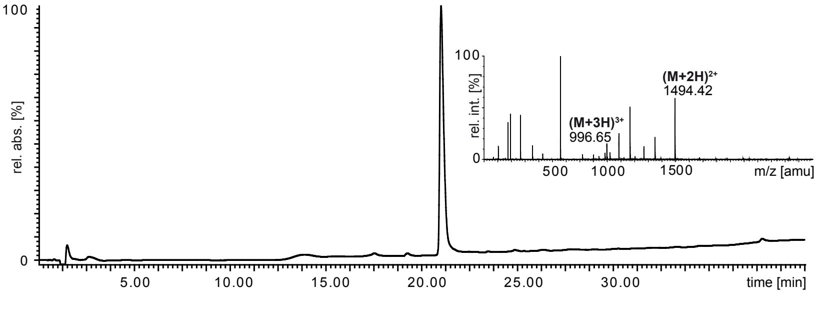

Figure S16: *HPLC-MS of purified* ***B6****.*

^{1}H-NMR (500 MHz, D_2O): δ= 4.92 (d, 1H, $J_{1,2}$ < 1 Hz, H-1α^{4}), 4.84 (d, 1H, $J_{1,2}$ = 9.61 Hz, H-1β^{1}), 4.73 (d, 1H, $J_{1,2}$ < 1 Hz, H-1α$^{4'}$), 4.63-4.52 (m, 3H, α-CH Asn-47, H-1β^{3}, α-CH Asn-44), 4.41 (d, 1H, $J_{1,2}$ = 7.78 Hz, H-1β^{2}), 4.39-4.33 (m, 4H, H-1β^{5}, H-1β$^{5'}$, H-1β^{6}, H-1β$^{6'}$), 4.23 (t, 1H, *J* = 5.49 Hz, α-CH Ser), 4.13-3.98 (m, 5H, α-CH Cys, α-CH Lys, 2^{3},α-CH Nle, 2^{4}), 3.94-3.90 (m, 3H, H-3^{6}, H-2$^{4'}$, H-3$^{6'}$), 3.81-3.73 (m, 7H, H-6a^{6}, H-6a$^{6'}$, H-6^{7}, H-6$^{7'}$, H-3^{2}, H-4^{6}, H-4$^{6'}$), 3.72-3.64 (m, 14H, H-9a^{7}, H-9a$^{7'}$, H-3^{4}, H-3^{1}, H-6a$^{4'}$, H-3$^{4'}$, H-3^{5}, H-3$^{5'}$, β-CH_2 Ser, H-6b^{6}, H-6b$^{6'}$, H-6a^{4}, H-2^{1}), 3.63-3.48 (m, 24H, H-5^{7}, H-5$^{7'}$, H-6a^{2}, H-2^{2}, H-3^{3}, H-6b^{2}, H-5^{2}, H-6a^{5}, H-6a$^{5'}$, H-2^{5}, H-4^{1}, H-2$^{5'}$, H-5^{1}, H-6a^{1}, H-5^{5}, H-5$^{5'}$, H-6a,b^{3}, H-6b^{5}, H-6b$^{5'}$, H-5^{3}, H-4^{3}, H-4^{7}, H-4$^{7'}$), 3.47-3.26 (m, 20H, H-4^{5}, H-4$^{5'}$, H-6b$^{4'}$, H-8^{7}, H-8$^{7'}$, H-6b^{1}, H-6b^{4}, H-5$^{4'}$, H-5^{4}, H-9b^{7}, H-9b$^{7'}$, H-5^{6}, H-4^{2}, H-2^{6}, H-2$^{6'}$, H-5$^{6'}$, H-7^{7}, H-7$^{7'}$, H-4$^{4'}$, H-4^{4}), 3.11-3.00 (m, 2H, β-CH_2 Cys), 2.80 (t, 2H, *J* = 7.32, ε-CH_2 Lys), 2.74-2.53 (m, 6H, β-CH_2 Asn-47, β-CH_2 Asn-44, H-3eq^{7}, H-3eq$^{7'}$), 1.90-1.81 (m, 18, NAc), 1.73-1.45 (m, 8H, β-CH_2 Lys, β-CH_2 Nle, H-3ax^{7}, H-3ax$^{7'}$, δ-CH_2 Lys), 1.33-1.22 (m, 2H, γ-CH_2 Lys), 1.18-1.06 (m, 13H, γ-CH_2 Nle, δ-CH_2 Nle, CH_3 *t*Bu), 0.68 (t, 3H, *J* = 6.56, ε-CH_3 Nle).

^{13}C-NMR (125 MHz, D_2O, [D6]-DMSO as internal standard): δ = 104.1 (C-1^{6}), 104.1 (C-1$^{6'}$), 102.8 (C-1^{2}), 101.8 (C-1^{3}), 101.1 (C-1^{4}), 101.0 (C-1^{5}), 101.0 (C-1$^{5'}$), 98.6 (C-1$^{4'}$), 80.6 (C-5^{2}), 80.2 (C-8^{7}), 80.2 (C-8$^{7'}$), 79.8 (C-1^{1}), 79.7 (C-5^{5}), 79.7 (C-5$^{5'}$), 77.9 (C-2^{4}), 77.7 (C-2$^{4'}$), 77.5 (C-7^{7}), 77.5 (C-7$^{7'}$), 77.0 (C-3^{6}), 77.0 (C-3$^{6'}$), 76.7 (C-5^{3}), 76.3 (C-5^{6}), 75.9 (C-5$^{6'}$), 75.0 (C-5^{1}), 74.4 (C-5$^{4'}$), 74.4 (C-5^{4}), 74.1 (C-3^{3}), 73.6 (C-4^{7}), 73.6 (C-4$^{7'}$), 73.5 (C-4^{1}), 73.2 (C-3$^{4'}$), 73.2 (C-3^{4}), 71.6 (C-2^{3}), 70.9 (C-3^{1}), 70.9 (C-3^{5}), 70.9 (C-3$^{5'}$), 70.8 (C-2^{6}), 70.8 (C-2$^{6'}$), 69.9 (C-4^{3}), 69.8 (C-4^{5}), 69.8 (C-4$^{5'}$), 69.5 (C-3^{2}), 69.0 (C-6^{7}), 69.0 (C-6$^{7'}$), 68.7 (C-4^{4}), 68.7 (C-4$^{4'}$), 64.0 (C-6^{4}), 64.0 (C-6$^{4'}$), 63.2 (C-6^{7}), 63.2 (C-6$^{7'}$), 62.5 ($C_β$ Ser) 62.5 (C-6^{3}), 62.5 (C-6^{5}), 62.5 (C-6$^{5'}$), 61.4 (C-6^{6}), 61.4 (C-6$^{6'}$), 61.3 (C-6^{3}), 61.3 (C-6^{1}), 57.3 ($C_α$ Ser), 56.3 (C-2^{2}), 56.3 (C-2^{5}), 56.3 (C-2$^{5'}$), 55.5 ($C_α$ Lys), 55.0 (C-5^{7}), 55.0 (C-5$^{7'}$), 54.4 ($C_α$ Nle), 54.1 ($C_α$ Cys), 53.2 (C-2^{1}), 51.8 ($C_α$ Asn-44), 51.4 ($C_α$ Asn-47), 41.5 ($C_β$ Cys), 41.1 (C-3^{7}), 41.1 (C-3$^{7'}$), 40.6 ($C_ε$ Lys), 37.7 ($C_β$ Asn-47, $C_β$ Asn-44), 32.0 ($C_β$ Nle), 31.9 ($C_β$ Lys), 30.3 (CH_3 *t*Bu), 28.5 ($C_γ$ Nle), 27.8 ($C_δ$ Lys), 23.7 ($C_γ$ Lys), 23.7 (NAc), 23.0 ($C_δ$ Nle), 14.6 ($C_ε$ Nle).

9. Synthesis of IL-6 (43-48) tetraantennary glycopeptide hydrazide B7

The tetrantennary nonasaccharide **12** was obtained as described.[5, 7]

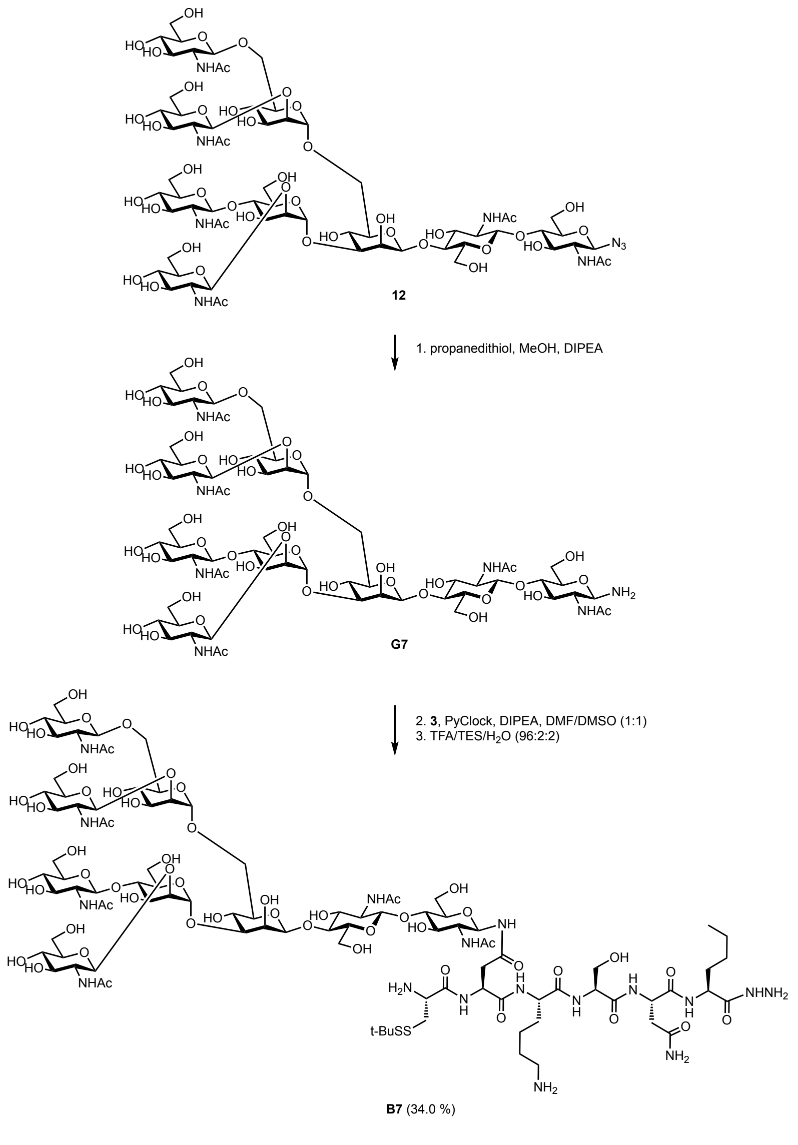

Figure S17: *Synthesis of* ***B7****.*

6.4 mg (3.66 µmol, 1 eq) of glycosyl azide **12** were dissolved in absolute methanol (200 µL) and 6.25 µL (36.5 µmol, 10 eq) of DIPEA and 22 µL (219 µmol, 60 eq) of 1,3-propanedithiol were added. After 1.5 h 100 µl of absolute methanol were added. After a total reaction time of 3.5 h the solution was concentrated in vacuo. The residue was dissolved in 720 µl of absolute methanol and the glycosylamine **G7** was precipitated with 2.2 mL of cold diethyl ether and centrifuged. This procedure was repeated two more times. The pellet was dried in high vacuum.

7.5 mg (5.50 µmol, 1.65 eq) of peptide **3** and 4.56 mg (8.2 µmol, 2.5 eq) of PyClock were dissolved in 74 µL of DMF/DMSO (1:1) and 1.4 µL (8.2 µmol, 2.5 eq) of DIPEA were added. After 10 min the solution was added to the 5.75 mg of the glycosylamine **G7**. After 34 h the reaction was stopped by addition of 1 mL of TFA/TES/H_2O (96:2:2). After 1.5 h the solution was concentrated in vacuo. The glycopeptide **B7** was precipitated by addition of 5 mL of cold diethyl ether, collected by centrifugation, washed with cold diethyl ether (2x, 5 mL) and dried in high vacuum. The residue was dissolved in 5 % acetonitrile/water and purified by RP-HPLC (Supelco Ascentis C18, 250 x 10 mm, 5 µm, gradient from 5 to 20 % acetonitrile/water, 0.1 % TFA)

Yield of **B7**: 2.82 mg (1.13 µmol, 34.0 %); ESI-MS: m/z (average isotopes): $C_{96}H_{165}N_{17}O_{54}S_2$ (2485.55); calculated $(M+2H)^{2+}$ 1243.78; found 1244.06.

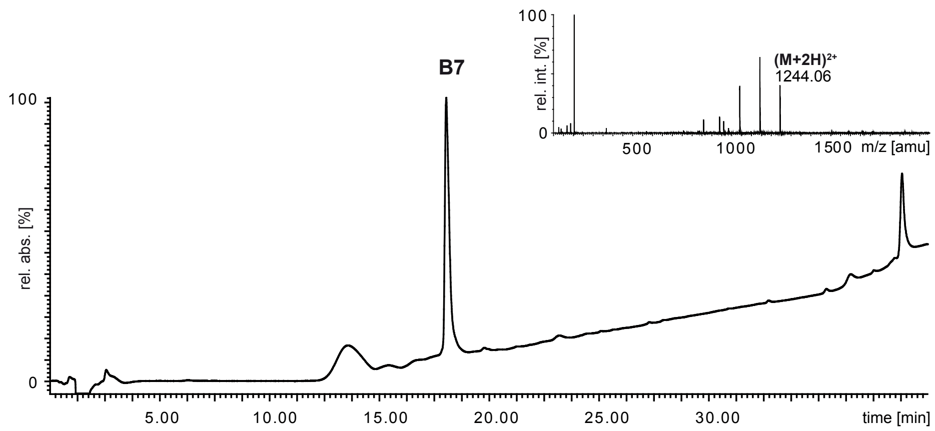

Figure S18: *HPLC-MS of purified* ***B7****.*

10. Synthesis of IL-6 (43-48) tetraantennary galactosylated glycopeptide B8

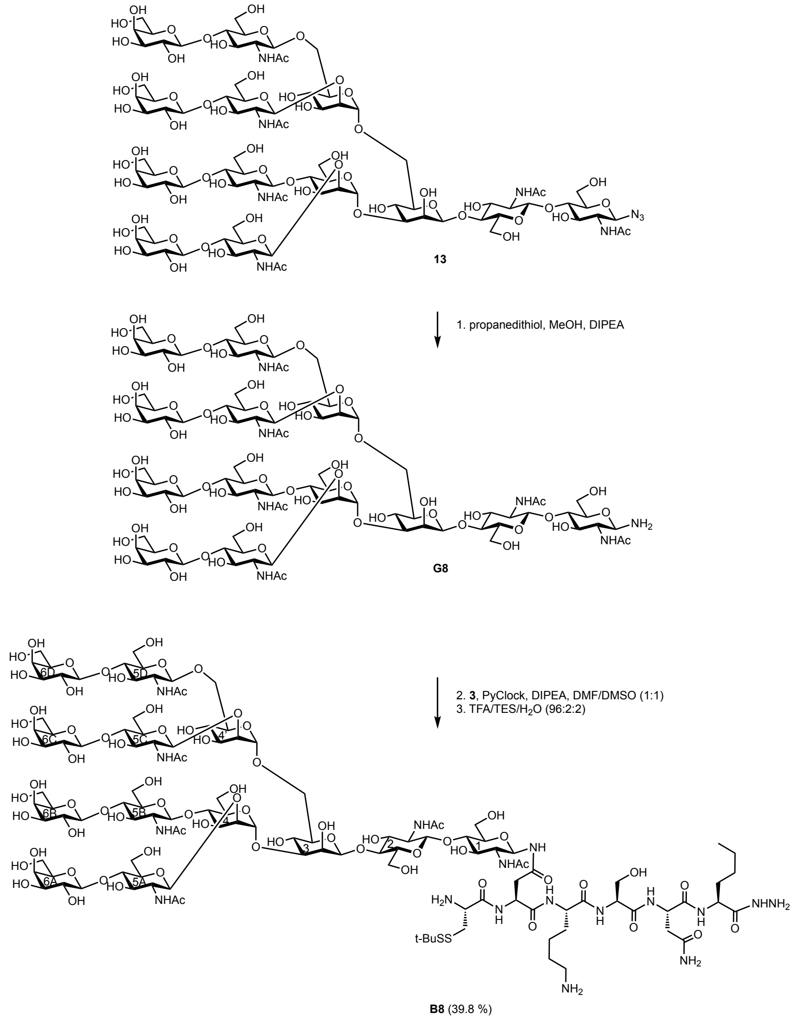

Figure S19: *Synthesis of* ***B8****.*

4.0 mg (1.67 µmol, 1 eq) of glycosyl azide **13**[7] were dissolved in 186 µL of absolute methanol and 8.6 µL (50.2 µmol, 30 eq) of DIPEA and 30.4 µL (30.8 µmol, 180 eq) of 1,3-propanedithiol were added. After 5 h the resulting glycosylamine **G8** was precipitated with 1 mL of cold diethyl ether and collected by centrifugation. The residue was dissolved in 300 µL methanol and precipitated with 1 mL of cold diethyl ether. This procedure was repeated two more times. The pellet was dried in high vacuum.

3.4 mg (2.51 µmol, 1.5 eq) of peptide **3** and 2.09 mg (3.8 µmol, 2.25 eq) of PyClock were dissolved in 37.2 µL of DMF/DMSO (1:1) and 0.86 µL (5.0 µmol, 3 eq) of DIPEA were added. After 20 min the solution was added to the glycosylamine **G8**. After 39 h a solution of 2.0 mg (3.6 µmol, 2.1 eq) PyClock and 1 µL DIPEA in 10 µL DMF/DMSO (1:1) was added added. After a total of 60 h the reaction was stopped by addition of 1 mL of TFA/TES/H_2O (96:2:2). After 1 h the solution was concentrated in vacuo. The glycopeptide **B7** was

precipitated by addition of 5 mL cold diethyl ether, collected by centrifugation, washed with cold diethyl ether (3x, 5 mL) and dried in high vacuum. The residue was dissolved in 5 % acetonitrile (220 µL) and purified by RP-HPLC (Supelco Ascentis C18, 250 x 10 mm, 5 µm, gradient from 5 to 20 % acetonitrile/water, 0.1 % TFA)

Yield of **B8**: 1.65 mg (0.66 µmol, 39.8 %); ESI-MS: m/z (average isotopes): $C_{120}H_{205}N_{17}O_{74}S_2$ (3134.11); calculated $(M+3H)^{3+}$ 1045.70, $(M+2H)^{2+}$ 1568.35; found 1045.25, 1567.34.

ESI-HRMS: m/z (exact mass): $C_{120}H_{205}N_{17}O_{74}S_2$ (3132.2242); calculated: $(M+2H)^{2+}$ 1567.1194, $(M+3H)^{3+}$ 1045.0820, $(M+4H)^{4+}$ 784.0633; found: 1567.1130, 1045.0814, 784.0629.

a)

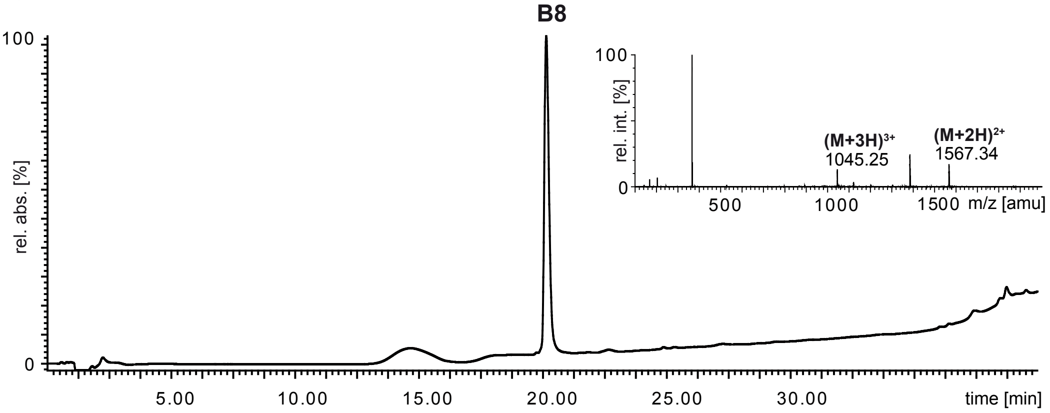

b) ^{1}H NMR, 500 MHz, D_2O + 1 % DMSO-D_6

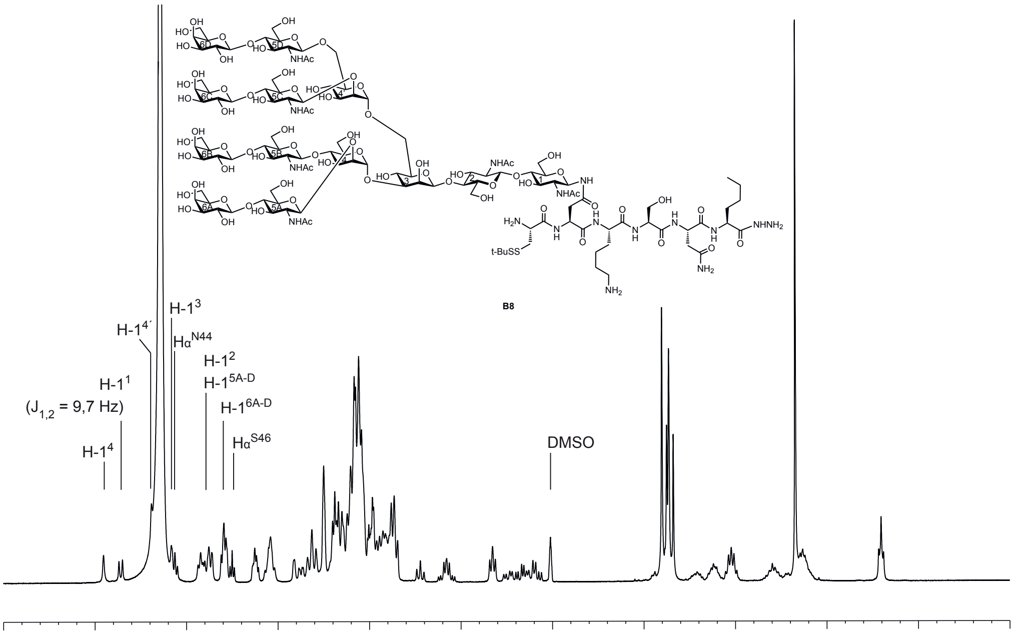

***Figure S20:** a) HPLC-MS of purified **B8**; b) partially assigned 500 MHz ^{1}H-NMR of **B8** in D_2O (DMSO-D_6 as internal reference).*

11. Synthesis of IL-6 (43-48) tetraantennary 2,6-sialylated glycopeptide B9

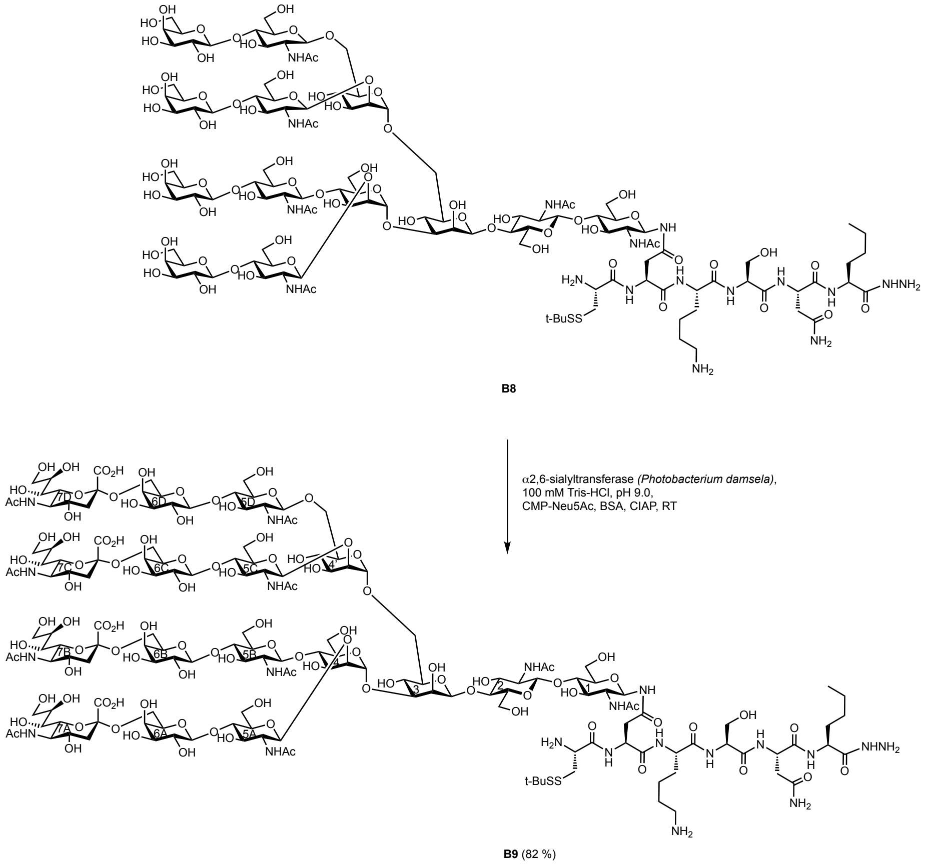

Figure S21: *Synthesis of **B9**.*

B9 was prepared by enzymatic elongation of **B8**. 3.07 mg (0.98 µmol, 1.0 eq) of **B8** and 5.8 mg (9.11 µmol, 9.3 eq) of CMP-*N*-acetyl-neuraminic acid were dissolved in 88 µL of Tris-buffer (100 mM Tris-HCl, 1 mg/ mL BSA, 20 µM NaN_3, pH 8.5). Calf-intestinal alkaline phosphatase (2.0 µL, 1000U/mL) and 10.0 µL (100 mU) of alpha-2,6-sialyltransferase from *Photobacterium damsela* were added. A pH value of 9 was maintained by periodical addition of small amounts of NaOH (0,5 µL, 5 M). CMP-*N*-acetyl-neuraminic acid was added after 17 h (5.0 mg), 46 h (2.9 mg) and 102 h (1 mg). After 102 h 2.0 µL of alpha-2,6-sialyltransferase (20 mU) and 0.5 µL of alkaline phosphatase (0.5 U) were added. After 5 days, the reaction mixture was passed over a HiLoad Superdex 30 column (600x16 mm, 0.1 M NH_4CO_3, 0.75 mL/ min) and lyophilized.

Yield of **B9**: 3.45 mg (0.80 µmol, 81.9 %); ESI-MS: m/z (average isotopes): $C_{164}H_{273}N_{21}O_{106}S_2$ (4299.13); calculated $(M+3H)^{3+}$ 1434.04, $(M+2H)^{2+}$ 2150.57; found

1433.72, 2149.96; ESI-HRMS: m/z (exact mass): $C_{164}H_{273}N_{21}O_{106}S_2$ (4296.6059); calculated: $(M+3H)^{3+}$ 1433.2092, $(M+4H)^{4+}$ 1075.1587, $(M+5H)^{5+}$ 860.3285; found: 1433.2061, 1075.1642, 860.3285.

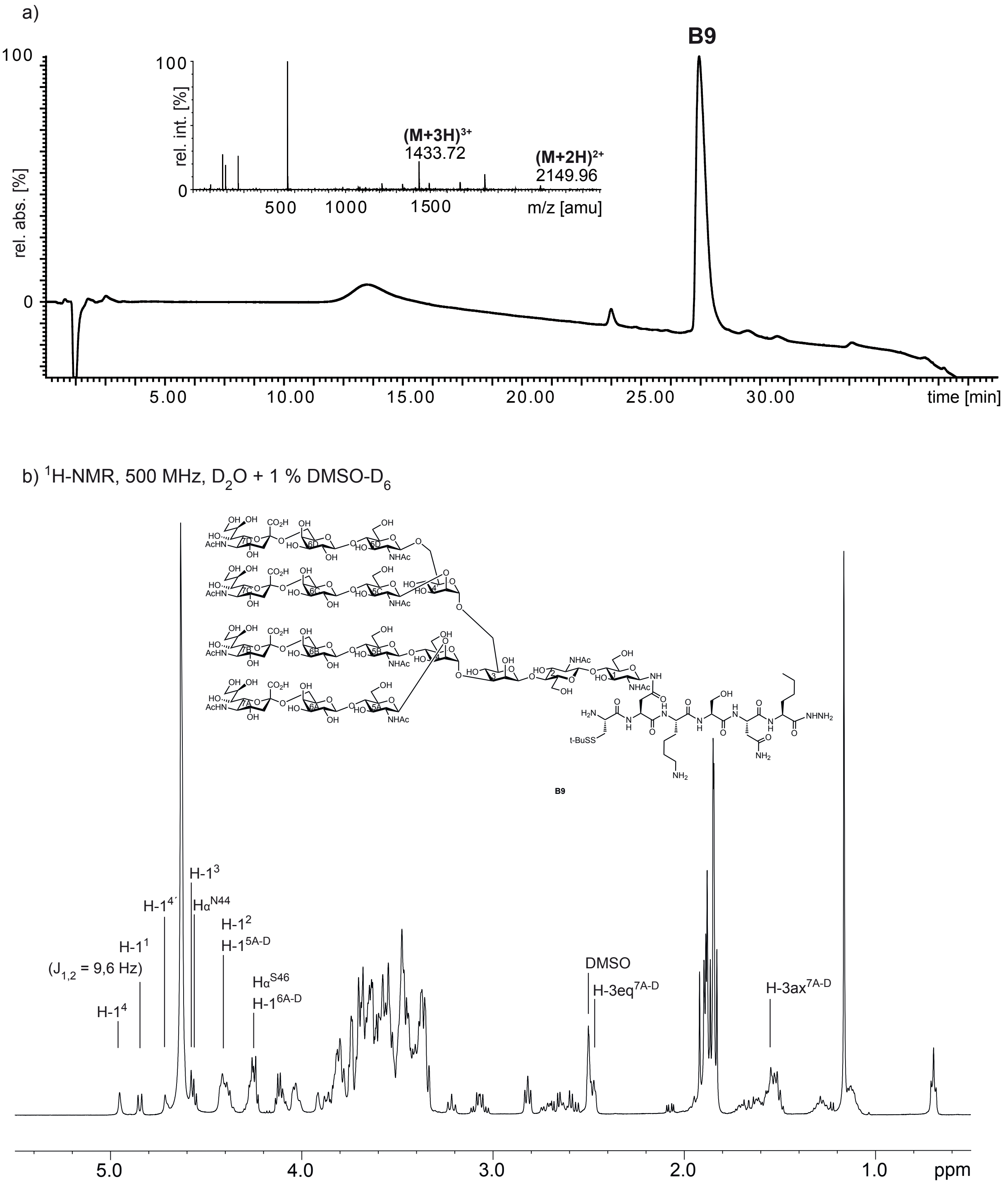

***Figure S22**: a) HPLC-MS of purified **B9**.; b) partially assigned 500 MHz ^1H-NMR of **B9** in D_2O (DMSO-D_6 as internal reference).*

12. Synthesis of 1-48 hydrazides D

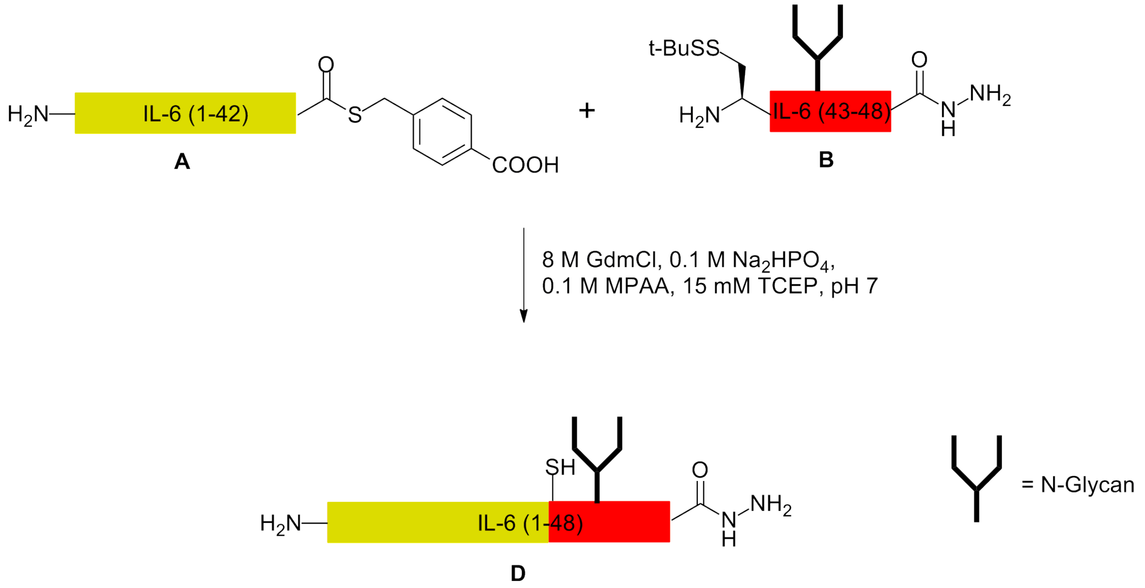

Figure S23: *Synthesis of 1-48 hydrazides* ***D***

*Synthesis of IL-6 (1-48) Man2 glycopeptide hydrazide **D3***

ligation buffer:	8 M GdmCl, 0.1 M Na_2HPO_4, 0.1 M MPAA, 15 mM TCEP, pH 7
solvent A:	H_2O, 0.1 % TFA (v/v)
solvent B:	acetonitrile, 0.1 % TFA (v/v)

The experiments were performed in an anaerobic chamber. 3.7 mg (2.5 µmol) of **B3** and 10.6 mg (2.2 µmol) of **A** were dissolved in 0.3 mL of ligation buffer and reacted for eight days. The reaction mixture was purified by RP-HPLC (YMC-Pack Protein RP 150 x 10 mm, gradient from 20 - 50 % solvent A in solvent B, flow rate 3 mL/min) and lyophilized.

Yield of **D3**: 6.3 mg (1.0 µmol; 47.1%); ESI-MS: m/z calc. (average isotopes): $C_{257}H_{430}N_{74}O_{94}S_1$ (6092.67); $(M+8H)^{8+}$ 762.58 $(M+7H)^{7+}$ 871.38, $(M+6H)^{6+}$ 1016.45, found 762.47, 871.21, 1016.26; ESI-HRMS m/z (exact mass): $C_{257}H_{430}N_{74}O_{94}S$ (6089.0863); calculated: $(M+5H)^{5+}$ 1218.8245, $(M+6H)^{6+}$ 1015.8550, $(M+7H)^{7+}$ 870.8767; found: 1218.8243, 1015.8550, 870.8782.

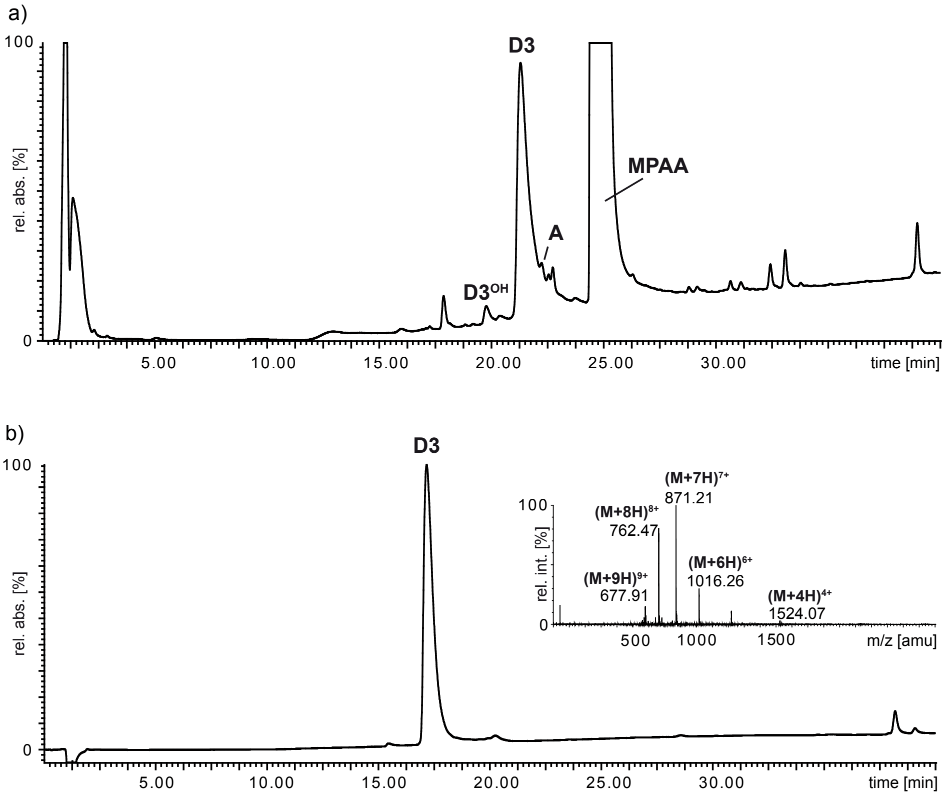

***Figure S24**: Ligation of **A** with **B3** a) HPLC chromatogram of the ligation after eight days b) HPLC-MS of **D3** after purification by HPLC.*

Synthesis of IL-6 (1-48) Man5 glycopeptide hydrazide ***D4***

ligation buffer:	8 M GdmCl, 0.1 M Na_2HPO_4, 0.1 M MPAA, 15 mM TCEP, pH 7
solvent A:	H_2O, 0.1 % TFA (v/v)
solvent B:	acetonitrile, 0.1 % TFA (v/v)

The experiments were performed in an anaerobic chamber. 1.0 mg (0.50 µmol) of **B4** and 2.9 mg (0.60 µmol) of **A** were dissolved in 0.2 mL of ligation buffer and reacted for eight days. The reaction mixture was purified by RP-HPLC (YMC-Pack Protein RP 150 x 10 mm, gradient from 20 - 50 % solvent A in solvent B, flow rate 3 mL/min) and lyophilized.

Yield of **D4**: 2.6 mg (0.40 µmol; 79.0%); ESI-MS: m/z calc. (average isotopes): $C_{275}H_{460}N_{74}O_{109}S_1$ (6579.09); $(M+7H)^{7+}$ 940.87 $(M+6H)^{6+}$ 1097.52, $(M+5H)^{5+}$ 1316.82, found 940.98, 1097.86, 1317.30.

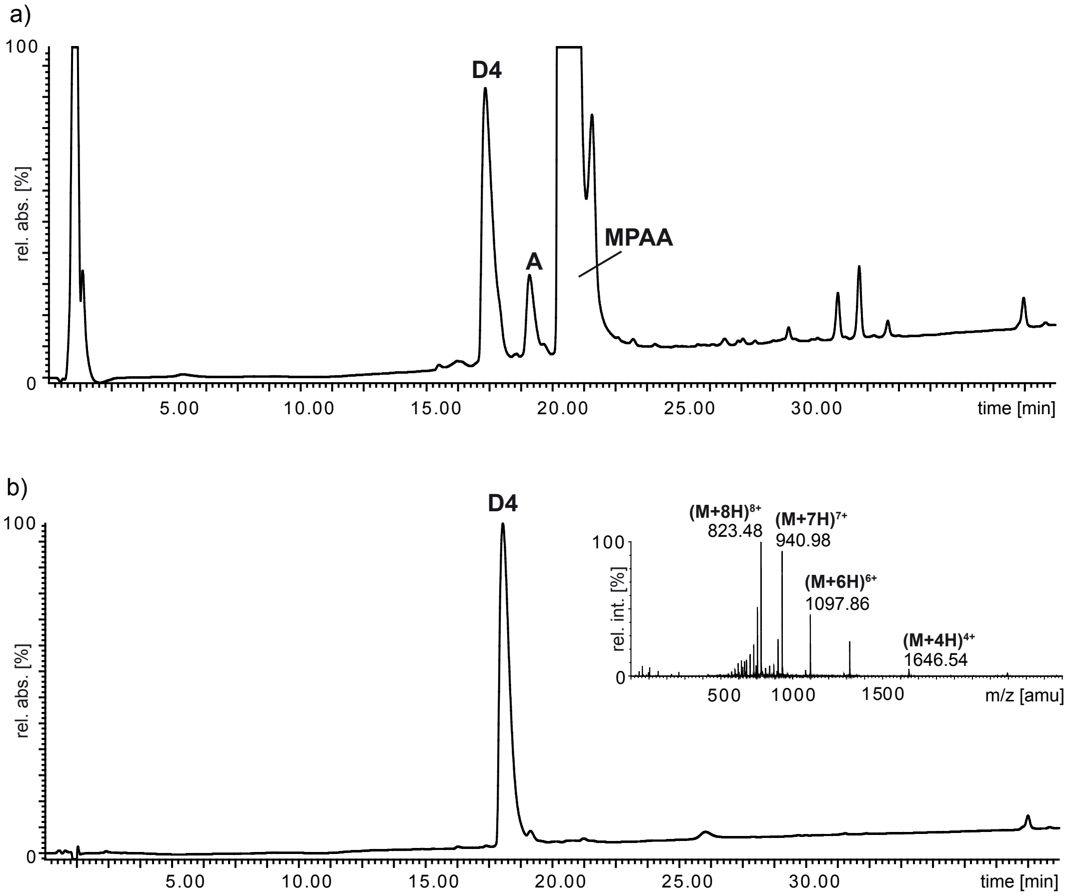

***Figure S25**: Ligation of **A** with **B4** a) HPLC chromatogram of the ligation after five days b) HPLC-MS of **D4** after purification by HPLC.*

Synthesis of IL-6 (1-48) biantennary 2,6-sialylated glycopeptide hydrazide ***D5***

ligation buffer: 8 M GdmCl, 0.1 M Na_2HPO_4, 0.1 M MPAA, 15 mM TCEP, pH 7

solvent A: H_2O, 0.1 % TFA (v/v)

solvent B: acetonitrile, 0.1 % TFA (v/v)

The experiments were performed in an anaerobic chamber. 1.3 mg (0.4 μmol) of **B5** and 2.5 mg (0.5 μmol) of **A** were dissolved in 84 μL of ligation buffer and reacted for eight days. The reaction mixture was purified by RP-HPLC (YMC-Pack Protein RP 150 x 10 mm, gradient from 20 - 50 % solvent A in solvent B, flow rate 3 mL/min). The eluate was immediately neutralized by addition of 0.1 M of NH_4HCO_3 and lyophilized. The crude hydrazide **D5** (2.5 mg) was used for the preparation of the corresponding thioester.

ESI-MS: m/z calc. (average isotopes): $C_{313}H_{520}N_{78}O_{135}S_1$ (7568.07); $(M+8H)^{8+}$ 947.00 $(M+7H)^{7+}$ 1082.14, $(M+6H)^{6+}$ 1262.33, found 947.06, 1082.25, 1262.43; ESI-HRMS: m/z (exact mass): $C_{313}H_{520}N_{78}O_{135}S_1$ (7563.5943); calculated: $(M+5H)^{5+}$ 1513.7261, $(M+6H)^{6+}$ 1261.6063, $(M+7H)^{7+}$ 1081.5208, $(M+8H)^{8+}$ 946.4566; found: 1513.7263, 1261.6069, 1081.5208, 946.4588.

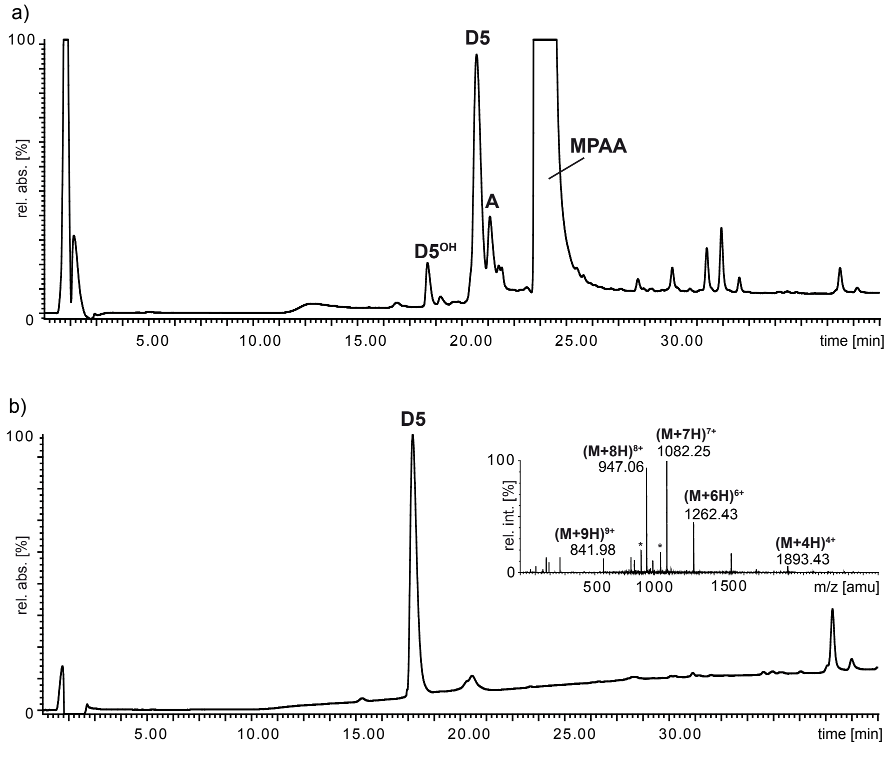

***Figure S26**: Ligation of **A** with **B5** a) HPLC chromatogram of the ligation after eight days b) HPLC-MS of **D5** after purification by HPLC (* loss of sialic acid).*

Synthesis of IL-6 (1-48) biantennary 2,3-sialylated glycopeptide hydrazide ***D6***

ligation buffer: 8 M GdmCl, 0.1 M Na_2HPO_4, 0.1 M MPAA, 15 mM TCEP, pH 7

solvent A: H_2O, 0.1 % TFA (v/v)

solvent B: acetonitrile, 0.1 % TFA (v/v)

The experiments were performed in an anaerobic chamber. 0.96 mg (0.32 µmol) of **B6** and 1.85 mg (0.38 µmol) of **A** were dissolved in 125 µL of ligation buffer and reacted for eight days. The reaction mixture was purified by RP-HPLC (YMC-Pack Protein RP 150 x 10 mm, gradient from 20 - 50 % solvent A in solvent B, flow rate 3 mL/min). The eluate was immediately neutralized by addition of 0.1 M of NH_4HCO_3 and lyophilized. The crude hydrazide **D6** (2.5 mg) was used for the preparation of the corresponding thioester.

ESI-MS: m/z calc. (average isotopes): $C_{313}H_{520}N_{78}O_{135}S_1$ (7568.07); $(M+8H)^{8+}$ 947.00 $(M+7H)^{7+}$ 1082.14, $(M+6H)^{6+}$ 1262.33, found 946.96, 1082.20, 1262.58.

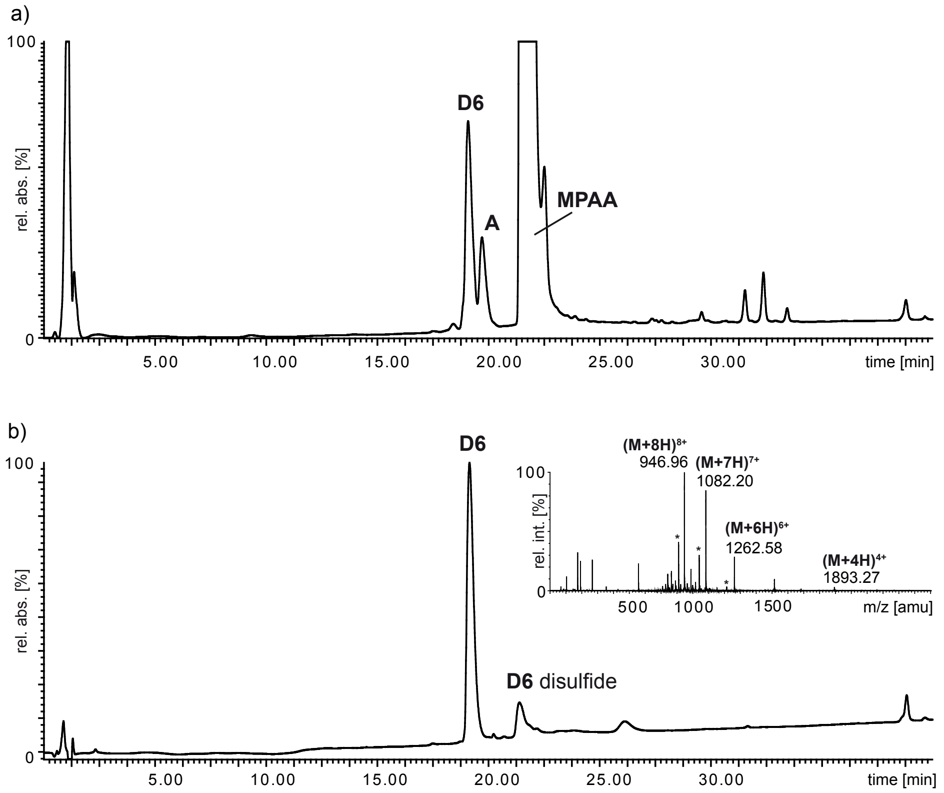

Figure S27*: Ligation of* ***A*** *with* ***B6*** *a) HPLC chromatogram of the ligation after one day b) HPLC-MS analysis of* ***D6*** *after purification by HPLC (* loss of sialic acid).*

Synthesis of IL-6 (1-48) tetraantennary glycopeptide hydrazide ***D7***

ligation buffer:	8 M GdmCl, 0.1 M Na_2HPO_4, 0.1 M MPAA, 15 mM TCEP, pH 7
solvent A:	H_2O, 0.1 % TFA (v/v)
solvent B:	acetonitrile, 0.1 % TFA (v/v)

The experiments were performed in an anaerobic chamber. 1.6 mg (0.7 µmol) of **B7** and 3.8 mg (0.8 µmol) of **A** were dissolved in 50 µL of ligation buffer and reacted for six days. The reaction mixture was purified by RP-HPLC (YMC-Pack Protein RP 150 x 10 mm, gradient from 20 - 50 % solvent A in solvent B, flow rate 3 mL/min) and lyophilized.

Yield of **D7**: 3.4 mg (0.5 µmol; 73.4%); ESI-MS: m/z calc. (average isotopes): $C_{295}H_{492}N_{78}O_{119}S_1$ (7067.58); $(M+8H)^{8+}$ 884.45 $(M+7H)^{7+}$ 1010.65, $(M+6H)^{6+}$ 1178.93, found 884.22, 1010.48, 1178.74; ESI-HRMS: m/z (exact mass): $C_{295}H_{492}N_{78}O_{119}S_1$ (7063.4566); calculated: $(M+5H)^{5+}$ 1413.6986, $(M+6H)^{6+}$ 1178.2500, $(M+7H)^{7+}$ 1010.0725, $(M+8H)^{8+}$ 883.9394; found: 1413.6972, 1178.2498, 1010.0725, 883.9401.

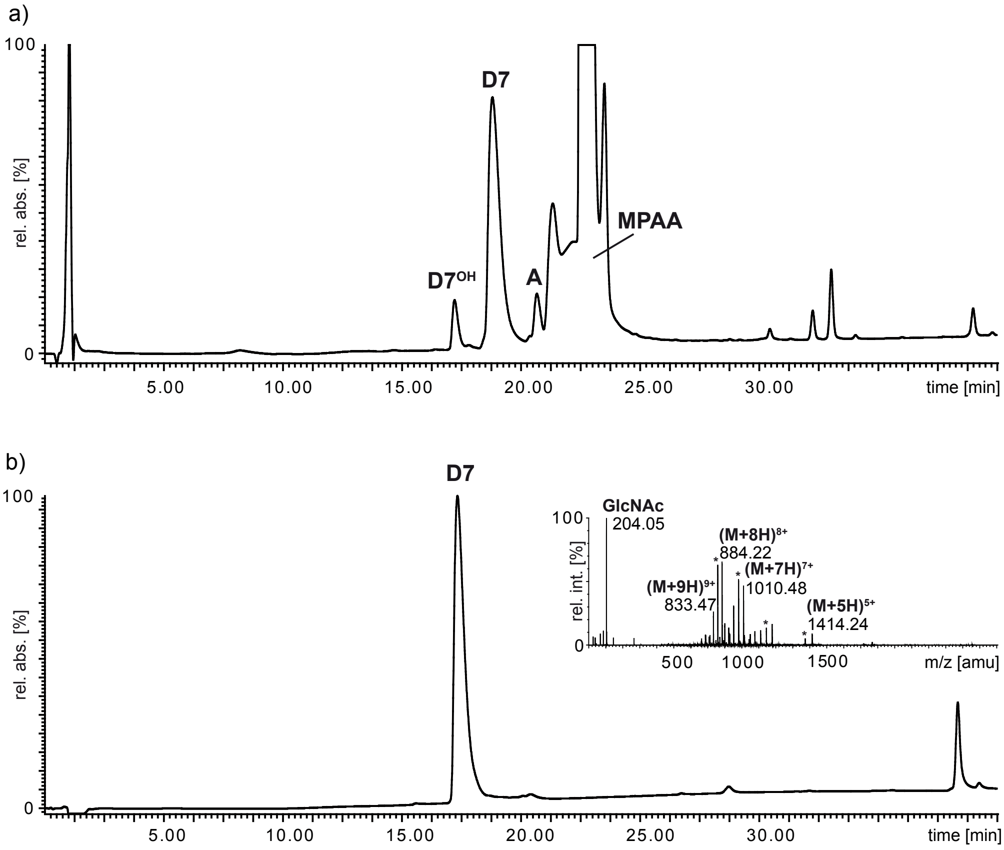

Figure S31*: Ligation of* ***A*** *with* ***B7*** *a) HPLC chromatogram of the ligation after 6 days b) HPLC-MS analysis of* ***D7*** *after purification by HPLC (* loss of GlcNAc).*

Synthesis of IL-6 (1-48) tetraantennary galactosylated glycopeptide hydrazide ***D8***

ligation buffer:	8 M GdmCl, 0.1 M Na_2HPO_4, 0.1 M MPAA, 15 mM TCEP, pH 7
solvent A:	H_2O, 0.1 % TFA (v/v)
solvent B:	acetonitrile, 0.1 % TFA (v/v)

The experiments were performed in an anaerobic chamber. 2.3 mg (0.7 µmol) of **B8** and 4.1 mg (0.8 µmol) of **A** were dissolved in 75 µL of ligation buffer and reacted for eight days. The reaction mixture was diluted by addition of 100 µl of 50 % solvent B in solvent A and immediately passed over a Superdex Peptide 10/300 GL column (30 % acetonitrile/ H_2O, 0.1 % TFA, flow rate 1.0 mL/min column. The eluate was lyophilized and the residue was dissolved in 100 µl of solvent A, purified by RP-HPLC (YMC-Pack Protein RP 150 x 10 mm, gradient from 20 - 50 % solvent A in solvent B, flow rate 3 mL/min) and lyophilized.

Yield of **D8**: 2.8 mg (0.5 µmol; 75.4%); ESI-MS: m/z calc. (average isotopes): $C_{319}H_{532}N_{78}O_{139}S_1$ (7716.14); $(M+8H)^{8+}$ 965.52 $(M+7H)^{7+}$ 1103.31, $(M+6H)^{6+}$ 1287.02, found 965.33, 1103.19, 1286.90; ESI-HRMS: m/z (exact mass): $C_{319}H_{532}N_{78}O_{139}S_1$ (7711.6679); calculated: $(M+5H)^{5+}$ 1543.3409, $(M+6H)^{6+}$ 1286.2853, $(M+7H)^{7+}$ 1102.6741, $(M+8H)^{8+}$ 964.9658; found: 1543.3398, 1286.2841, 1102.6740, 964.9672.

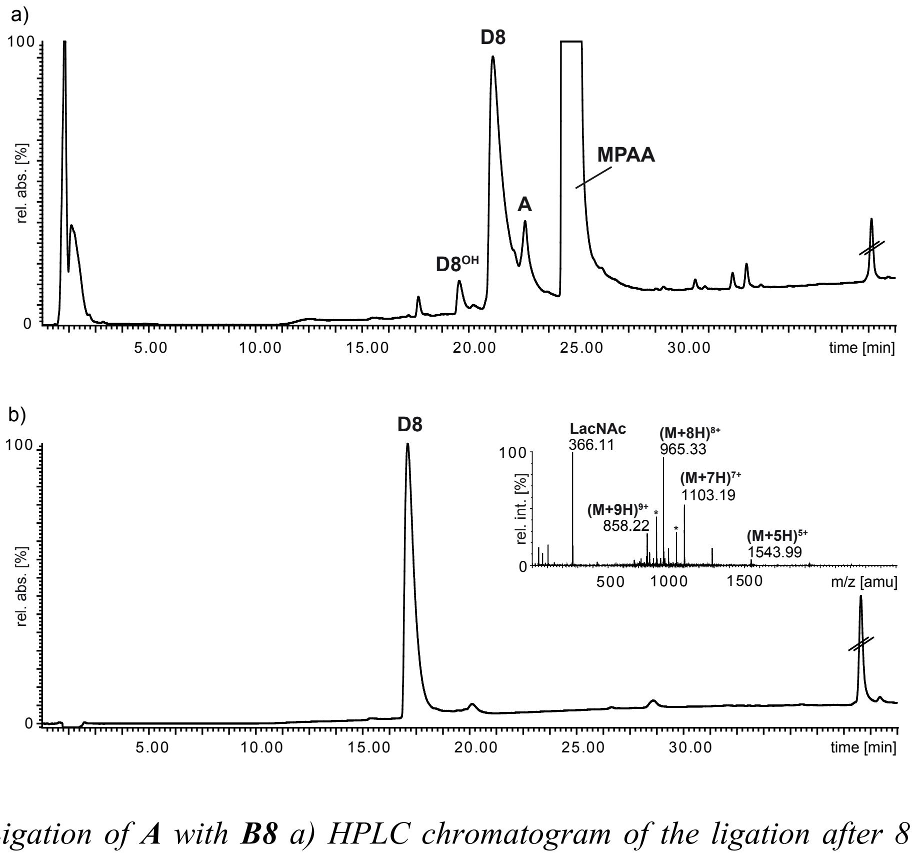

Figure S29*: Ligation of* ***A*** *with* ***B8*** *a) HPLC chromatogram of the ligation after 8 days b) HPLC-MS analysis of* ***D8*** *after purification by HPLC (* loss of LacNAc).*

Synthesis of IL-6 (1-48) tetraantennary 2,6-sialylated glycopeptide hydrazide ***D9***

ligation buffer:	8 M GdmCl, 0.1 M Na_2HPO_4, 0.1 M MPAA, 15 mM TCEP, pH 7
solvent A:	H_2O, 0.1 % TFA (v/v)
solvent B:	acetonitrile, 0.1 % TFA (v/v)

The experiments were performed in an anaerobic chamber. 1.8 mg (0.4 µmol) of **B9** and 2.5 mg (0.5 µmol) of **A** were dissolved in 0.2 mL of ligation buffer and reacted for four days. 6 mg (final concentration 0.2 M) of DTT were added and the pH was adjusted to pH 8.5 to hydrolyze remaining **A**, as **A** coeluted with **D9** during RP-HPLC. After incubation for one day the reaction mixture was purified by RP-HPLC (YMC-Pack Protein RP 150 x 10 mm, gradient from 20 - 50 % solvent A in solvent B, flow rate 3 mL/min). The eluate was immediately neutralized by addition of 0.1 M of NH_4HCO_3 and lyophilized. The crude hydrazide **D9** (2.8 mg) was used for the preparation of the corresponding thioester.

ESI-MS: m/z calc. (average isotopes): $C_{363}H_{600}N_{82}O_{171}S_1$ (8881.16); $(M+8H)^{8+}$ 1111.15 $(M+7H)^{7+}$ 1269.74, $(M+6H)^{6+}$ 1481.19, found 1111.05, 1269.64, 1481.22.

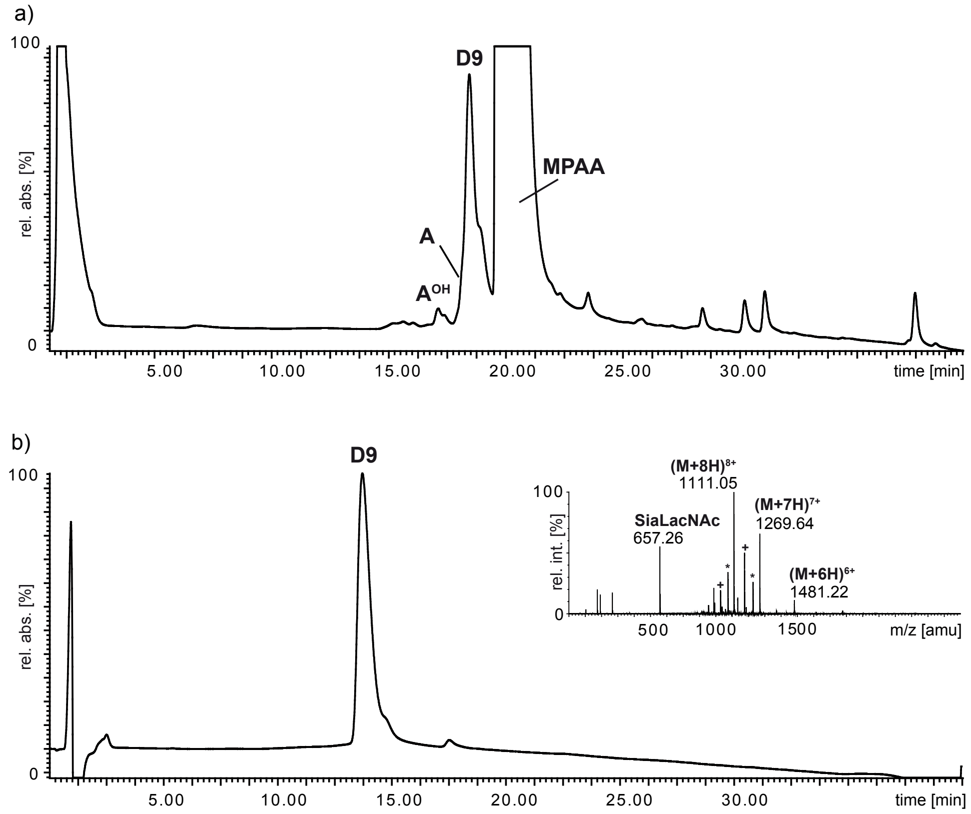

***Figure S30**: Ligation of **A** with **B9** a) HPLC chromatogram of the ligation after 4 days b) HPLC-MS analysis of **D9** after purification by HPLC (* loss of sialic acid; + loss of SiaLacNAc).*

13. Synthesis of IL-6 1-48 thioesters

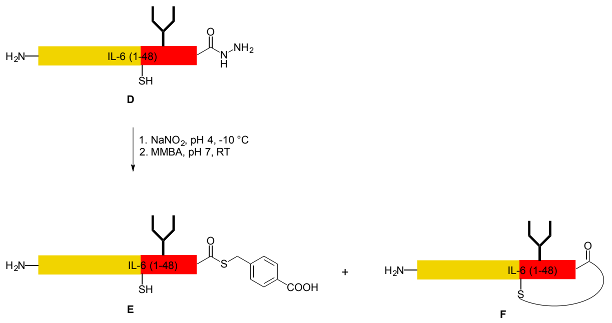

***Figure S33**: Thioesterification of 1-48 hydrazides **D** yields a mixture of thioesters **E** and thiolactones **F**.*

*Synthesis of IL-6 (1-48) Man2 glycopeptide MMBA thioester **E3***

diazotation buffer: 6 M GdmCl, 0.2 M Na_2HPO_4, 0.02 M $NaNO_2$, pH 4

A portion of 2.8 mg (0.45 µmol) of **D3** was dissolved in 200 µl of diazotation buffer at -10 °C and kept for 15 min. Subsequently, a solution of 6.7 mg (40 µmol) of MMBA in 50 µl of 1 N NaOH cooled to -10 °C was added and the pH of the reaction was adjusted to a value of 7 with 1 N NaOH. The reaction was left at ambient temperature for one hour and subsequently passed over a Superdex Peptide 10/300 GL column (30 % acetonitrile/ H_2O, 0.1 % TFA, flow rate 1.0 mL/min) and lyophilized. Yield: 2.3 mg (0.38 µmol; 82.1 %) of a mixture of IL-6 (1-48) tetra MMBA thioester **E3** and IL-6 (1-48) tetra thiolactone **F3**.

E3: ESI-MS: m/z (average isotopes): $C_{265}H_{434}N_{72}O_{96}S_2$ (6228.84); calculated: $(M+5H)^{5+}$ 1246.77, $(M+4H)^{4+}$ 1558.21, $(M+3H)^{3+}$) 2077.28, found: 1247.11, 1558.53, 2077.92.

F3: ESI-MS: m/z (average isotopes): $C_{257}H_{426}N_{72}O_{94}S$ (6060.62); calculated: $(M+5H)^{5+}$ 1213.13, $(M+4H)^{4+}$ 1516.16, $(M+3H)^{3+}$) 2021.21, found: 1213.71, 1516.38, 2021.83.

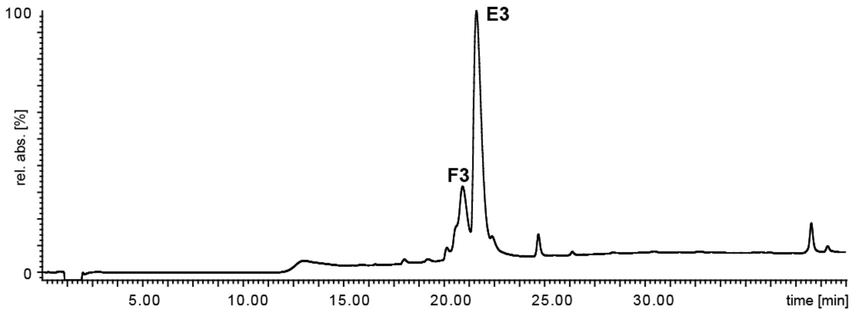

***Figure S35**: HPLC chromatogram of IL-6 (1-48) Man2 glycopeptide MMBA thioester **E3** and the corresponding thiolactone **F3** after purification by gel filtration.*

*Synthesis of IL-6 (1-48) Man5 glycopeptide MMBA thioester **E4***

diazotation buffer:	6 M GdmCl, 0.2 M Na_2HPO_4, 0.02 M $NaNO_2$, pH 4
solvent A:	H_2O, 0.1 % formic acid (v/v)
solvent B:	acetonitrile, 0.1 % formic acid (v/v)

A portion of 2.6 mg (0.40 µmol) of **D4** was dissolved in 200 µl of diazotation buffer at -10 °C and kept for 15 min. Subsequently, a solution of 6.7 mg (40 µmol) of MMBA in 50 µl of 1 N NaOH cooled to -10 °C was added and the pH of the reaction was adjusted to a value of 7 with 1 N NaOH. The reaction was left at ambient temperature for one hour and subsequently passed over a RP-HPLC column (YMC-Pack Protein RP 150 x 10 mm, gradient 20-50 % solvent B in A, flow rate 3.0 mL/min) and lyophilized. Yield: 1.8 mg (0.26 µmol; 67.5 %) of a mixture of IL-6 (1-48) Man5 MMBA thioester **E4** and IL-6 (1-48) Man5 thiolactone **F4**.

E4: ESI-MS: m/z (average isotopes): $C_{283}H_{464}N_{72}O_{111}S_2$ (6715.26); calculated: $(M+7H)^{7+}$ 960.32, $(M+6H)^{6+}$ 1120.21, $(M+5H)^{5+}$) 1344.05, found: 960.18, 1120.21, 1344.15.
F4: ESI-MS: m/z (average isotopes): $C_{275}H_{456}N_{72}O_{109}S$ (6547.05); calculated: $(M+7H)^{7+}$ 936.29, $(M+6H)^{6+}$ 1092.18, $(M+5H)^{5+}$) 1310.41, found: 936.29, 1092.30, 1310.34.

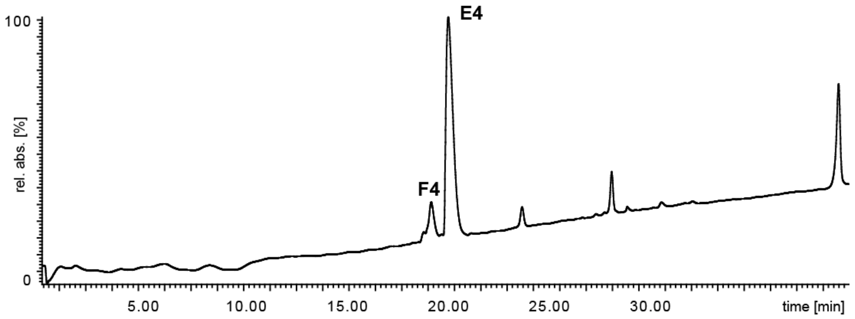

***Figure S36**: HPLC chromatogram of IL-6 (1-48) Man5 glycopeptide MMBA thioester **E4** and the corresponding thiolactone **F4** after purification by gel filtration.*

*Synthesis of IL-6 (1-48) biantennary 2,6-sialylated glycopeptide MMBA thioester **E5***

diazotation buffer: 6 M GdmCl, 0.2 M Na_2HPO_4, 0.02 M $NaNO_2$, pH 4

A portion of 2.5 mg of crude **D5** (synthesized from 1.3 mg of **B5** and 2.5 mg of **A** was dissolved in 100 µl of diazotation buffer at -10 °C and kept at this temperature for 15 min. Subsequently, a solution of 3.4 mg (20 µmol) of MMBA in 25 µl of 1 N NaOH cooled to -10 °C was added and the pH of the reaction was adjusted to a value of 7 with 1 N NaOH. The reaction was kept at ambient temperature for one hour and subsequently passed over a Superdex Peptide 10/300 GL column (30 % acetonitrile/ H_2O, 0.1 % acetic acid, flow rate 1.0 mL/min) and lyophilized. Yield: 1.8 mg (0.24 µmol; 56.6 % over 2 steps from **B5**) of a mixture of IL-6 (1-48) MMBA thioester **E5** and IL-6 (1-48) thiolactone **F5**.

E5: ESI-MS: m/z (average isotopes): $C_{321}H_{524}N_{76}O_{137}S_2$ (7704.15); calculated: $(M+7H)^{7+}$ 1101.49, $(M+6H)^{6+}$ 1285.03, $(M+5H)^{5+}$) 1541.83, found: 1101.21, 1284.78, 1541.48.
F5: ESI-MS: m/z (average isotopes): $C_{313}H_{516}N_{76}O_{135}S$ (7535.94); calculated: $(M+7H)^{7+}$ 1077.56, $(M+6H)^{6+}$ 1256.99, $(M+5H)^{5+}$) 1508.19, found: 1077.27, 1256.69, 1507.97.

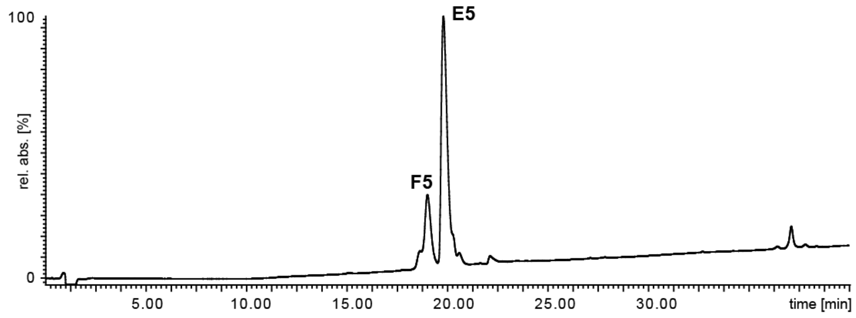

***Figure S37**: HPLC chromatogram of IL-6 (1-48) biantennary 2,6-sialylated glycopeptide MMBA thioester **E5** and the corresponding thiolactone **F5** after purification by gel filtration.*

*Synthesis of IL-6 (1-48) biantennary 2,3-sialylated glycopeptide MMBA thioester **E6***

diazotation buffer: 6 M GdmCl, 0.2 M Na_2HPO_4, 0.02 M $NaNO_2$, pH 4

solvent A: H_2O, 0.1 % formic acid (v/v)

solvent B: acetonitrile, 0.1 % formic acid (v/v)

A portion of approximately 1.3 mg (0.15 µmol) of crude **D6** was dissolved in 50 µl of diazotation buffer at -10 °C and kept at this temperature for 15 min. Subsequently, a solution of 1.7 mg (10 µmol) of MMBA in 12 µl of 1 N NaOH cooled to -10 °C was added and the pH of the reaction was adjusted to a value of 7 with 1 N NaOH. The reaction was left at ambient temperature for one hour and immediately passed over a RP-HPLC column (YMC-Pack Protein RP 150 x 10 mm, gradient 20-50 % solvent B in A, flow rate 3.0 mL/min) and lyophilized. Yield: 0.5 mg (0.07 µmol; 22 % over 2 steps from **B6**) of a mixture of IL-6 (1-48) MMBA thioester **E6** and IL-6 (1-48) thiolactone **F6**.

E6: ESI-MS: m/z (average isotopes): $C_{321}H_{524}N_{76}O_{137}S_2$ (7704.15); calculated: $(M+7H)^{7+}$ 1101.49, $(M+6H)^{6+}$ 1285.03, $(M+5H)^{5+}$) 1541.83, found: 1102.01, 1285.67, 1542.64.
F6: ESI-MS: m/z (average isotopes): $C_{313}H_{516}N_{76}O_{135}S$ (7535.94); calculated: $(M+7H)^{7+}$ 1077.56, $(M+6H)^{6+}$ 1256.99, $(M+5H)^{5+}$) 1508.19, found: 1078.04, 1257.52, 1508.79.

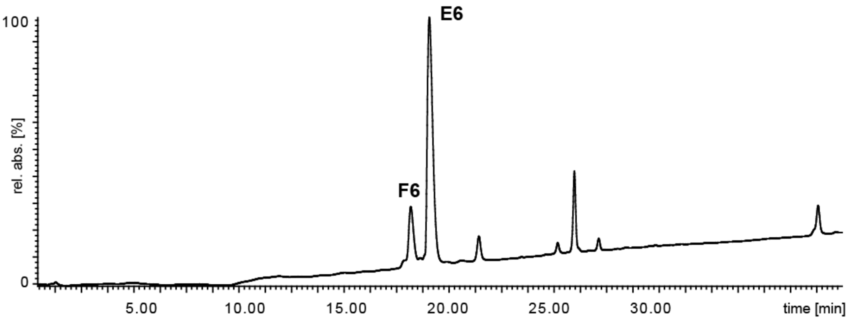

***Figure S38**: HPLC chromatogram of IL-6 (1-48) biantennary 2,3-sialylated glycopeptide MMBA thioester **E6** and the corresponding thiolactone **F6** after purification by gel filtration.*

Synthesis of IL-6 (1-48) tetraantennary glycopeptide MMBA thioester ***E7***

diazotation buffer: 6 M GdmCl, 0.2 M Na_2HPO_4, 0.02 M $NaNO_2$, pH 4

A portion of 1.5 mg (0.19 µmol) of **D7** was dissolved in 60 µl of diazotation buffer at -10 °C and kept at this temperature for 15 min. Subsequently, a solution of 2.0 mg (12 µmol) of MMBA in 15 µl of 1 N NaOH cooled to -10 °C was added and the pH of the reaction was adjusted to a value of 7 with 1 N NaOH. The reaction was left at ambient temperature for one hour and subsequently passed over a Superdex Peptide 10/300 GL column (30 % acetonitrile/ H_2O, 0.1 % TFA, flow rate 1.0 mL/min) and lyophilized. Yield: 0.97 mg (0.14 µmol; 70.6 %) of a mixture of IL-6 (1-48) MMBA thioester **E7** and IL-6 (1-48) thiolactone **F7**.

E7: ESI-MS: m/z (average isotopes): $C_{303}H_{496}N_{76}O_{121}S_2$ (7203.75); calculated: $(M+7H)^{7+}$ 1030.11, $(M+6H)^{6+}$ 1201.63, $(M+5H)^{5+}$) 1441.75, found: 1030.19, 1201.79, 1442.03.
F7: ESI-MS: m/z (average isotopes): $C_{295}H_{488}N_{76}O_{119}S$ (7035.73); calculated: $(M+7H)^{7+}$ 1106.08, $(M+6H)^{6+}$ 1173.59, $(M+5H)^{5+}$) 1408.11, found: 1006.17, 1173.76, 1408.50.

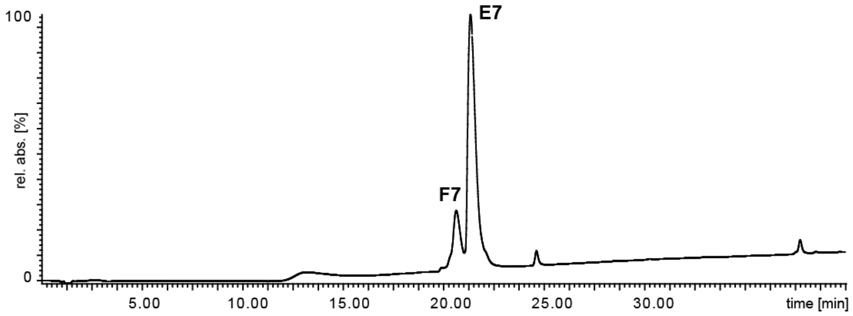

Figure S39*: HPLC chromatogram of IL-6 (1-48) tetraantennary MMBA thioester* ***E7*** *and the corresponding thiolactone* ***F7*** *after purification by gel filtration.*

*Synthesis of IL-6 (1-48) tetraantennary galactosylated glycopeptide MMBA thioester **E8***

diazotation buffer: 6 M GdmCl, 0.2 M Na_2HPO_4, 0.02 M $NaNO_2$, pH 4

A portion of 1.9 mg (0.25 µmol) of **D8** was dissolved in 100 µl of diazotation buffer at -10 °C and kept at this temperature for 15 min. Subsequently, a solution of 3.4 mg (20 µmol) of MMBA in 25 µl of 1 N NaOH cooled to -10 °C was added and the pH of the reaction was adjusted to a value of 7 with 1 N NaOH. The reaction was left at ambient temperature for one hour and immediately passed over a Superdex Peptide 10/300 GL column (30 % acetonitrile/ H_2O, 0.1 % TFA, flow rate 1.0 mL/min) and lyophilized. Yield: 1.28 mg (0.17 µmol; 67.4 %) of a mixture of IL-6 (1-48) MMBA thioester **E8** and IL-6 (1-48) thiolactone **F8**.

E8: ESI-MS: m/z (average isotopes): $C_{327}H_{536}N_{76}O_{141}S_2$ (7852.31); calculated: $(M+7H)^{7+}$ 1122.76, $(M+6H)^{6+}$ 1309.72, $(M+5H)^{5+}$) 1571.46, found: 1122.84, 1309.96, 1571.88.
F8: ESI-MS: m/z (average isotopes): $C_{319}H_{528}N_{76}O_{139}S$ (7684.10); calculated: $(M+7H)^{7+}$ 1098.73, $(M+6H)^{6+}$ 1281.68, $(M+5H)^{5+}$) 1537.82, found: 1098.85, 1281.95, 1537.95.

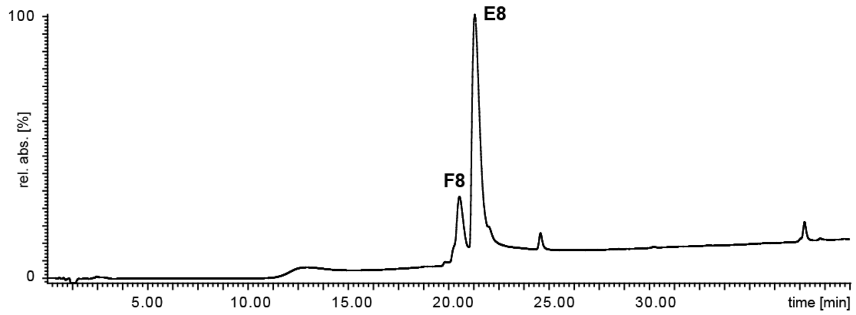

***Figure S40**: HPLC chromatogram of IL-6 (1-48) tetraantennary galactosylated glycopeptide MMBA thioester **E8** and the corresponding thiolactone **F8** after purification by gel filtration.*

*Synthesis of IL-6 (1-48) tetraantennary 2,6-sialylated glycopeptide MMBA thioester **E9***

diazotation buffer: 6 M GdmCl, 0.2 M Na_2HPO_4, 0.02 M $NaNO_2$, pH 4

A portion of 2.8 mg (0.3 µmol) of crude **D9** was dissolved in 150 µl of diazotation buffer at -10 °C. The pH was adjusted to a value of 4 with 1 N HCland the reaction was kept -10 °C for 15 min. Subsequently, a solution of 5.1 mg (30 µmol) of MMBA in 38 µl of 1 N NaOH cooled to -10 °C was added and the pH of the reaction was adjusted to a value of 7 with 1 N NaOH. The reaction was incubated at ambient temperature for one hour and immediately passed over a Superdex Peptide 10/300 GL column (30 % acetonitrile/ H_2O, 0.1 % acetic acid, flow rate 1.0 mL/min) and lyophilized. Yield: 1.9 mg (0.21 µmol; 50.3 % over 2 steps from **B9**) of a mixture of IL-6 MMBA thioester **E9** and IL-6 (1-48) thiolactone **F9**.

E9: ESI-MS: m/z (average isotopes): $C_{371}H_{604}N_{80}O_{173}S_2$ (9017.33); calculated: $(M+8H)^{8+}$ 1128.17, $(M+7H)^{7+}$ 1289.19, $(M+6H)^{6+}$) 1503.89, found: 1128.52, 1289.70, 1504.63.
F9: ESI-MS: m/z (average isotopes): $C_{363}H_{596}N_{80}O_{171}S$ (8849.11); calculated: $(M+7H)^{7+}$ 1107.14, $(M+6H)^{6+}$ 1265.16, $(M+5H)^{5+}$) 1475.85, found: 1107.54, 1265.78, 1476.45.

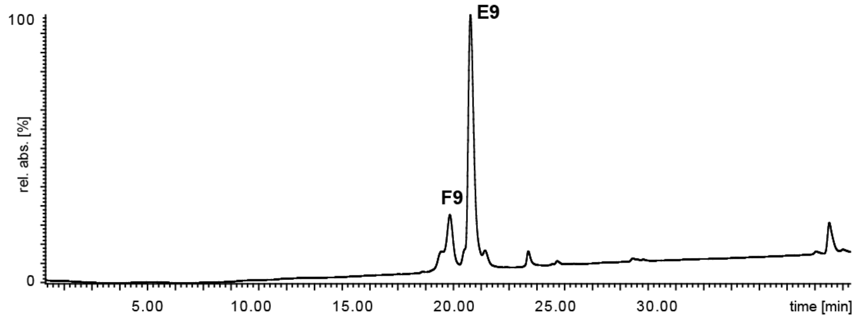

***Figure S41**: HPLC chromatogram of IL-6 (1-48) heptadeca (tetraantennary 2,6 sialylated) MMBA thioester **E9** and the corresponding thiolactone **F9** after purification by gel filtration.*

14. Ligation to 1-183 full length IL-6 and refolding to glycoforms IL-6$^{3\text{-}9}$

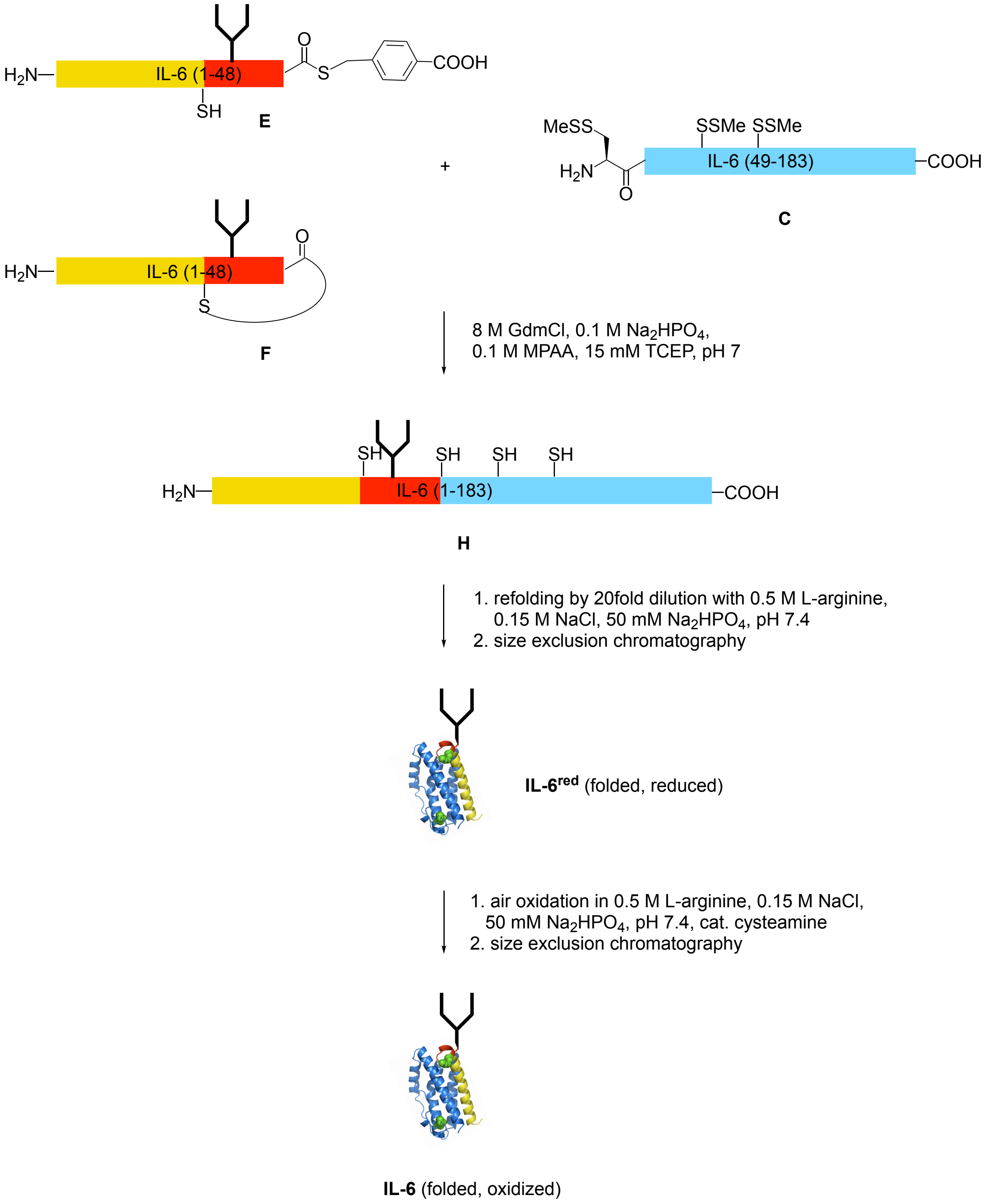

Figure S42: Ligation to 1-183 full length interleukin 6 glycopeptides **H** and refolding to glycoforms **IL-6**$^{3\text{-}9}$.

Synthesis of Man2 ***IL-6³***

ligation buffer: 8 M GdmCl, 0.1 M Na_2HPO_4, 0.1 M MPAA, 15 mM TCEP, pH 7

refolding buffer: 0.5 M arginine, 150 mM NaCl, 50 mM Na_2HPO_4, pH 8.5

The reactions were performed in an anaerobic chamber. 2.3 mg (0.35 μmol) of a mixture of **E3**/**F3** and 4.0 mg (0.26 μmol) of **C** were dissolved in 150 μl of ligation buffer. The reaction was kept for ten days at ambient temperature. The ligation mixture was subsequently reduced by addition of 4.6 mg of DTT (final concentration 200 mM) and incubated for four hours.

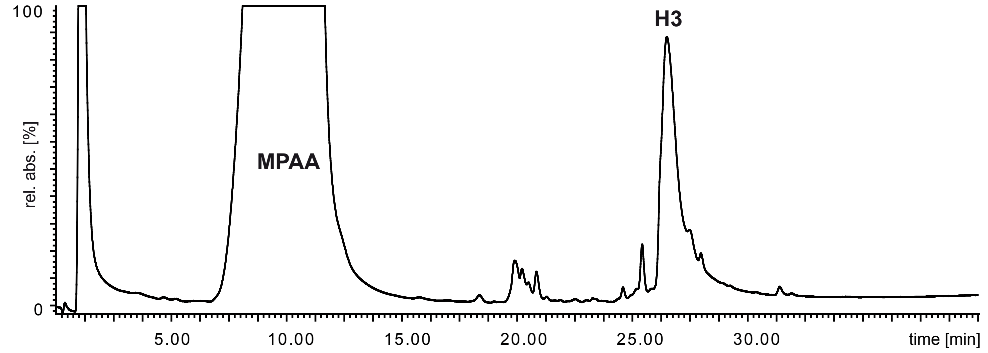

Figure S43*: HPLC-MS of the ligation of* ***E3/F3*** *with* ***C*** *after 10 days.*

The solution containing glycoprotein **H3** (total volume 150 μL) was refolded and purified in aliquots of 30 μL. A portion of 30 μL of the reduced ligation mixture was placed in a test tube and 570 μl of refolding buffer were added within three seconds during vortexing. After 30 min the test tube was removed from the anaerobic chamber, centrifuged and the supernatant was immediately passed over a HiLoad 16/60 Superdex 75 pg column (flow rate: 2 mL/min, refolding buffer).

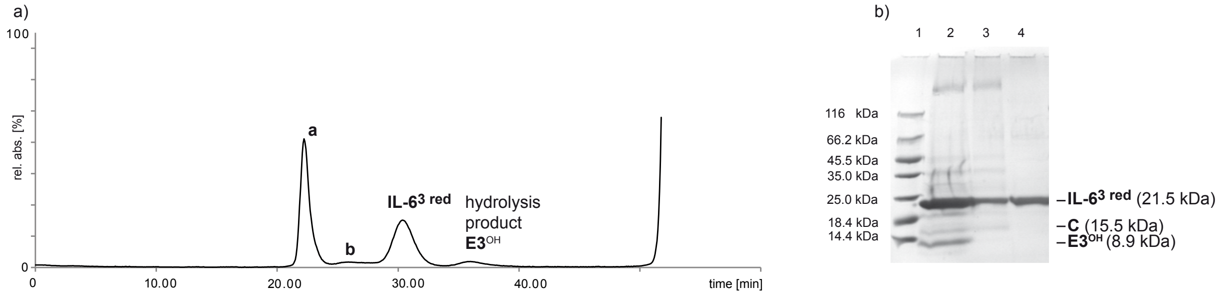

Figure S44*: a) Purification of refolded* ***IL-6³ red*** *by gel filtration; b) Reducing SDS-PAGE of the purification of* ***IL-6³ red*** *by gel filtration. Lane 1: molecular weight standard; Lane 2: crude* ***IL-6³ red****; Lane 3: Peak* ***a*** *at 22 min; Lane 4:* ***IL-6³ red*** *(peak at 31 min).*

The fraction of monomeric **IL-6^{3} ^{red}** (ca. 10 mL per 30 µL aliquot) was concentrated by ultrafiltration to 1 mL (Pierce concentrators, 20 mL, 20 K MWCO, 3800 g, 10 °C, Thermo Scientific, USA). The concentrate was transferred to a test tube and 1 µL of aqueous cysteamine (1 mM) was added. **IL-6^{3} ^{red}** was oxidized in the open tube for 4 days. Crude **IL-6^{3}** was purified over a HiLoad 16/60 Superdex 75 pg column (flow rate: 2 mL/min, refolding buffer). The fractions of monomeric **IL-6^{3}** (ca. 10 mL per 60 µL aliquot) were pooled and **IL-6^{3}** was concentrated by ultrafiltration as above.

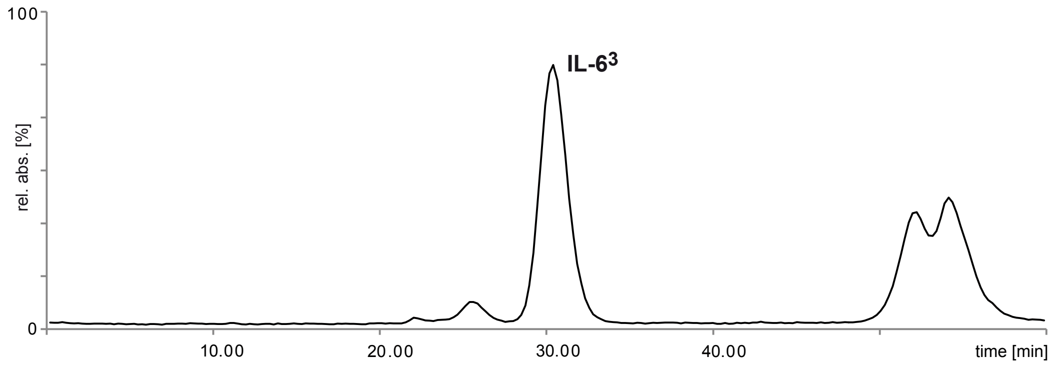

***Figure S45**: Purification of **IL-6^{3}** by gel filtration after oxidation.*

Yield of **IL-6^{3} ^{red}**: 2.0 mg (0.09 µmol; 36 %; determined by A_{280}); ESI-MS: m/z (average isotopes): $C_{938}H_{1523}N_{255}O_{306}S_{8}$ (21525.17); calculated: $(M+18H)^{18+}$ 1196.84, $(M+17H)^{17+}$ 1267.19, $(M+16H)^{16+}$) 1346.32, found: 1197.40, 1267.83, 1347.03.

Yield of **IL-6^{3}**: 1.5 mg (0.07 µmol; 73 %; determined by A_{280}); ESI-MS: m/z (average isotopes): $C_{938}H_{1519}N_{255}O_{306}S_{8}$ (21521.13); calculated: $(M+18H)^{18+}$ 1196.62, $(M+17H)^{17+}$ 1266.95, $(M+16H)^{16+}$) 1346.07, found: 1196.36, 1266.71, 1345.84; ESI-HRMS: m/z (exact mass): $C_{938}H_{1519}N_{255}O_{306}S_{8}$ (21507.8905); calculated for most abundant isotope peak: $(M+12H)^{12+}$ 1794.3341, $(M+13H)^{13+}$ 1656.4630, $(M+14H)^{14+}$ 1538.2161; found: 1794.3337, 1656.4637, 1538.2164.

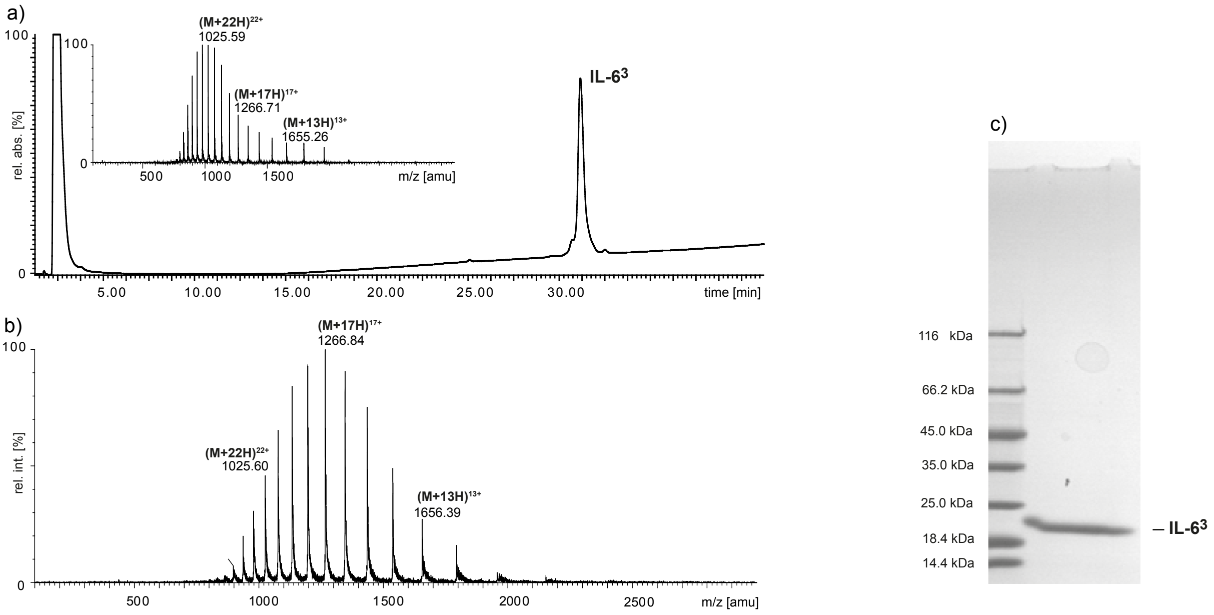

Figure S46: *Characterization of purified **IL-6**^{3}. a) HPLC-MS, b) ESI-MS (direct injection), c) SDS-PAGE*

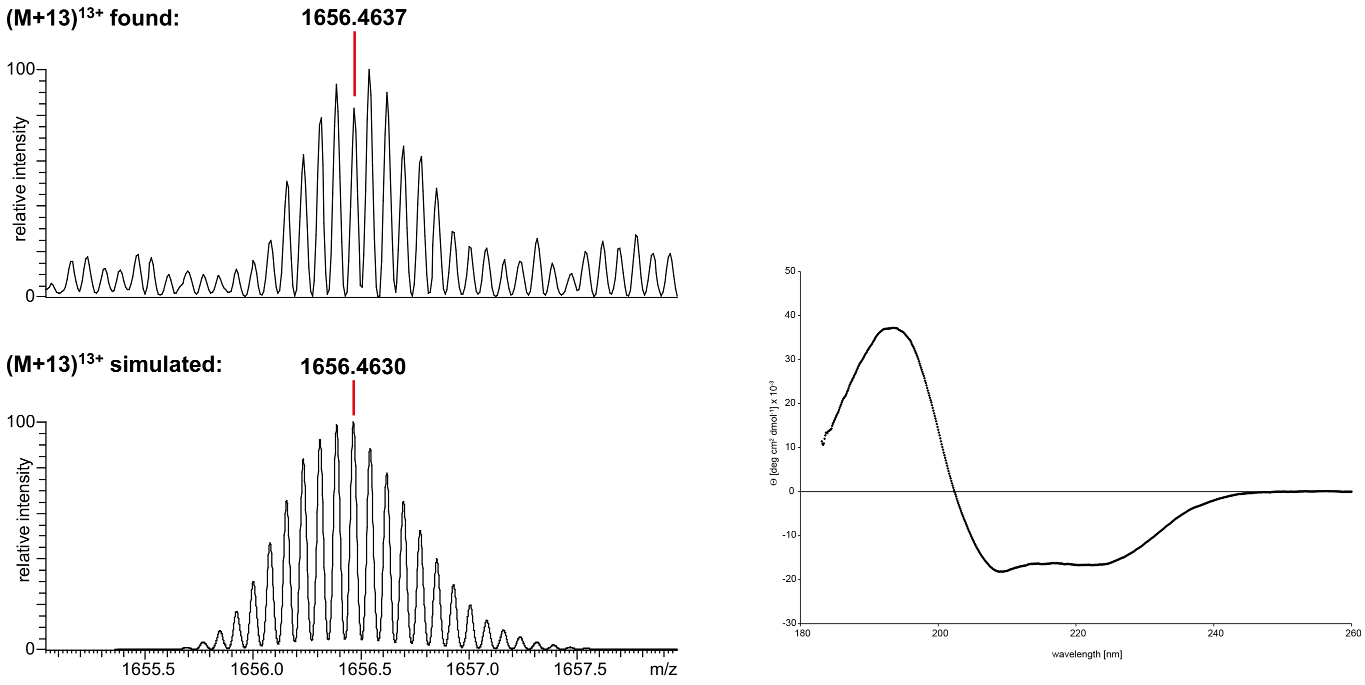

Figure S47: *a) Measured and simulated HR-MS (M+13)$^{13+}$of **IL-6**^{3}, b) CD-spectrum of purified **IL-6**^{3}.*

Synthesis of Man5 ***IL-6⁴***

ligation buffer: 8 M GdmCl, 0.1 M Na_2HPO_4, 0.1 M MPAA, 15 mM TCEP, pH 7

refolding buffer: 0.5 M arginine, 150 mM NaCl, 50 mM Na_2HPO_4, pH 8.5

The reactions were performed in an anaerobic chamber. 1.8 mg (0.27 µmol) of a mixture of **E4**/**F4** and 4.7 mg (0.30 µmol) of **C** were dissolved in 150 µl of ligation buffer. The reaction was kept for seven days at ambient temperature. The ligation mixture was subsequently reduced by addition of 4.6 mg of DTT (final concentration 200 mM) and incubated for four hours.

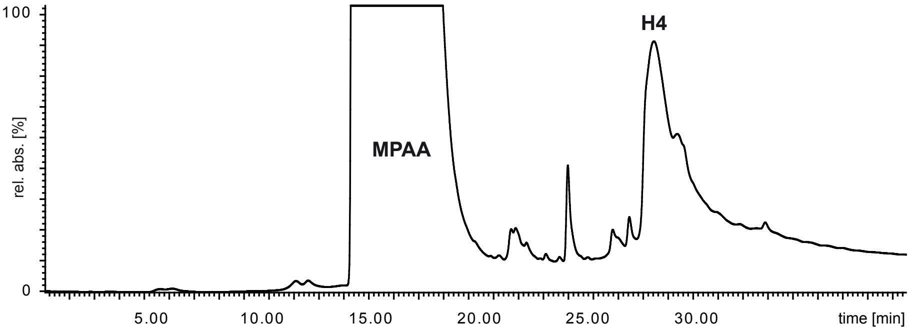

Figure S48*: HPLC-MS of the ligation of* ***E4/F4*** *with* ***C*** *after 6 days.*

The solution containing glycoprotein **H4** (total volume 150 µL) was refolded and purified in aliquots of 50 µL. A portion of 50 µL of the reduced ligation mixture was placed in a test tube and 950 µl of refolding buffer were added within three seconds during vortexing. After 30 min the test tube was removed from the anaerobic chamber, centrifuged and the supernatant was immediately passed over a HiLoad 16/60 Superdex 75 pg column (flow rate: 2 mL/min, refolding buffer).

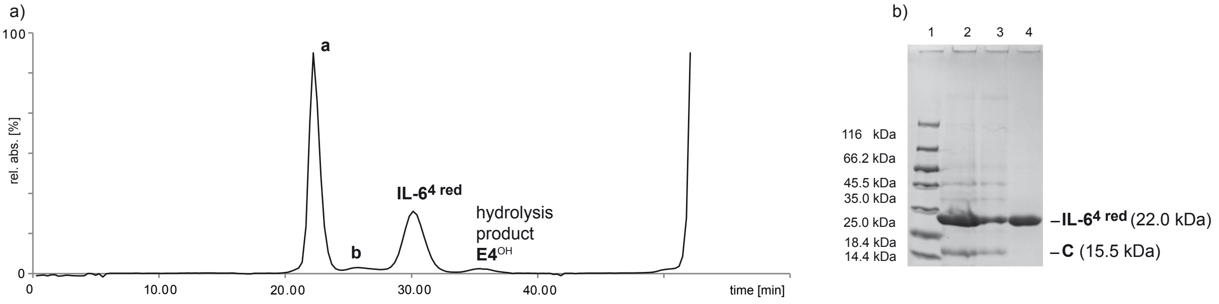

Figure S49*: a) Purification of refolded* ***IL-6⁴ red*** *by gel filtration; b) SDS-PAGE analysis of the purification of* ***IL-6⁴ red*** *by gel filtration. Lane 1: molecular weight standard; Lane 2: crude* ***IL-6⁴ red****; Lane 3: Peak* ***a*** *at 22 min; Lane 4:* ***IL-6⁴ red*** *(31 min).*

The fraction of monomeric **IL-6^{4} ^{red}** (ca. 10 mL per 50 μL aliquot) was concentrated by ultrafiltration to 1 mL (Pierce concentrators, 20 mL, 20 K MWCO, 3800 g, 10 °C, Thermo Scientific, USA). The concentrate was transferred to a test tube and 1 μL of aqueous cysteamine (1 mM) was added. **IL-6^{4} ^{red}** was oxidized in the open tube for 4 days. Crude **IL-6^{4}** was purified over a HiLoad 16/60 Superdex 75 pg column (flow rate: 2 mL/min, refolding buffer). The fractions of monomeric **IL-6^{4}** (ca. 10 mL per 60 μL aliquot) were pooled and **IL-6^{4}** was concentrated by ultrafiltration as above.

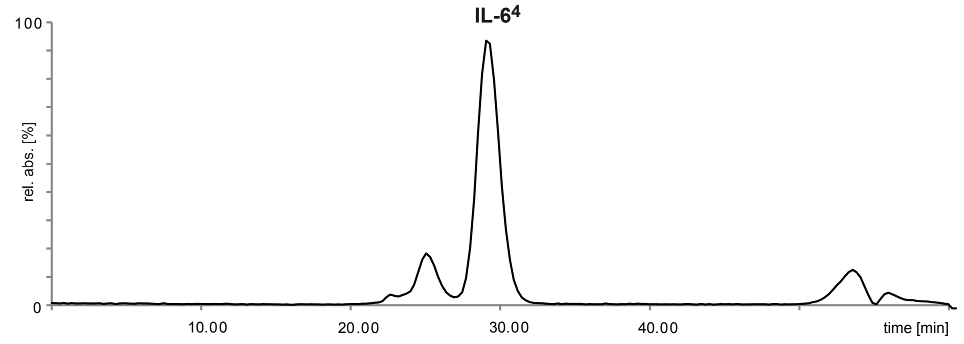

***Figure S50**: Purification of **IL-6^{4}** by gel filtration.*

Yield of **IL-6^{4} ^{red}**: 0.70 mg (0.03 μmol; 38 %; determined by A_{280}); ESI-MS: m/z (average isotopes): $C_{956}H_{1553}N_{255}O_{321}S_8$ (22011.60); calculated: $(M+18H)^{18+}$ 1223.87, $(M+17H)^{17+}$ 1295.80, $(M+16H)^{16+}$) 1376.73, found: 1224.33, 1296.22, 1377.13.

Yield of **IL-6^{4}**: 0.50 mg (0.02 μmol; 71 %; determined by A_{280}); ESI-MS: m/z (average isotopes): $C_{956}H_{1549}N_{255}O_{321}S_8$ (22007.56) calculated: $(M+18H)^{18+}$ 1223.67, $(M+17H)^{17+}$ 1295.56, $(M+16H)^{16+}$ 1376.47, found: 1224.39, 1296.28, 1377.25.

ESI-HRMS: m/z (exact mass): $C_{956}H_{1549}N_{255}O_{321}S_8$ (21994.0490); calculated for most abundant isotope peak: $(M+12H)^{12+}$ 1834.9309, $(M+13H)^{13+}$ 1693.8598, $(M+14H)^{14+}$ 1572.9417; found: 1834.9315, 1693.8604, 1572.9451.

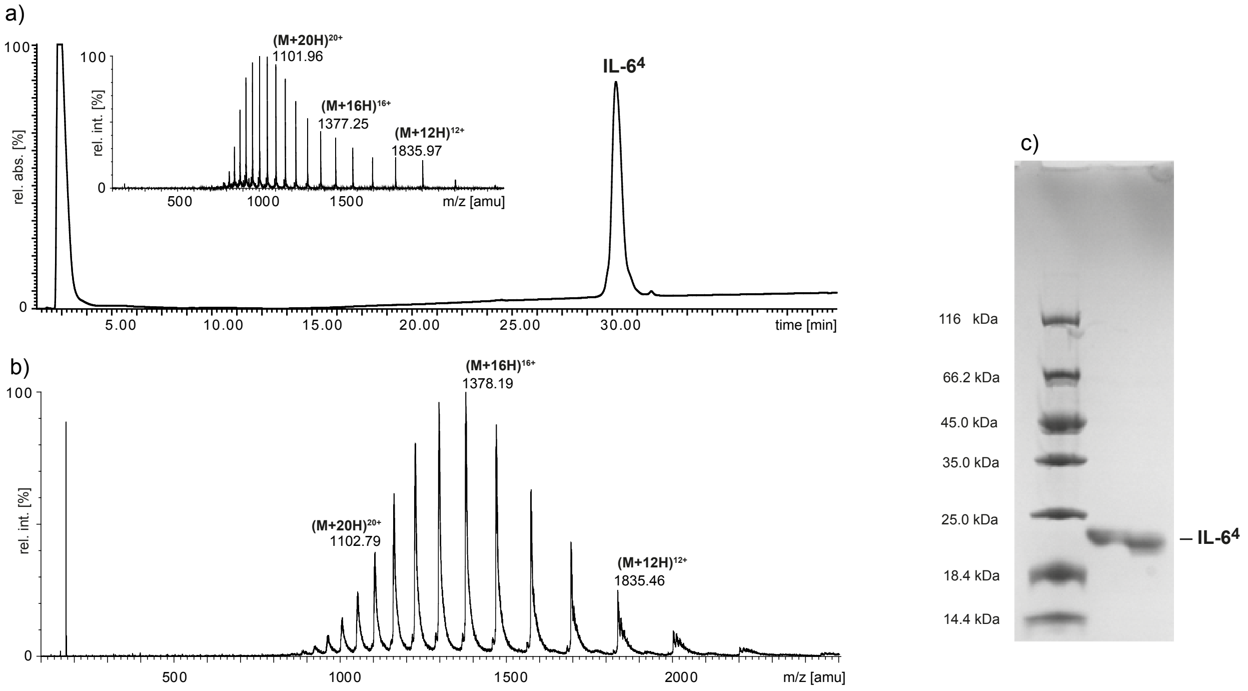

***Figure S51**: Characterization of purified **IL-6**^{4}. a) HPLC-MS, b) ESI-MS (direct injection), c) SDS-PAGE*

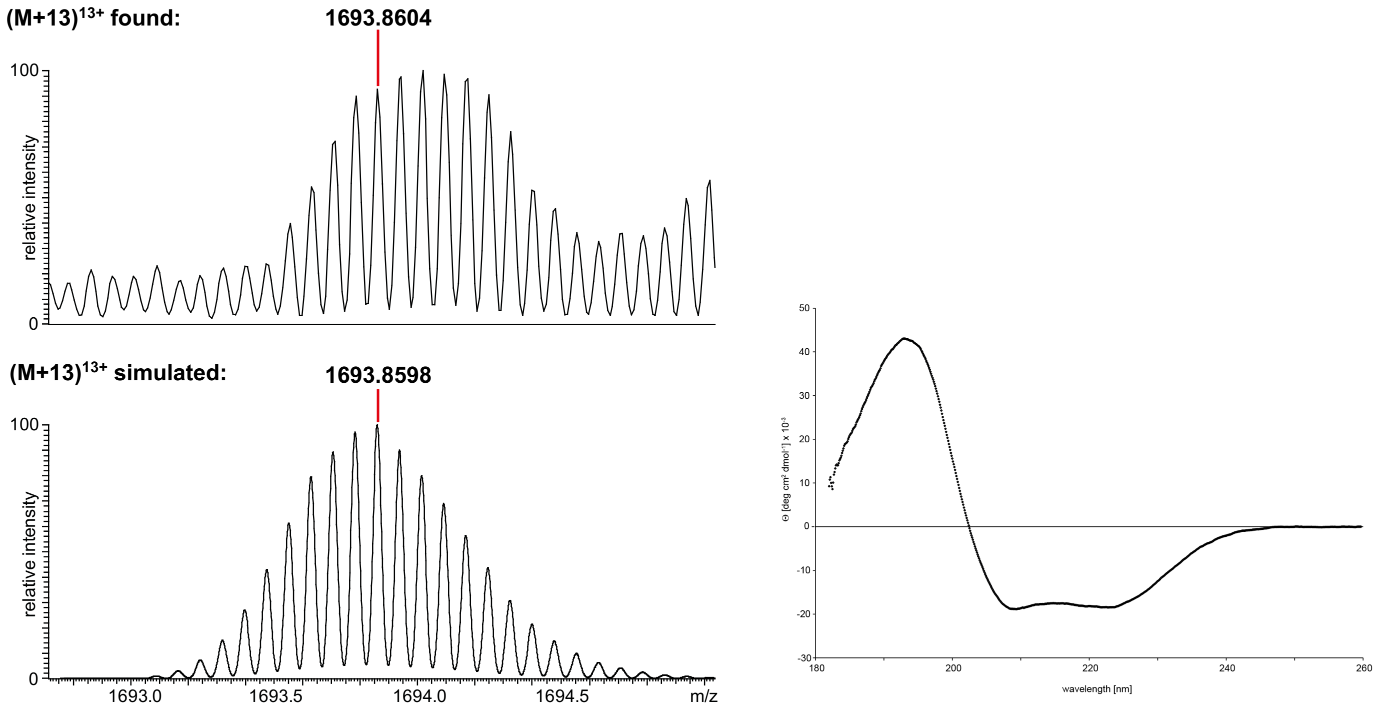

***Figure S52**: a) Measured and simulated HR-MS (M+13)$^{13+}$ of **IL-6**^{4}, b) CD-spectrum of purified **IL-6**^{4}.*

*Synthesis of biantennary 2,6-sialylated **IL-6**^{5}.*

ligation buffer: 8 M GdmCl, 0.1 M Na_2HPO_4, 0.1 M MPAA, 15 mM TCEP, pH 7

refolding buffer: 0.5 M arginine, 150 mM NaCl, 50 mM Na_2HPO_4, pH 8.5

The reactions were performed in an anaerobic chamber. 1.7 mg (0.22 µmol) of a mixture of **E5**/**F5** and 3.0 mg (0.19 µmol) of **C** were dissolved in 200 µl of ligation buffer. The reaction was kept for five days at ambient temperature. The ligation mixture was subsequently reduced by addition of 6.2 mg of DTT (final concentration 200 mM) and incubated for four hours.

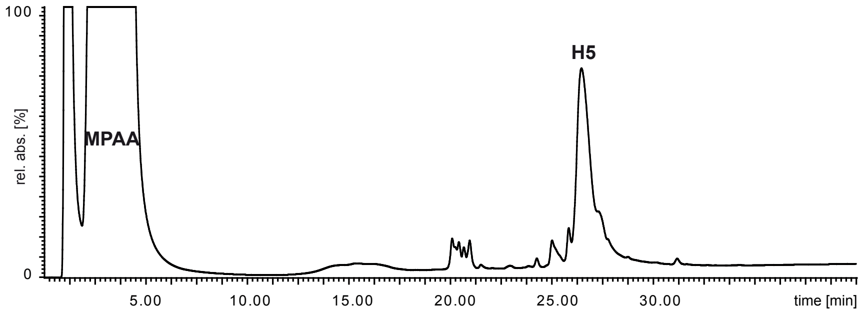

***Figure S53**: HPLC-MS of the ligation of **E5/F5** with **C** after 5 days.*

The solution containing glycoprotein **H 5** (total volume 200 µL) was refolded and purified in aliquots of 50 µL. A portion of 50 µL of the reduced ligation mixture was placed in a test tube and 950 µl of refolding buffer were added within three seconds during vortexing. After 30 min the test tube was removed from the anaerobic chamber, centrifuged and the supernatant was immediately passed over a HiLoad 16/60 Superdex 75 pg column (flow rate: 2 mL/min, refolding buffer).

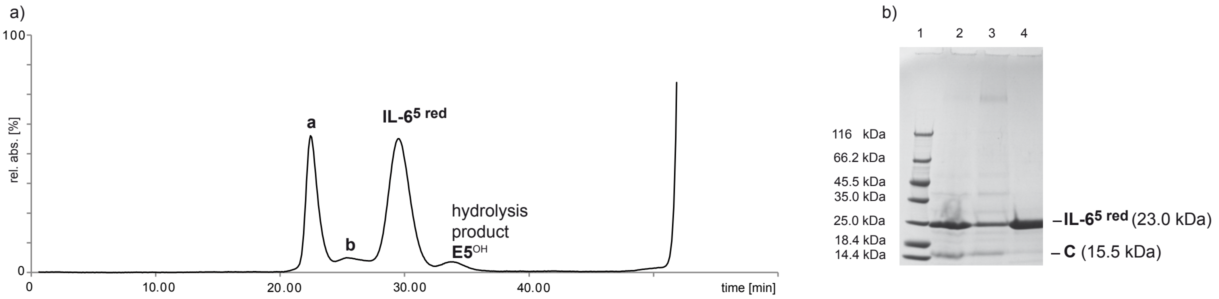

***Figure S54**: a) Purification of refolded **IL-6**^{5} **red** by gel filtration; b) SDS-PAGE analysis of the purification of **IL-6**^{5} **red** by gel filtration. Lane 1: molecular weight standard; Lane 2: crude **IL-6**^{5} **red**; Lane 3: Peak **a** at 22 min; Lane 4: **IL-6**^{5} **red** (29 min).*

The fraction of monomeric **IL-6^{5} red** (ca. 10 mL per 50 µL aliquot) was concentrated by ultrafiltration to 1 mL (Pierce concentrators, 20 mL, 20 K MWCO, 3800 g, 10 °C, Thermo Scientific, USA). The concentrate was transferred to a test tube and 1 µL of aqueous cysteamine (1 mM) were added. **IL-6^{5} red** was oxidized in the open tube for 4 days. Crude **IL-6^{5}** was purified over a HiLoad 16/60 Superdex 75 pg column (flow rate: 2 mL/min, refolding buffer). The fractions of monomeric **H5** (ca. 10 mL per 60 µL aliquot) were pooled and **H5** was concentrated by ultrafiltration as above.

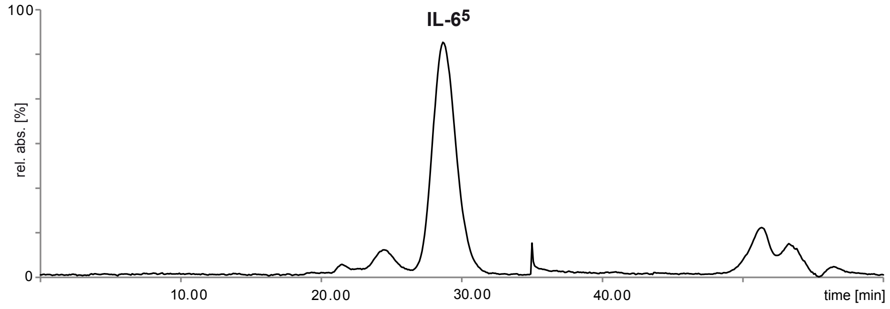

***Figure S55**: Purification of **IL-6^{5}** by gel filtration.*

Yield of **IL-6^{5} red**: 1.97 mg (0.09 µmol; 44 %; determined by A_{280}); ESI-MS: m/z (average isotopes): $C_{994}H_{1613}N_{259}O_{347}S_{8}$ (23000.49); calculated: $(M+18H)^{18+}$ 1278.81, $(M+17H)^{17+}$ 1353.97, $(M+16H)^{16+}$) 1438.53, found: 1278.62, 1353.79, 1438.34.

Yield of **H5**: 1.52 mg (0.07 µmol; 77 %; determined by A_{280}); ESI-MS: m/z (average isotopes): $C_{994}H_{1609}N_{259}O_{347}S_{8}$ (22996.45); calculated: $(M+18H)^{18+}$ 1278.58, $(M+17H)^{17+}$ 1353.73, $(M+16H)^{16+}$) 1438.28, found: 1278.65, 1353.81, 1438.39.

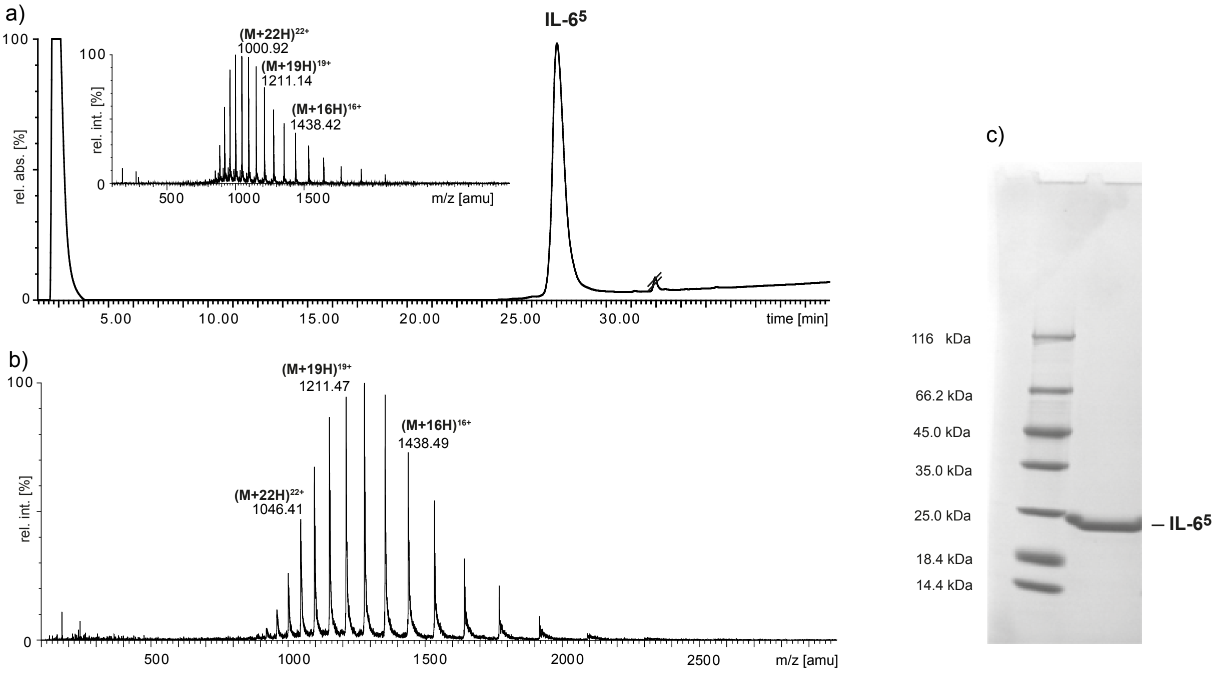

***Figure S56**: Characterization of purified **IL-6**^{5}. a) HPLC-MS, b) ESI-MS (direct injection), c) SDS-PAGE.*

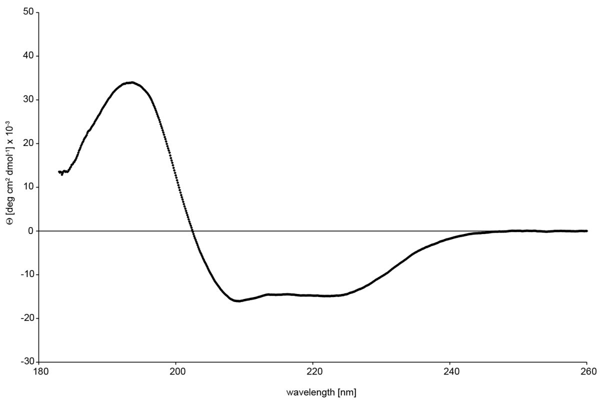

***Figure S57**: CD-spectrum of purified **IL-6**^{5}.*

*Synthesis of biantennary 2,3-sialylated **IL-6⁶***

ligation buffer: 8 M GdmCl, 0.1 M Na_2HPO_4, 0.1 M MPAA, 15 mM TCEP, pH 7

refolding buffer: 0.5 M arginine, 150 mM NaCl, 50 mM Na_2HPO_4, pH 8.5

The reactions were performed in an anaerobic chamber. 0.47 mg (0.06 µmol) of a mixture of **E6**/**F6** and 1.1 mg (0.07 µmol) of **C** were dissolved in 37 µl of ligation buffer. The reaction was kept for seven days at ambient temperature. The ligation mixture was subsequently reduced by addition of 1.0 mg of DTT (final concentration 200 mM) and incubated for four hours.

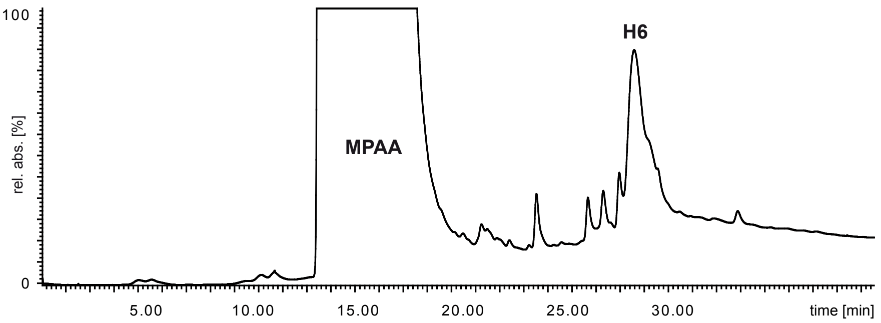

***Figure S58**: HPLC-MS of the ligation of **E6/F6** with **C** after 6 days.*

The reduced ligation mixture, containing the glycoprotein **H6** (total volume 37 µL) was placed in a test tube and 760 µl of refolding buffer were added within three seconds during vortexing. After 30 min the test tube was removed from the anaerobic chamber, centrifuged and the supernatant was immediately passed over a HiLoad 16/60 Superdex 75 pg column (flow rate: 2 mL/min, refolding buffer).

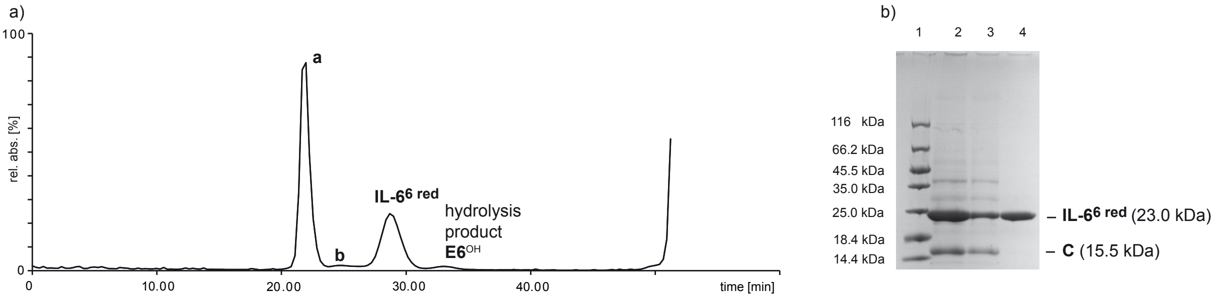

***Figure S59**: a) Purification of refolded **IL-6⁶ red** by gel filtration; b) SDS-PAGE analysis of the purification of **IL-6⁶ red** by gel filtration. Lane 1: molecular weight standard; Lane 2: crude **IL-6⁶ red**; Lane 3: Peak **a** at 22 min; Lane 4: **IL-6⁶ red** (29 min).*

The fraction of monomeric **IL-6^{6} red** (ca. 10 mL per 50 µL aliquot) was concentrated by ultrafiltration to 1 mL (Pierce concentrators, 20 mL, 20 K MWCO, 3800 g, 10 °C, Thermo Scientific, USA). The concentrate was transferred to a test tube and 1 µL of aqueous cysteamine (1 mM) were added. **IL-6^{6} red** was oxidized in the open tube for 4 days. Crude **IL-6^{6}** was purified over a HiLoad 16/60 Superdex 75 pg column (flow rate: 2 mL/min, refolding buffer). The fractions of monomeric **IL-6^{6}** (ca. 10 mL per 60 µL aliquot) were pooled and **IL-6^{6}** was concentrated by ultrafiltration as above.

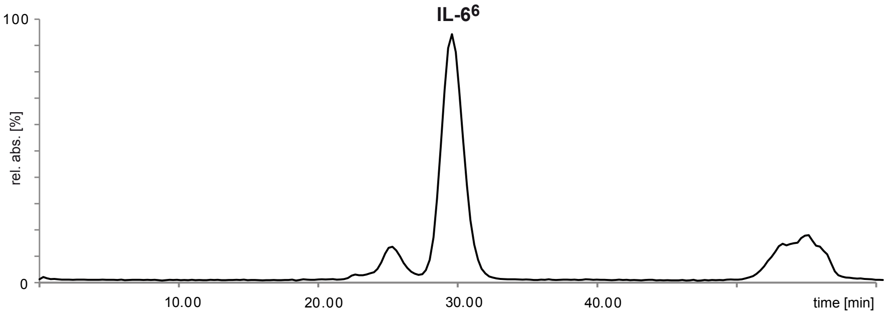

***Figure S60**: Purification of **IL-6^{6}** by gel filtration.*

Yield of **IL-6^{6} red**: 0.51 mg (0.02 µmol; 36 %; determined by A_{280}); ESI-MS: m/z (average isotopes): $C_{994}H_{1613}N_{259}O_{347}S_{8}$ (23000.49); calculated: $(M+18H)^{18+}$ 1278.81, $(M+17H)^{17+}$ 1353.97, $(M+16H)^{16+}$) 1438.53, found: 1279.16, 1354.33, 1438.94.

Yield of **IL-6^{6}**: 0.40 mg (0.02 µmol; 77 %; determined by A_{280}); ESI-MS: m/z (average isotopes): $C_{994}H_{1609}N_{259}O_{347}S_{8}$ (22996.45); calculated: $(M+18H)^{18+}$ 1278.58, $(M+17H)^{17+}$ 1353.73, $(M+16H)^{16+}$) 1438.28, found: 1279.61, 1354.74, 1439.43.

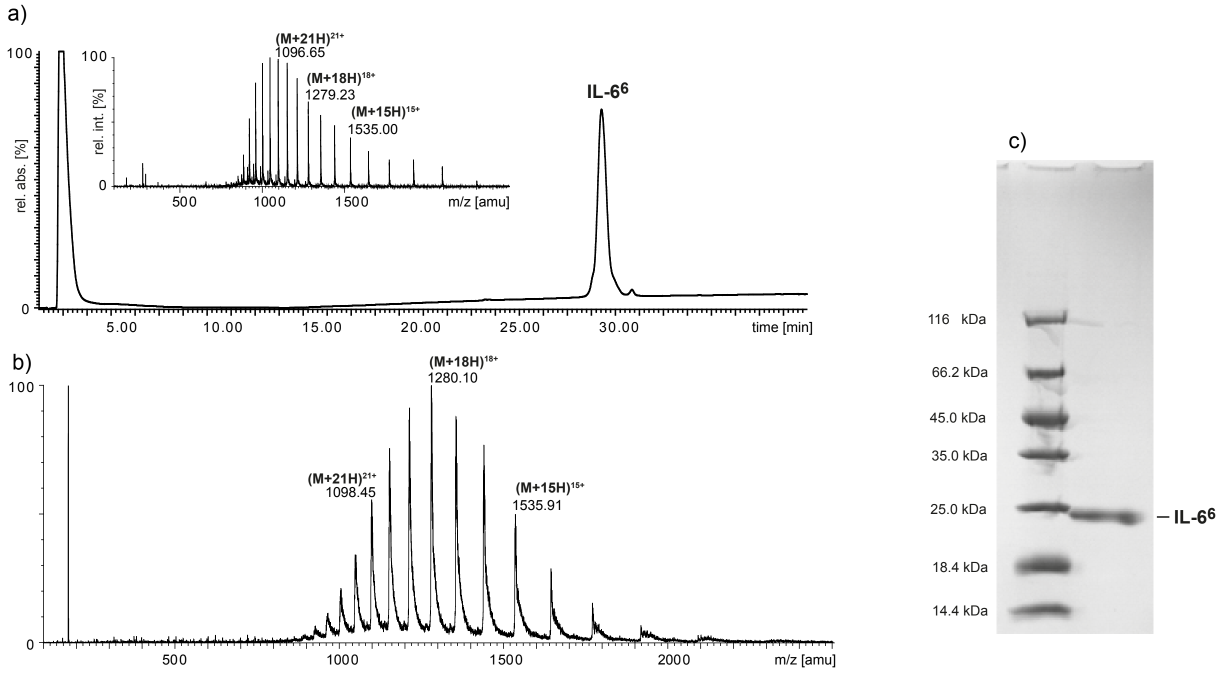

Figure S61: *Characterization of purified **IL-6^{6}**. a) HPLC-MS, b) ESI-MS (direct injection), c) SDS-PAGE*

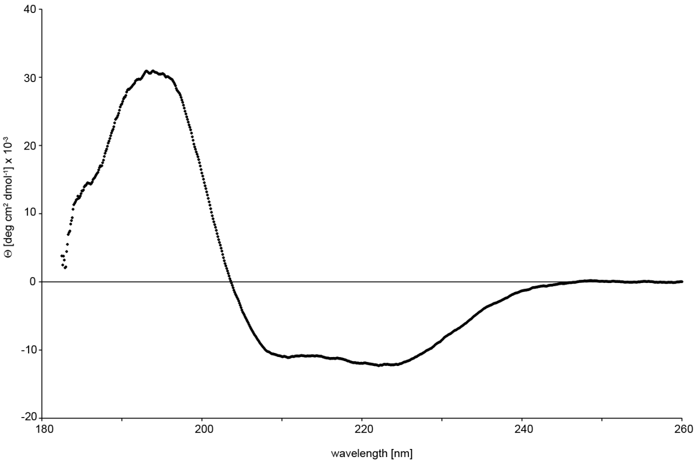

Figure S62: *CD-spectrum of purified **IL-6^{6}**.*

Synthesis of tetraantennary ***IL-6***^{7}

ligation buffer: 8 M GdmCl, 0.1 M Na_2HPO_4, 0.1 M MPAA, 15 mM TCEP, pH 7

refolding buffer: 0.5 M arginine, 150 mM NaCl, 50 mM Na_2HPO_4, pH 8.5

The reactions were performed in an anaerobic chamber. 1.3 mg (0.18 µmol) of a mixture of **E7**/**F7** and 3.0 mg (0.19 µmol) of **C** were dissolved in 200 µl of ligation buffer. The reaction was kept for seven days at ambient temperature. The ligation mixture was subsequently reduced by addition of 6.2 mg of DTT (final concentration 200 mM) and incubated for four hours.

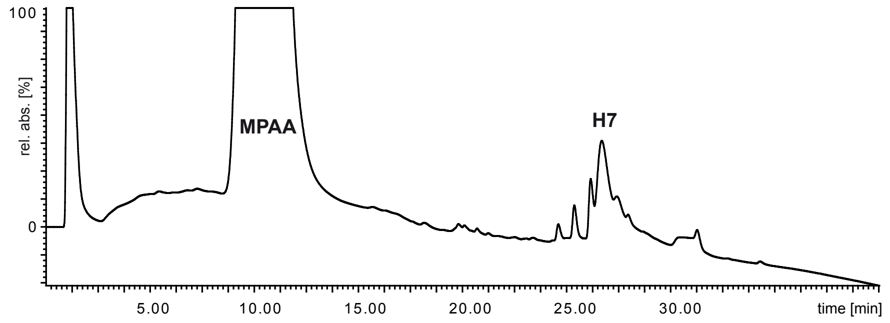

Figure S64*: HPLC-MS of the ligation of* ***E7****/****F7*** *with* ***C*** *after 7 days.*

The solution containing glycoprotein **H7** (total volume 200 µL) was refolded and purified in aliquots of 50 µL. A portion of 50 µL of the reduced ligation mixture was placed in a test tube and 950 µl of refolding buffer were added within three seconds during vortexing. After 30 min the test tube was removed from the anaerobic chamber, centrifuged and the supernatant was immediately passed over a HiLoad 16/60 Superdex 75 pg column (flow rate: 2 mL/min, refolding buffer).

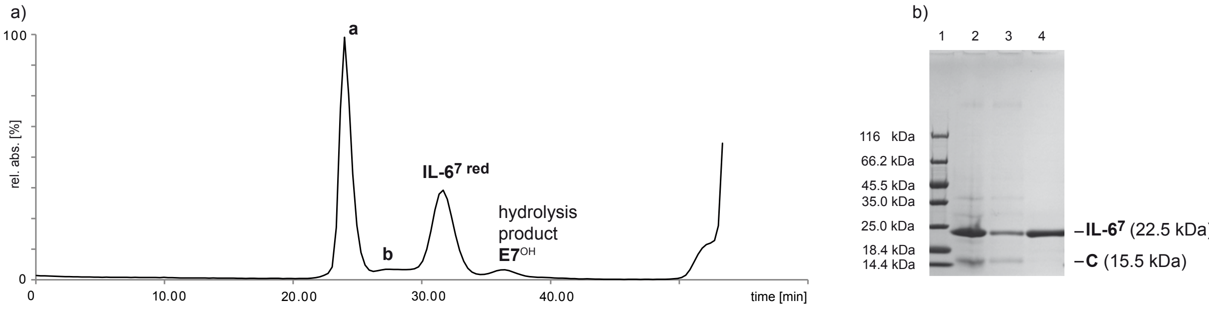

Figure S65*: a) Purification of refolded* ***IL-6***$^{7\ red}$ *by gel filtration; b) SDS-PAGE analysis of the purification of* ***IL-6***$^{7\ red}$ *by gel filtration. Lane 1: molecular weight standard; Lane 2: crude* ***IL-6***$^{7\ red}$*; Lane 3: Peak* ***a*** *at 22 min; Lane 4:* ***IL-6***$^{7\ red}$ *(32 min).*

The fraction of monomeric **IL-6^{7} red** (ca. 10 mL per 50 µL aliquot) was concentrated by ultrafiltration to 1 mL (Pierce concentrators, 20 mL, 20 K MWCO, 3800 g, 10 °C, Thermo Scientific, USA). The concentrate was transferred to a test tube and 1 µL of aqueous cysteamine (1 mM) were added. **IL-6^{7} red** was oxidized in the open tube for 4 days. Crude **IL-6^{7}** was purified over a HiLoad 16/60 Superdex 75 pg column (flow rate: 2 mL/min, refolding buffer). The fractions of monomeric **IL-6^{7}** (ca. 10 mL per 60 µL aliquot) were pooled and **IL-6^{7}** was concentrated by ultrafiltration as above.

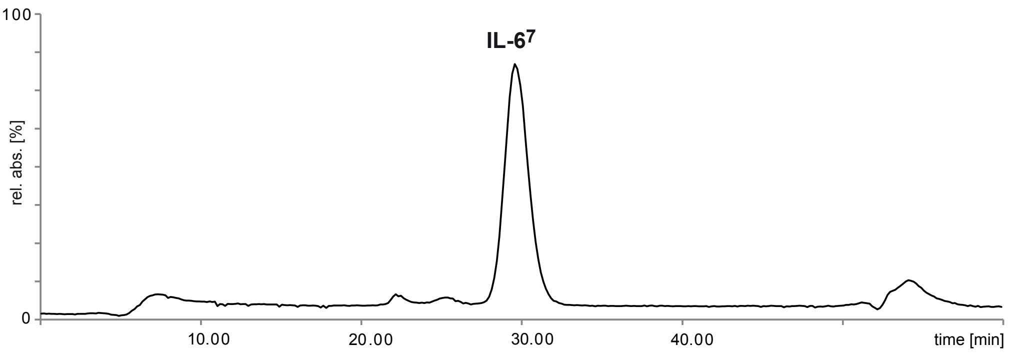

***Figure S66**: Purification of **IL-6^{7}** by gel filtration.*

Yield of **IL-6^{7} red**: 1.3 mg (0.06 µmol; 32 %; determined by A_{280}); ESI-MS: m/z (average isotopes): $C_{976}H_{1585}N_{259}O_{331}S_8$ (22500.08); calculated: $(M+18H)^{18+}$ 1251.00, $(M+17H)^{17+}$ 1324.53, $(M+16H)^{16+}$) 1407.26, found: 1251.16, 1324.66, 1407.29.

Yield of **IL-6^{7}**: 0.9 mg (0.04 µmol; 75 %; determined by A_{280}); ESI-MS: m/z (average isotopes): $C_{976}H_{1581}N_{259}O_{331}S_8$ (22496.04); calculated: $(M+18H)^{18+}$ 1250.78, $(M+17H)^{17+}$ 1324.30, $(M+16H)^{16+}$) 1407.00, found: 1250.98, 1324.51, 1407.23.

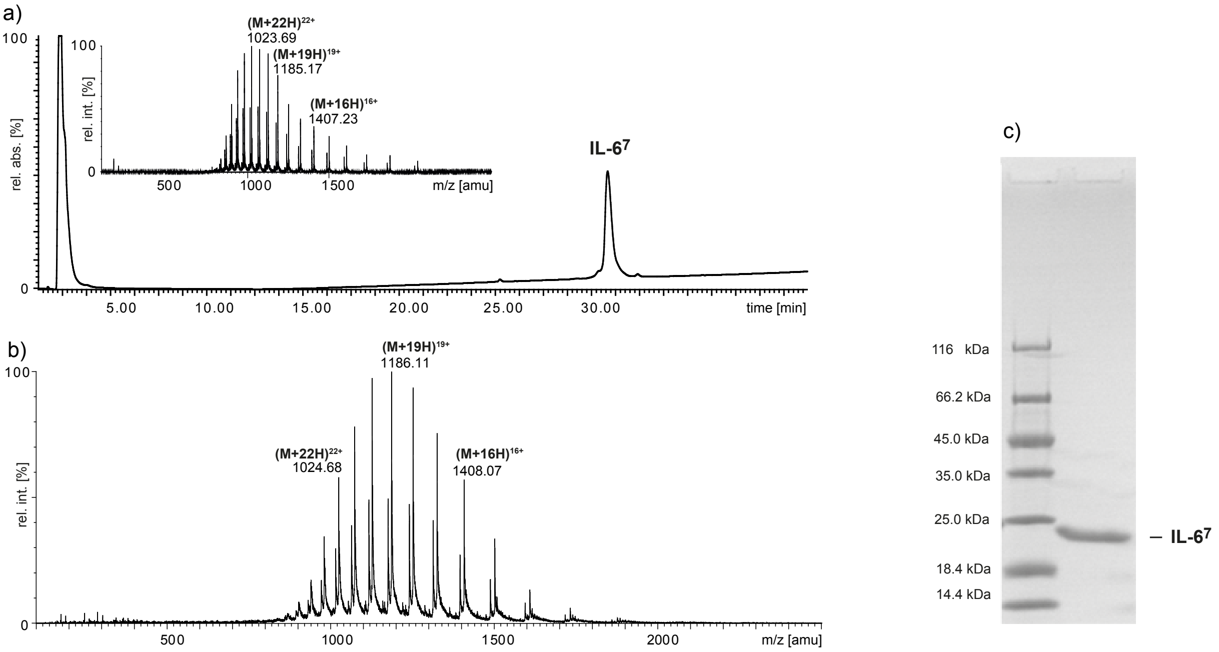

Figure S67: *Characterization of purified* ***IL-6*** ^{7}*. a) HPLC-MS, b) ESI-MS (direct injection), c) SDS-PAGE.*

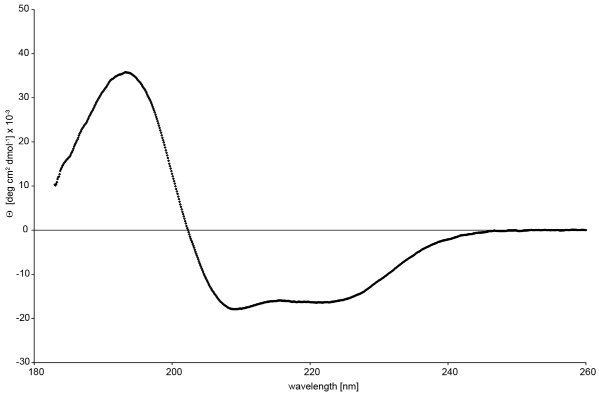

Figure S68: *CD-spectrum of purified* ***IL-6*** ^{7}*.*

Synthesis of tetraantennary galactosylated ***IL-6***^{8}

ligation buffer: 8 M GdmCl, 0.1 M Na_2HPO_4, 0.1 M MPAA, 15 mM TCEP, pH 7

refolding buffer: 0.5 M arginine, 150 mM NaCl, 50 mM Na_2HPO_4, pH 8.5

The reactions were performed in an anaerobic chamber. 1.0 mg (0.13 µmol) of a mixture of **E8**/**F8** and 2.0 mg (0.13 µmol) of **C** were dissolved in 150 µl of ligation buffer. The reaction was kept for seven days at ambient temperature. The ligation mixture was subsequently reduced by addition of 4.6 mg of DTT (final concentration 200 mM) and incubated for four hours.

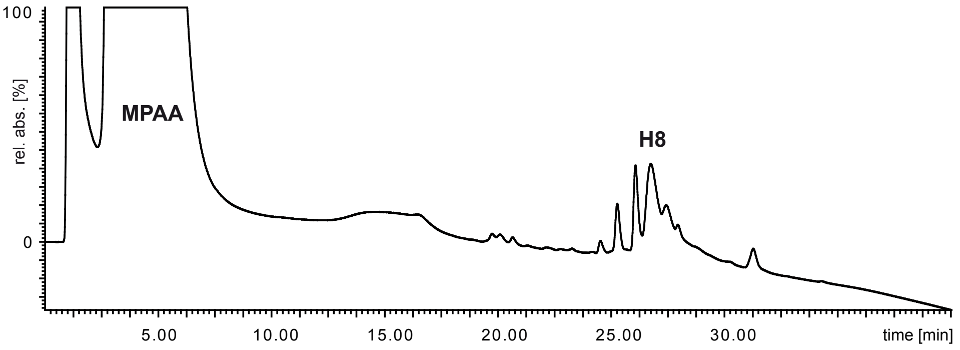

Figure S69: *HPLC-MS of the ligation of* ***E8/F8*** *with* ***C*** *after 7 days.*

The solution containing glycoprotein **H8** (total volume 150 µL) was refolded and purified in aliquots of 50 µL. A portion of 50 µL of the reduced ligation mixture was placed in a test tube and 950 µl of refolding buffer were added within three seconds during vortexing. After 30 min the test tube was removed from the anaerobic chamber, centrifuged and the supernatant was immediately passed over a HiLoad 16/60 Superdex 75 pg column (flow rate: 2 mL/min, refolding buffer).

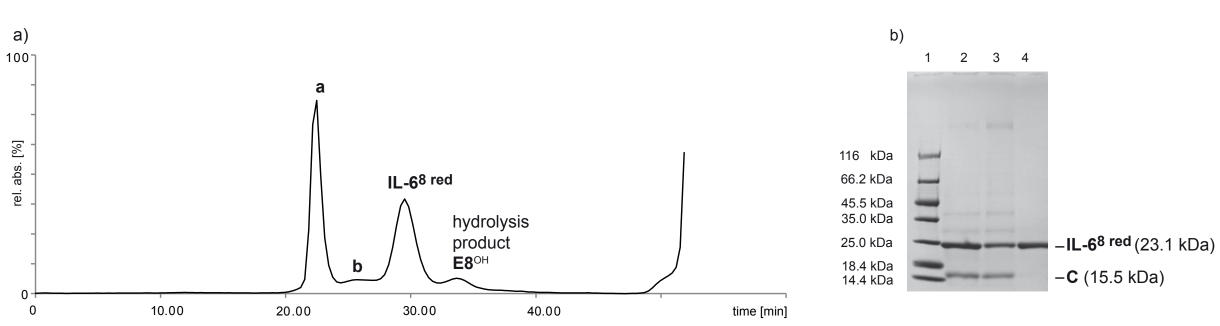

Figure S70: *a) Purification of refolded* ***IL-6***$^{8\ red}$ *by gel filtration; b) SDS-PAGE analysis of the purification of* ***IL-6***$^{8\ red}$ *by gel filtration. Lane 1: molecular weight standard; Lane 2: crude* ***IL-6***$^{8\ red}$*; Lane 3: Peak* ***a*** *at 22 min; Lane 4:* ***IL-6***$^{8\ red}$ *(29 min).*

The fraction of monomeric **IL-6^8 ^{red}** (ca. 10 mL per 50 µL aliquot) was concentrated by ultrafiltration to 1 mL (Pierce concentrators, 20 mL, 20 K MWCO, 3800 g, 10 °C, Thermo Scientific, USA). The concentrate was transferred to a test tube and 1 µL of aqueous cysteamine (1 mM) were added. **IL-6^8 ^{red}** was oxidized in the open tube for 4 days. Crude **IL-6^8** was purified over a HiLoad 16/60 Superdex 75 pg column (flow rate: 2 mL/min, refolding buffer). The fractions of monomeric **IL-6^8** (ca. 10 mL per 60 µL aliquot) were pooled and **IL-6^8** was concentrated by ultrafiltration as above.

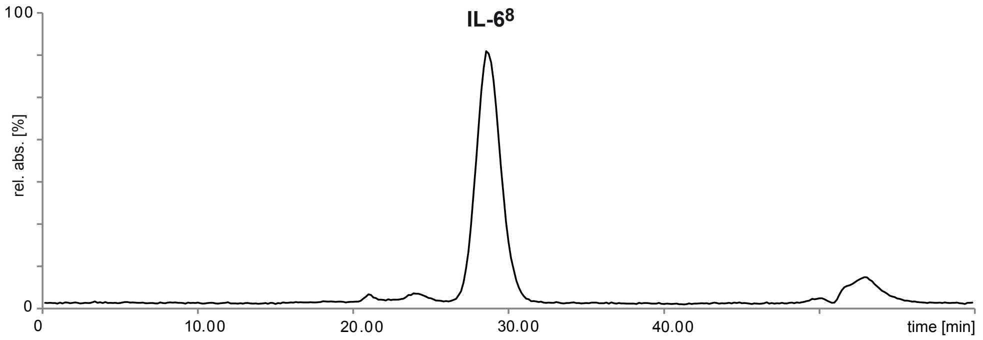

***Figure S71**: Purification of oxidized **IL-6^8** by gel filtration.*

Yield of **IL-6^8 ^{red}**: 1.2 mg (0.05 µmol; 40 %; determined by A_{280}); ESI-MS: m/z (average isotopes): $C_{1000}H_{1625}N_{259}O_{351}S_8$ (23148.65); calculated: $(M+18H)^{18+}$ 1287.04, $(M+17H)^{17+}$ 1362.69, $(M+16H)^{16+}$) 1447.79, found: 1287.02, 1362.78, 1447.76.

Yield of **IL-6^8**: 0.9 mg (0.04 µmol; 75 %; determined by A_{280}); ESI-MS: m/z (average isotopes): $C_{1000}H_{1621}N_{259}O_{351}S_8$ (23144.61); calculated: $(M+18H)^{18+}$ 1286.81, $(M+17H)^{17+}$ 1362.45, $(M+16H)^{16+}$) 1447.54, found: 1286.96, 1362.66, 1447.70.

ESI-HRMS: m/z (exact mass): $C_{1000}H_{1621}N_{259}O_{351}S_8$ (23130.4721); calculated for most abundant isotope peak: $(M+12H)^{12+}$ 1929.6329, $(M+13H)^{13+}$ 1781.2770, $(M+14H)^{14+}$ 1654.1149; found: 1929.6352, 1781.2786, 1654.1166

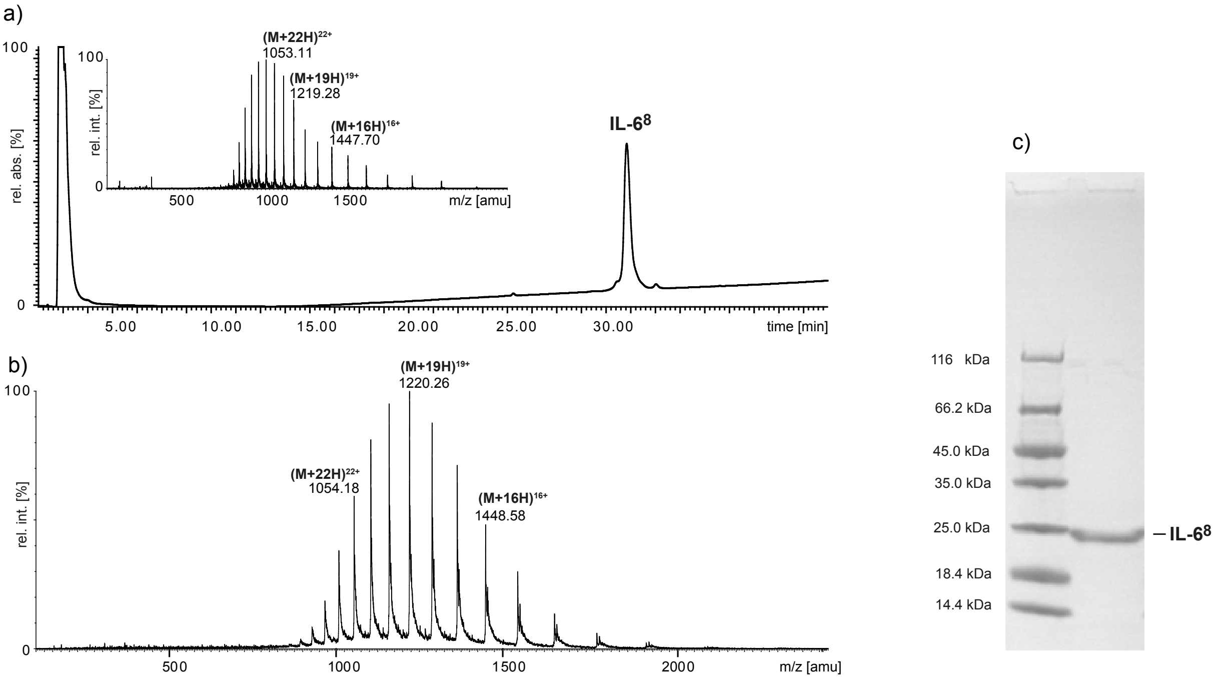

Figure S72*: Characterization of purified* ***IL-6***$^{\boldsymbol{8}}$. *a) HPLC-MS, b) ESI-MS (direct injection), c) SDS-PAGE*

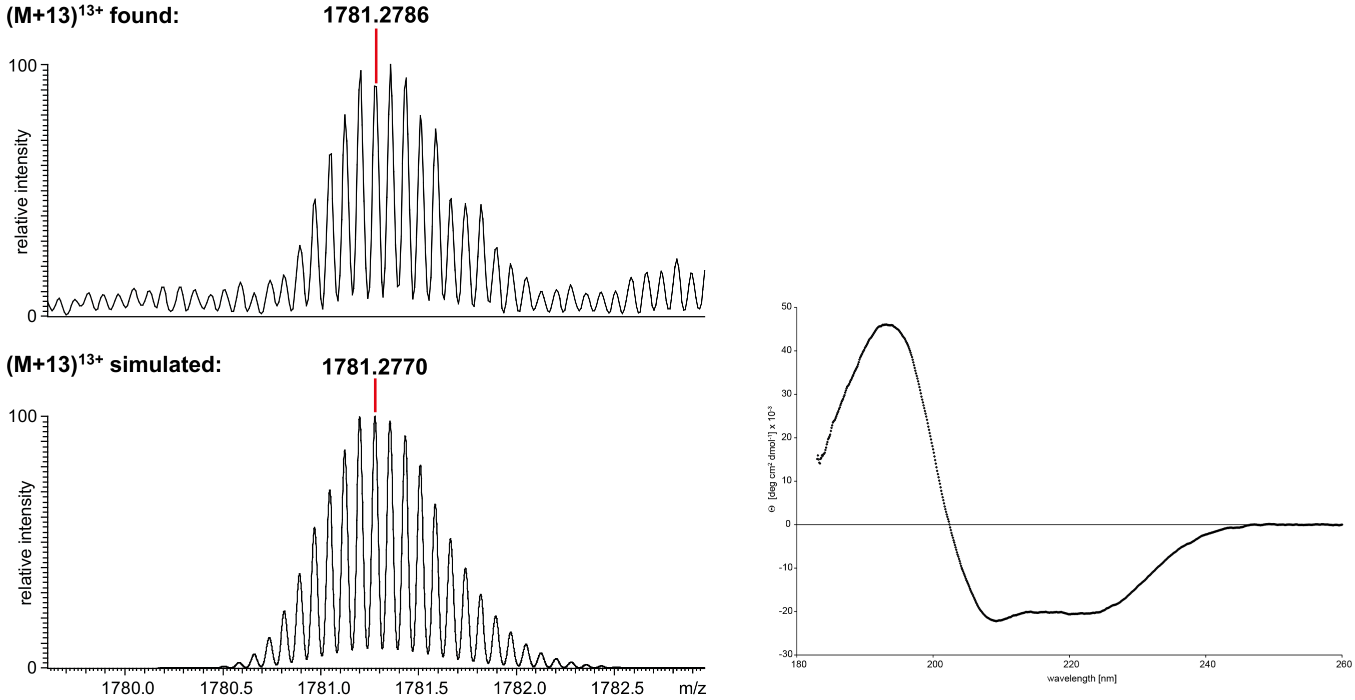

Figure S73*: a) Measured and simulated HR-MS* $(M+13)^{13+}$ *of* ***IL-6***$^{\boldsymbol{8}}$, *b) CD-spectrum of purified* ***IL-6***$^{\boldsymbol{8}}$.

*Synthesis of tetraantennary 2,6-sialylated **IL-6^{9}***

ligation buffer: 8 M GdmCl, 0.1 M Na_2HPO_4, 0.1 M MPAA, 15 mM TCEP, pH 7

refolding buffer: 0.5 M arginine, 150 mM NaCl, 50 mM Na_2HPO_4, pH 8.5

The reactions were performed in an anaerobic chamber. 1.9 mg (0.21 µmol) of a mixture of **E9**/**F9** and 3.5 mg (0.22 µmol) of **C** were dissolved in 200 µl of ligation buffer. The reaction was kept for six days at ambient temperature. The ligation mixture was subsequently reduced by addition of 6.2 mg of DTT (final concentration 200 mM) and incubated for four hours.

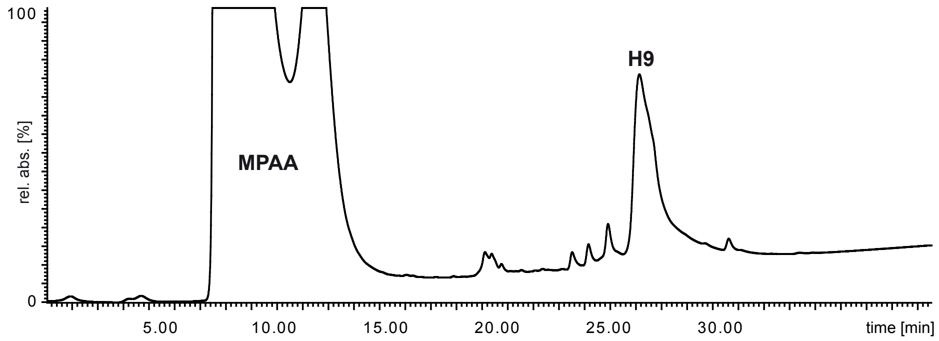

***Figure S74**: HPLC-MS of the ligation of **E9/F9** with **C** after 6 days.*

The solution containing glycoprotein **H9** (total volume 200 µL) was refolded and purified in aliquots of 50 µL. A portion of 50 µL of the reduced ligation mixture was placed in a test tube and 950 µl of refolding buffer were added within three seconds during vortexing. After 30 min the test tube was removed from the anaerobic chamber, centrifuged and the supernatant was immediately passed over a HiLoad 16/60 Superdex 75 pg column (flow rate: 2 mL/min, refolding buffer).

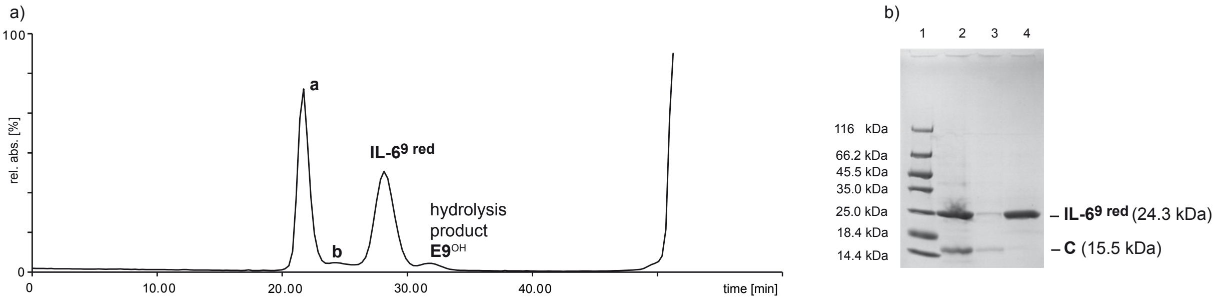

***Figure S75**: a) Purification of refolded **H9^{red}** by gel filtration; b) SDS-PAGE analysis of the purification of **H9^{red}** by gel filtration. Lane 1: molecular weight standard; Lane 2: crude **H9^{red}**; Lane 3: Peak **a** at 22 min; Lane 4: **H9^{red}** (29 min).*

The fraction of monomeric **IL-6^9 ^{red}** (ca. 10 mL per 50 µL aliquot) was concentrated by ultrafiltration to 1 mL (Pierce concentrators, 20 mL, 20 K MWCO, 3800 g, 10 °C, Thermo Scientific, USA). The concentrate was transferred to a test tube and 1 µL of aqueous cysteamine (1 mM) were added. **IL-6^9 ^{red}** was oxidized in the open tube for 4 days. Crude **IL-6^9** was purified over a HiLoad 16/60 Superdex 75 pg column (flow rate: 2 mL/min, refolding buffer). The fractions of monomeric **IL-6^9** (ca. 10 mL per 60 µL aliquot) were pooled and **IL-6^9** was concentrated by ultrafiltration as above.

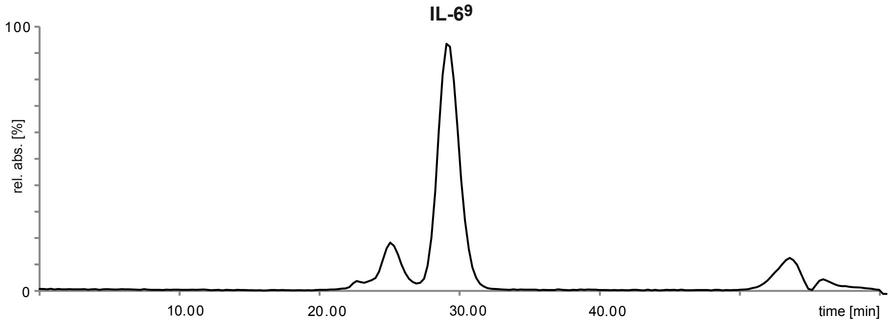

***Figure S76**: Purification of oxidized **H9** by gel filtration.*

Yield of **IL-6^9 ^{red}**: 2.5 mg (0.10 µmol; 48 %; determined by A280); ESI-MS: m/z (average isotopes): $C_{1044}H_{1693}N_{263}O_{383}S_8$ (24313.67); calculated: $(M+18H)^{18+}$ 1351.54, $(M+17H)^{17+}$ 1430.98, $(M+16H)^{16+}$) 1520.35, found: 1351.16, 1430.51, 1519.79.

Yield of **IL-6^9**: 1.8 mg (0.07 µmol; 73 %; determined by A280); ESI-MS: m/z (average isotopes): $C_{1044}H_{1689}N_{263}O_{383}S_8$ (24309.63); calculated: $(M+18H)^{18+}$ 1351.54, $(M+17H)^{17+}$ 1430.98, $(M+16H)^{16+}$) 1520.35, found: 1352.37, 1431.83, 1521.36.

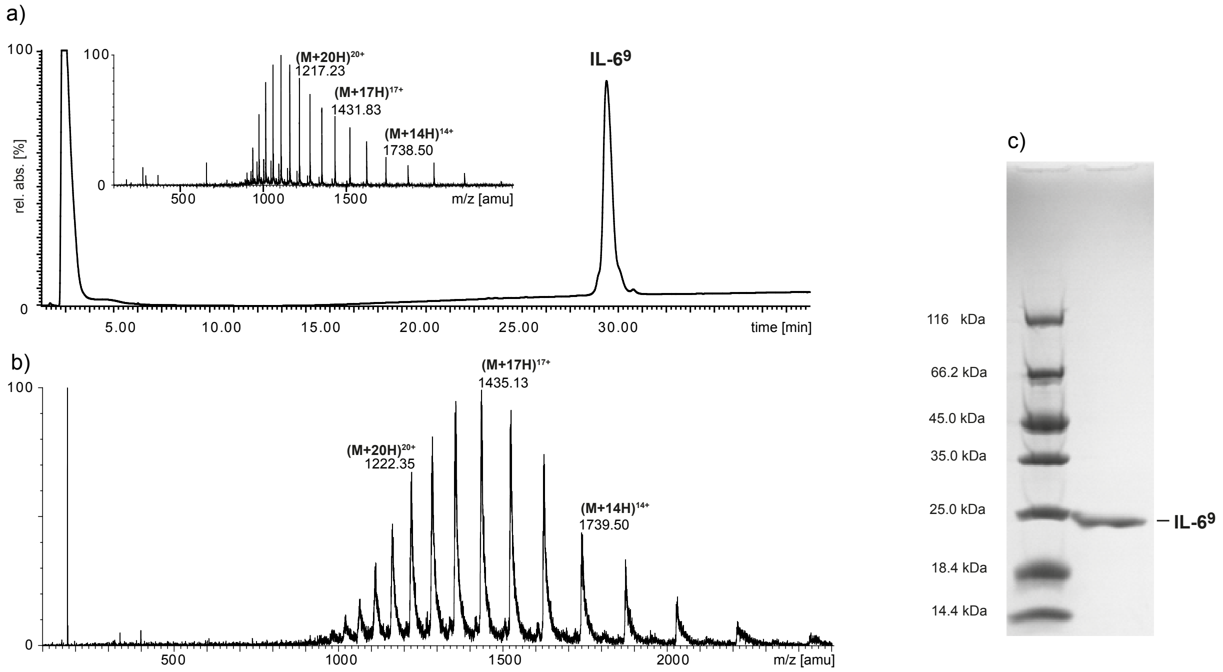

***Figure S77**: Characterization of purified **IL-6^9**. a) HPLC-MS, b) ESI-MS (direct injection), c) SDS-PAGE*

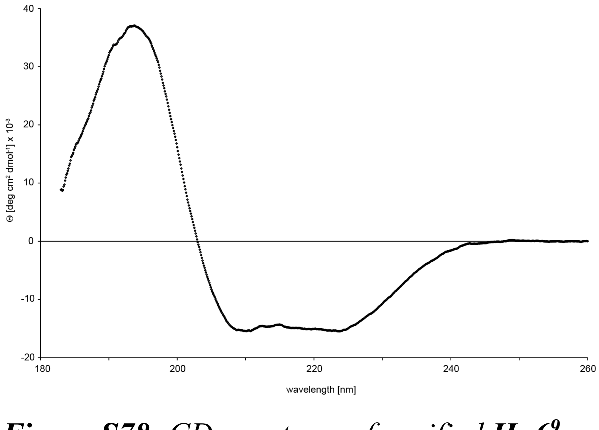

***Figure S78**: CD-spectrum of purified **IL-6^9**.*

15. CD spectroscopy

An aliquot of the final solutions of the **IL-6** glycoforms (50 µL) was exchanged to 10 mM sodium phosphate pH 7.4 (NAP-10 columns, GE Healthcare, Germany; procedure as suggested by the manufacturer). The CD spectra were recorded in a Hellma 165-QS cuvette (1.0 mm, 160 µL) with a J-600 spectropolarimeter (Jasco, Germany).

16. Bioassay for the measurement of IL-6 activity

Murine pre-B-cells (Ba/F3) stably transduced with cDNAs encoding human gp130 and human IL-6R (termed Ba/F3-gp130-IL-6R) have been described previously.[8] $5x10^3$ Ba/F3-gp130-IL-6R cells were suspended in DMEM containing 10% FBS and 1% Pen/Strep in a final volume of 100µl in a 96well plate. Cells were incubated in the absence or presence of increasing concentrations of either recombinant, unglycosylated IL-6, or the different glycoforms of IL-6 for 48h at 37°C with 5% CO_2 in a water-saturated atmosphere. Cell viability was measured using the Cell Titer Blue Cell viability assay (Promega, Karlsruhe, Germany) according to the manufacturer's protocol (excitation 560 nm, emission 590 nm). Normalization of relative light units (RLU) was achieved by subtraction of values measured at 0min from the values obtained after 60min. All values were measured in triplicates in each experiment.

17. Validation of ELISA test for IL-6 glycoforms

For the detection of the glycoforms **IL-6$^{1\text{-}9}$** and **IL-6** from *E. coli* we used a commercial research ELISA sandwich test specific for IL-6 in human serum (ImmunoTools, Friesoythe, Germany). An initial quantification of IL-6 glycoforms was performed as follows: A solution containing 250 pg of each glycoform was subjected to the assay according to the manufacturer's instructions.

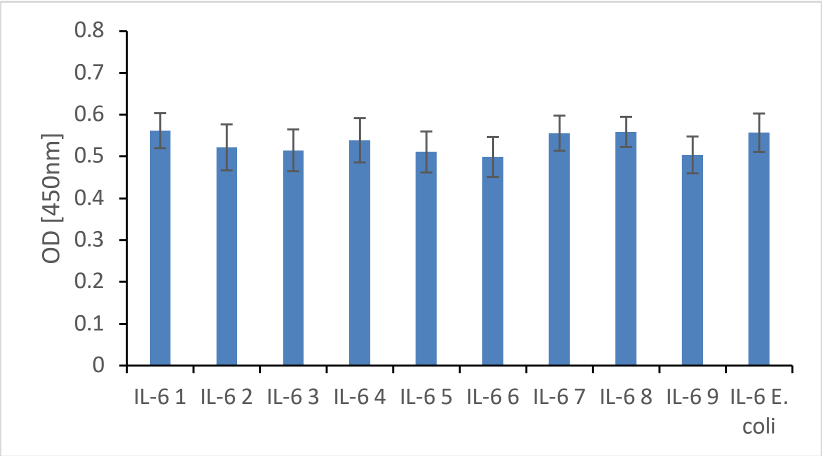

***Figure S79**: Results of ELISA sandwich assay for 250 pg of the glycoforms **IL-6$^{1\text{-}9}$** and **IL-6** from E. coli.*

18. Analysis of IL-6 plasma clearance in vivo

Rats which have been equipped with two vascular catheters were obtained from Charles River Laboratories. Rats (250-275 g body weight) were housed under standard conditions with food and drinking water ad libitum and held under a 12h light-dark cycle. Animals had one week to adjust after arrival before the experiment started.

The IL-6 glycoforms were shipped as solutions in the buffer used for the final gel filtration. The concentration of each glycoform was determined by absorption at 280 nm as specified in the supporting information. The solutions for injection were prepared by diluting the stocks with PBS to a final concentration of 32 µg of IL-6 glycoform/mL. Aliquots of 250 µL were made and kept at -24 °C until use.

Rats were injected with one of the IL-6 glycoforms (8 µg of IL-6 in 250 µl of sterile PBS) in one catheter and blood samples were obtained from the second catheter 0, 1, 2, 5, 10, 15, and 20 minutes after injection of IL-6. Serum was obtained from the coagulated blood samples via centrifugation, and serum samples were stored until measurement at -20°C. IL-6 was determined via a human IL-6 specific ELISA at appropriate dilution according to the manufacturer's instructions (ImmunoTools, Friesoythe, Germany). 24h after the IL-6 injection, the final blood sample for the determination of acute phase proteins was drawn, which was treated as the other blood samples described above. One week after the first experiment, rats received a different IL-6 glycoform and were treated according to the same protocol. Rats were used 3 to 4 times, depending on the patency rate of the catheter. After the last experiments, rats were sacrificed and their livers harvested for determination of induction of genes encoding acute phase response proteins. All animal experiments were approved by the local authorities (Ministerium für Energiewende, Landwirtschaft, Umwelt und ländliche Räume, Kiel, Germany, V 242-7224.121-3 (47-4/15)).

19. Quantitative Real-Time PCR (qPCR)

Rat livers were excised, snap-frozen in liquid nitrogen and total RNA was isolated using the Nucleospin RNA II kit (Macherey-Nagel, Düren, Germany) according to the manufacturer's instructions. 1 µg of the total RNA was reversely transcribed with oligo-dT15 primers using the RevertAid reverse transcriptase (Thermo Fisher Scientific, Waltham, MA, USA). The

amount of cDNA derived from 50 ng of RNA was used for each qPCR reaction using the Power SYBR Green PCR Master Mix (Thermo Fisher Scientific, Waltham, MA, USA) according to manufacturer's instructions on a LightCycler® 480 System (Roche Applied Systems, Penzberg, Germany). One sample from each of the eight rats was measured in duplicates and normalized to GAPDH expression. Relative gene expression was calculated using the $2^{-\Delta Ct}$ method. The following primers were used:

ORM1_fwd: GTGTGCAGGAGCAGTGAAAA, rORM1_rev: CATGCCCACATCTTTGACAG,
rA2M_fwd: GGCCATTGCCTATCTCAATACG, rA2M_rev: TTATCCCCAAAGGCGCTGTA,
rCRP_fwd: GTGCTATCTCCAGAACAG, rCRP_rev: CACAACAGTCAGTCAAGG,
rFGL_fwd: GGTGGGCTAGTCACCAAACA, rFGL_rev: CCTTGGTAGTACACGCCGTT,
rHP_fwd: TGATCAAGCTCAAACAGAAAGTG, rHP_rev: CATAGCAAGTGTCTTCCTCATACTT,
rCP_fwd: GTATGTGATGGCTATGGGCAA, rCP_rev: CCTGGATGGAACTGGTGATGGA
rIL6_fwd: TCTCTCCGCAAGAGACTTCCA, rIL6_rev: ATACTGGTCTGTTGTGGGTGG,
rGAPDH_fwd:TGACTCTACCCACGGCAAGTTCAA, rGAPDH_rev: ACGACATACTCAGCACCAGCATCA

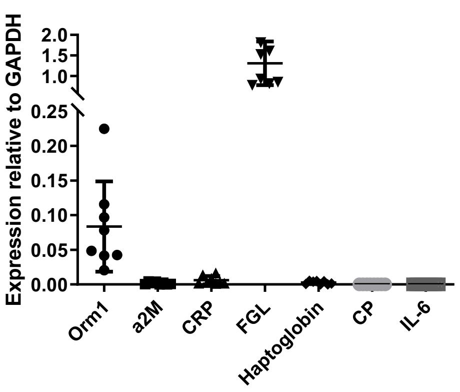

Figure S 80: *Quantitative Real-Time PCR (qPCR) of the rat livers tested for Orm1 (rat alpha1-acid glycoprotein); a2M (rat alpha2-macroglobulin); CRP (rat C-reactive protein); FGL (rat fibrinogen like protein 1); rat haptoglobin; CP (rat ceruloplasmin); IL-6. The expressions were normalized to GADPH (rat glyceraldehyde 3-phosphate dehydrogenase).*

20. Quantification of rat acute phase proteins

The levels of the acute phase proteins (rat C-reactive protein and rat alpha1-acid glycoprotein) in the sera of rats injected with the different **IL-6** variants were detected using specific ELISA Kits (Abcam, Berlin, Germany), which were used according to the manufacturer's instructions.

a) Serum levels (0h, 24h) of rat C-reactive protein (rCRP) after administration of **IL-6** variants.

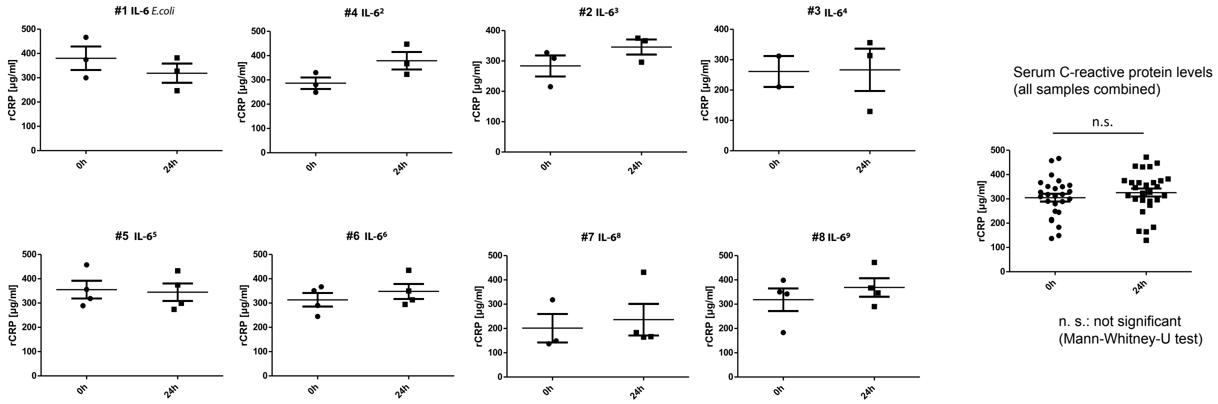

b) Serum levels (0h, 24h) of rat alpha1-acid glycoprotein after administration of **IL-6** variants.

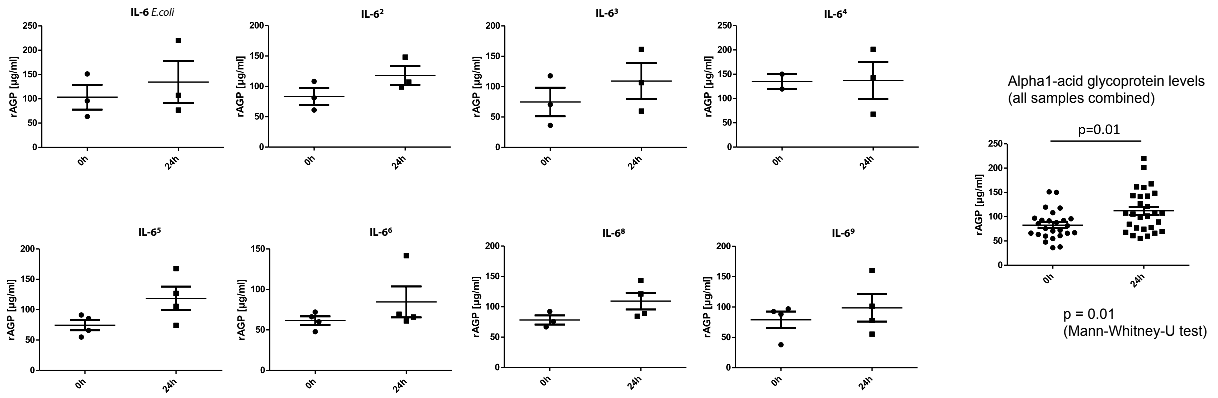

Figure S 81: *Determination of serum concentrations of a) rat C-reactive protein and b) rat alpha1-acid glycoprotein before (0h) and after (24h) administration of specific* ***IL-6*** *variants. The increase of serum concentration for C-reactive protein was statistically not significant whereas for alpha1-acid glycoprotein an increase was found (Mann-Whitney-U test).*

21. Complexation of IL-6 and IL-6 glycoforms with sIL-6R

Complexes of IL-6 with the IL-6 receptor were formed using recombinantly expressed soluble IL-6 receptor (sIL-6R).[9] For complexation 15 µg (0.4 nmol) of sIL-6R in 24 µL of PBS (154 mM NaCl, 5.6 mM Na_2HPO_4, 1.1 mM KH_2PO_4, pH 7.4) were mixed with 1.2 nmol of

each IL-6 variant. The amounts used were 22.5 µL (1.1 mg/mL) of **IL-6** (*E. coli*) in PBS; 23 µL (1.1 mg/mL) of **IL-6^3** in Arg-buffer (0.5 M L-Arg, 150 mM NaCl, 50 mM Na_2HPO_4, pH 8.5 and 29 µL (0.9 mg/mL) of **IL-6^8** in Arg-buffer. The mixtures were vortexed immediately and kept for 10 minutes at room temperature. Subsequently, the IL-6/sIL-6R complexes were purified by gel filtration (Superdex 200 Increase 3.2/300, flow rate: 40 µL/min, PBS). The fractions containing IL-6/sIL-6R complexes were collected and kept at 4° C.

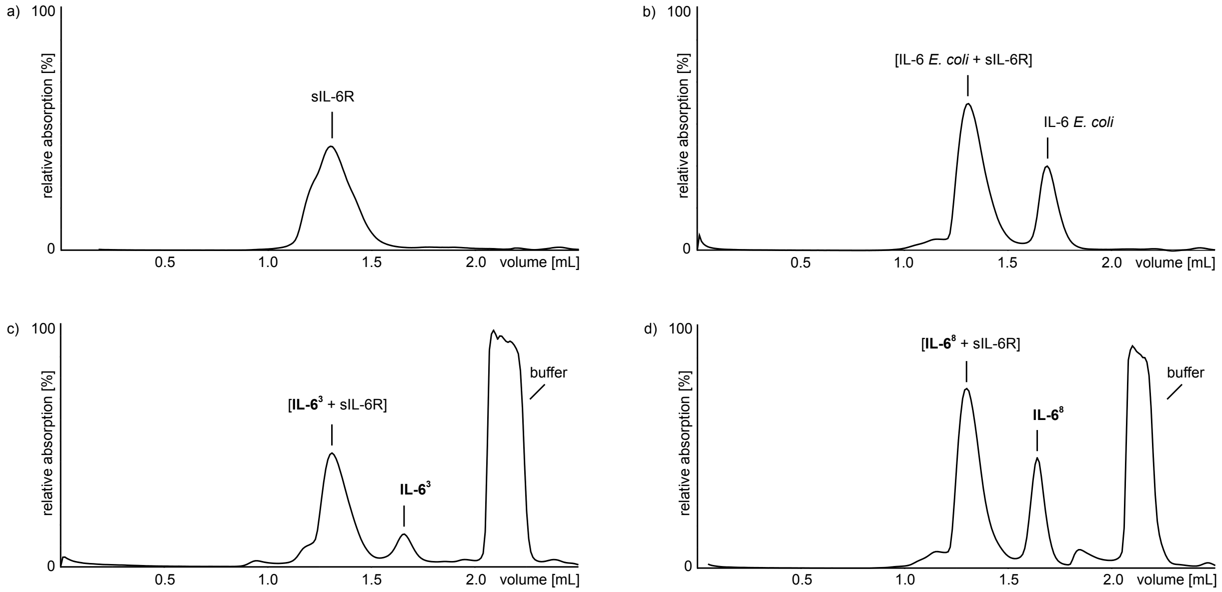

***Figure S82**: SEC of IL-6/sIL-6R complexes: a) soluble IL-6 receptor (sIL-6R) only; b) sIL-6R loaded with **IL-6** expressed in E. coli; c) sIL-6R loaded with **IL-6^3**; d) sIL-6R loaded with **IL-6^8**.*

22. Biolayer interferometry (BLI)

The Octet RED96 system (ForteBio, Pall Life Science, Fremont, CA, USA) was employed for binding kinetic measurements. All steps were performed in a final volume of 200 µl at 25° C and 1000 rpm agitation. The ligand sGP130Fc (IL-6 Coreceptor GP130-Fc Fusion)[10] was immobilized on anti-human Fc biosensors (ForteBio) at 5 µg/mL in PBS (Sigma Aldrich) for 180 s. Subsequently, the tips were rinsed in PBS for 45 s. The association of the three IL-6/sIL-6R complexes (different concentrations in PBS, see Fig. S82) was measured for 300 s followed by dissociation for 3600 s (plain PBS). In each experiment a reference control was measured by incubating the captured sGP130Fc with PBS. Data fitting and analysis was performed with ForteBio data analysis software using a 1:1 model after Savitzky-Golay filtering.

analyte	ligand (immobilized)	K_d (nM)	k_{on} (1/Ms)	k_{off} (1/s)	Rmax (saturation)
**IL-6** *E.coli*/sIL-6R complex	sGP130-Fc	0.408	1.58E+5 (± 8.59E+2)	6.45E-5 (± 2.75E-7)	0.2318
**IL-6^{3}**/sIL-6R complex	sGP130-Fc	0.402	1.45E+5 (± 4.08E+2)	5.84E-5 (± 1.28E-7)	0.3104
**IL-6^{8}**/sIL-6R complex	sGP130-Fc	0.192	1.53E+5 (± 4.48E+2)	2.93E-5 (± 1.57E-7)	0.2845

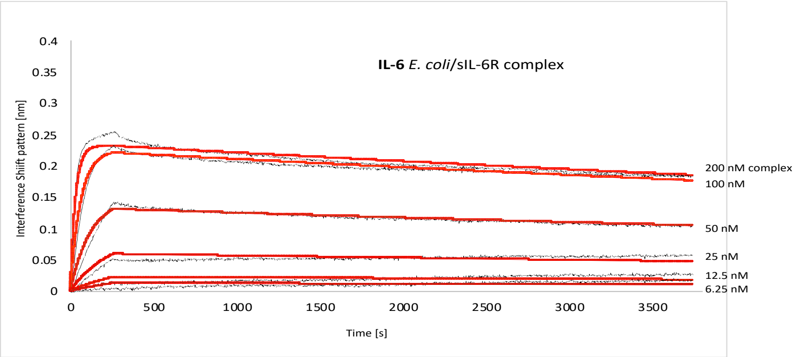

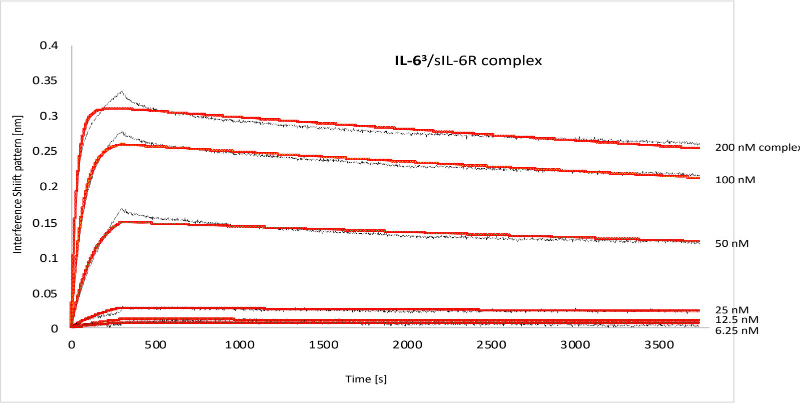

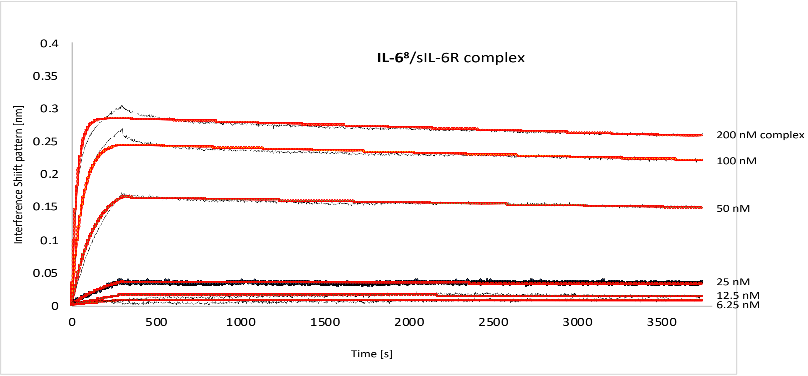

Figure S83*: BLI sensorgrams (black) and calculated curve fit (red) of three IL-6/sIL-6R complexes (at different concentrations) binding to immobilized sGP130-Fc. The calculated kinetic values are given in the table.*

23. Crystallization and X-ray structure determination

Synthetic **IL-6[1]** (GlcNAc)[1] was used at concentrations of 15 and 7.5 mg/ml for the screening of initial crystallization conditions by using the commercial Qiagen screen, JCSG+. Equal volumes of 300 nl of protein and screening solutions were pipette by a Phoenix liquid handling robot (Art Robbins) into 96-well MRC-2 crystallization plates (Molecular Dimensions) and then stored at 293 K, in a RockMaker-1000 imaging system (Formulatrix), and at 277 K. Initial 3D crystals appeared within days to weeks only at 277 K in a condition consisting of 0.24 M sodium malonate, pH 7.0 and 20% (w/v) PEG 3350. After optimization of the initial condition in 48-well MRC crystallization plates, a larger 3D crystal was transferred to a cryo-protectant solution of 0.2 M sodium malonate, pH 7.2, 18% (w/v) PEG 3350 and 25% (v/v) glycerol, flash-frozen and stored in liquid nitrogen. Diffraction measurements were carried out at the MX-14.1 beamline at Helmholtz-Zentrum Berlin. A complete data set was recorded, processed and scaled with with XDSAPP3[12] up to 2.0 Å resolution. Phase determination was performed by Molecular Replacement with PHASER[13] using 1ALU (PDB ID) as a start model. Model building and refinement were performed by REFMAC[14] and COOT[15]. Data processing, refinement and model statistics are given in Table S1.

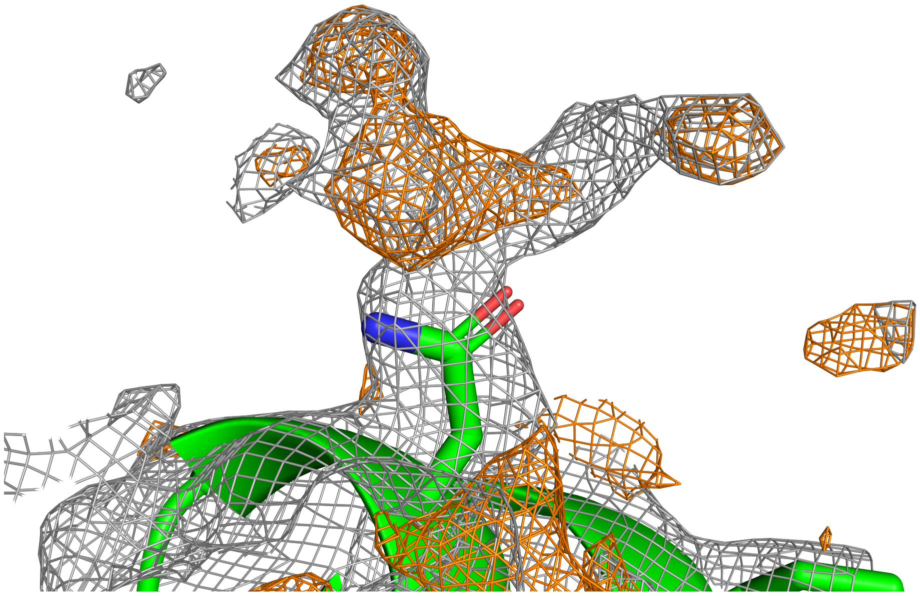

***Figure S84**: SigmaA-weighted 2mFo-DFc model (0.75 r.m.s.d., gray) and mFo-DFc residual (2.25 r.m.s.d., orange) densities of the final model of **IL-6[1]** at the ASN-44 glycosylation site. Model and density calculations did not contain any sugar atoms. Both densities indicate a covalent extension of the Asn side chain.*

**Table S1**: Crystallographic data statistics	
**Data processing**	
space group (symbol / no.)	C222(1) / 20
Unit cell (a,b,c ; α,β,γ) [Å / °]	72.10, 129.02, 50,10 ; 90,90,90
Resolution (High res), [Å]	50 – 2.0 (2.12 – 2.0)
Unique reflections (High res), [Å]	16029 (2465)
Observations (High res), [Å]	57915 (8326)
Completness (h.r.)	98.6 (95.9)
Mutliplicity (h.r.)	3.64 (3.69)
Wilson B-factor, [$Å^2$]	46.02
R-meas (h.r.), [%]	8.4 (167.0)
I/sigma (h.r.)	11.2 (0.72)
CC1/2 (h.r.)	99.9 (43.4)
**Refinement**	
no. of amino acids (aa range)	158 (16-50, 60-183)
no. of protein atoms	1,352
no. of peptide/intermediate atoms	0
no. of ligand atoms	0
no. of waters	83
no. of solvent atoms	0
no. of metals	0
Resolution (High res), [Å]	39.57 - 2.0 (2.05 - 2.0)
R-work (High res) [%]	20.6 (54.0)
R-free (High res) [%]	25.3 (46.9)
B-factor protein atoms, [Å2]	57.16
B-factor peptide/intermediate, [Å2]	--
B-factor ligands (incl. Zn2+), [Å2]	--
B-factor water, [Å2]	54.79
B-factor solvent atoms, [Å2]	--
RMSD bond lengths / target [A]	0.012 / 0.013
RMSD angles / target [°]	1.647 / 1.650
RMSD planes / target [A]	0.007 / 0.020
CC ligand (s)	--

References:

[1] A. Reif, S. Siebenhaar, A. Tröster, M. Schmälzlein, C. Lechner, P. Velisetty, K. Gottwald, C. Pöhner, I. Boos, V. Schubert, S. Rose-John, C. Unverzagt, *Angew. Chem. Int. Ed.* **2014**, *53*, 12125 –12131.

[2] M. Kiyozumi, K. Kato, T. Komori, A. Yamamoto, T. Kawasaki, H. Tsukamoto, *Carbohydr. Res.* **1970**, *14*, 355 – 364.

[3] V. Ullmann, M. Rädisch, I. Boos, J. Freund, C. Pöhner, S. Schwarzinger, C. Unverzagt, *Angew. Chem. Int. Ed.* **2012**, *51*, 11566-11570.

[4] D. Ott, J. Seifert, I. Prahl, M. Niemietz, J. Hoffmann, J. Guder, M. Mönnich, C. Unverzagt, *Eur. J. Org. Chem*. **2012**, 5054-5068

[5] M. Mönnich, S. Eller, T. Karagiannis, L. Perkams, T. Luber, D. Ott, M. Niemietz, J. Hoffman, J. Walcher, L. Berger, M. Pischl, M. Weishaupt, C. Wirkner, R. G. Lichtenstein, C. Unverzagt, *Angew. Chem. Int. Ed.*, **2016**, *55*, 10487–10492.

[6] C. G. F. Graf, C. Schulz, M. Schmälzlein, C. Heinlein, M. Mönnich, L. Perkams, M. Püttner, I. Boos, M. Hessefort, J. N. Lombana Sanchez, M. Weyand, C. Steegborn, B. Breiden, K. Ross, G. Schwarzmann, K. Sandhoff, C. Unverzagt *Angew. Chem. Int. Ed.*, **2017**, *56*, 5252-5257.

[7] A. Canales, I. Boos, L. Perkams, L. Karst, T. Luber, T. Karagiannis, G. Domínguez, F. J. Cañada, J. Pérez-Castells, D. Häussinger, C. Unverzagt, J. Jiménez-Barbero "Breaking the Limits in Analyzing Carbohydrate Recognition by NMR Spectroscopy: *Angew. Chem. Int. Ed.*, **2017**, *56*, 14987 –14991.

[8] C. Garbers, W. Thaiss, G. W. Jones, G. H. Waetzig, I. Lorenzen, F. Guilhot, R. Lissilaa, W. G. Ferlin, J. Grotzinger, S. A. Jones, S. Rose-John, J. Scheller, *J. Biol. Chem.* **2011**, *286*, 42959-42970.

[9] P. Vollmer, B. Oppmann, N. Voltz, M. Fischer, S. Rose-John, *Eur. J. Biochem.* **1999**, *263*, 438-446.

[10] T. Jostock, J. Mullberg, S. Ozbek, R. Atreya, G. Blinn, N. Voltz, M. Fischer, M. F. Neurath, S. Rose-John, *Eur. J. Biochem.* **2001**, *268*, 160-167.

[11] a) K. M. Sparta, M. Krug, U. Heinemann, U. Mueller, M. S. Weiss, *J. Appl. Cryst.* **2016**, *49*, 1085–1092; b) W. Kabsch, *Acta Crystallogr. D Biol. Crystallogr.* **2010**, *66*, 125-132.

[12] Sparta K., Krug M., Heinemann U., Mueller U., Weiss M. S. *J. Appl. Crystallogr.* **2016**, *49*, 1085-1092.

[13] A. J. McCoy, R. W. Grosse-Kunstleve, P. D. Adams, M. D. Winn, L. C. Storoni, R. J. Read, *J. Appl. Crystallogr.* **2007**, *40*, 658-674.

[14] G. N. Murshudov, P. Skubak, A. A. Lebedev, N. S. Pannu, R. A. Steiner, R. A. Nicholls, M. D. Winn, F. Long, A. A. Vagin, *Acta Crystallogr. D Biol. Crystallogr.* **2011**, *67*, 355-367.

[15] P. Emsley, B. Lohkamp, W. G. Scott, K. Cowtan, *Acta Crystallogr. D Biol. Crystallogr.* **2010**, *66*, 486-501.
